# Computed Tomography-derived intratumoral and peritumoral radiomics in predicting EGFR mutation in lung adenocarcinoma

**DOI:** 10.1007/s11547-023-01722-6

**Published:** 2023-09-25

**Authors:** Youlan Shang, Weidao Chen, Ge Li, Yijie Huang, Yisong Wang, Xiaoyan Kui, Ming Li, Hairong Zheng, Wei Zhao, Jun Liu

**Affiliations:** 1 Department of Radiology, The Second Xiangya Hospital, Central South University, No. 139 Middle Renmin Road, Changsha, 410011 Hunan People’s Republic of China; 2 Infervision, Chaoyang District, Beijing, 100025 China; 3 Department of Radiology, Xiangya Hospital, Central South University, No. 87 Xiangya Rd, Changsha, 410008 Hunan People’s Republic of China; 4 School of Computer Science and Engineering, Central South University, Changsha, 410083 Hunan People’s Republic of China; 5 Department of Radiology, Huadong Hospital Affiliated to Fudan University, Shanghai, People’s Republic of China; 6 Paul C. Lauterbur Research Center for Biomedical Imaging, Institute of Biomedical and Health Engineering, Shenzhen Institutes of Advanced Technology, Chinese Academy of Sciences, Shenzhen, 518055 People’s Republic of China; 7 Clinical Research Center for Medical Imaging in Hunan Province, Changsha, Hunan Province People’s Republic of China

**Keywords:** Lung adenocarcinoma, CT, Radiomics, Peritumoral, EGFR

## Abstract

**Objective:**

To investigate the value of Computed Tomography (CT) radiomics derived from different peritumoral volumes of interest (VOIs) in predicting epidermal growth factor receptor (EGFR) mutation status in lung adenocarcinoma patients.

**Materials and methods:**

A retrospective cohort of 779 patients who had pathologically confirmed lung adenocarcinoma were enrolled. 640 patients were randomly divided into a training set, a validation set, and an internal testing set (3:1:1), and the remaining 139 patients were defined as an external testing set. The intratumoral VOI (VOI_I) was manually delineated on the thin-slice CT images, and seven peritumoral VOIs (VOI_P) were automatically generated with 1, 2, 3, 4, 5, 10, and 15 mm expansion along the VOI_I. 1454 radiomic features were extracted from each VOI. The *t*-test, the least absolute shrinkage and selection operator (LASSO), and the minimum redundancy maximum relevance (mRMR) algorithm were used for feature selection, followed by the construction of radiomics models (VOI_I model, VOI_P model and combined model). The performance of the models were evaluated by the area under the curve (AUC).

**Results:**

399 patients were classified as EGFR mutant (EGFR+), while 380 were wild-type (EGFR−). In the training and validation sets, internal and external testing sets, VOI4 (intratumoral and peritumoral 4 mm) model achieved the best predictive performance, with AUCs of 0.877, 0.727, and 0.701, respectively, outperforming the VOI_I model (AUCs of 0.728, 0.698, and 0.653, respectively).

**Conclusions:**

Radiomics extracted from peritumoral region can add extra value in predicting EGFR mutation status of lung adenocarcinoma patients, with the optimal peritumoral range of 4 mm.

**Supplementary Information:**

The online version contains supplementary material available at 10.1007/s11547-023-01722-6.

## Introduction

With the advancement of precision medicine, molecular targeted therapy has been widely used in the treatment of lung cancer. Several studies have shown that the epidermal growth factor receptor (EGFR) mutation status provides the conditions for individualized therapy in lung adenocarcinoma patients [1–4]. EGFR-mutant patients treated with the EGFR-tyrosine kinase inhibitor (EGFR-TKI) achieve longer progression-free survival and better response rates than conventional chemotherapy [5, 6]. Therefore, the National Comprehensive Cancer Network guidelines recommend routine detection of EGFR mutations to guide molecularly targeted therapy for lung adenocarcinoma patients [7].

Conventional identification of EGFR mutations requires biopsy and genetic testing which has several limitations in clinical practice: (1) the potential risk of tumor metastasis during biopsy; (2) the difficulty in obtaining representative tumor tissue due to tumor genetic heterogeneity; (3) not all tumors of all sizes and locations are suitable for biopsy; (4) a lack of adequate material and high-quality DNA may lead to testing failure; (5) genetic mutations may change throughout treatment, while repeated biopsies are impractical. In addition, the economic and time costs of biopsy should also be considered [8–11]. Therefore, there is an urgent need for a reliable, safe, convenient, and cost-effective method for the non-invasive prediction of EGFR mutation status in lung adenocarcinoma patients, to assist clinicians in selecting appropriate patients for EGFR-TKI treatment, support individualized decision-making, maximize the prognosis of the patient, and also avoid waste of medical resources.

As an emerging data mining technique, radiomics has attracted increasing attention for its advantages in providing objective and quantifiable imaging information, which can be used for differential diagnosis, genetic analysis, clinical staging, therapeutic evaluation, and prognosis prediction. The main steps of radiomics analysis are as follows: (1) acquisition and pre-processing of medical images (CT, MR, X-ray, ultrasound, PET, and so on); (2) segmentation of volumes of interest (VOI), which can be done manually by radiologists or automatically or semi-automatically by software; (3) feature extraction, extracting high-throughput features from VOIs, including shape features, first-order statistical features, texture features, and higher-order statistical features; (4) feature selection, excluding the non-repeatable, redundant, and irrelevant features from a large number of extracted features; (5) model construction, constructing the prediction model based on machine learning methods for a specific clinical problem, and training it [12–14].

Recent studies have demonstrated that radiomic features extracted from lung CT images can predict EGFR mutation status [10, 15–18]. However, most studies focus on intratumoral lesions and give little attention to subtle changes in the peritumoral region. Recent cancer studies have shown that as cancer infiltrates and metastasizes, the lung parenchyma surrounding the tumor may also be affected, and changes in the microenvironment, such as tumor angiogenesis, lymphangiogenesis, microvascular and lymphatic infiltration can provide valuable clinical information, which may reflect the biological behavior of the tumor, thus helping the characterization of tumor aggressiveness and the predicted prognosis of tumors [19, 20]. Therefore, mining peritumoral radiomic features may identify new biological markers for the non-invasive prediction of EGFR mutation in lung adenocarcinoma. We hope to develop a radiomics model combining intratumoral and peritumoral features to predict EGFR mutation status in lung adenocarcinoma patients non-invasively. We will explore the optimal peritumoral range corresponding to the highest AUC of the prediction model, which may be helpful for targeted therapy of lung adenocarcinoma.

## Materials and methods

This retrospective study was approved by The Second Xiangya Hospital, Institutional Review Board (No. 2022K012), which waived the requirement for patients’ informed consent referring to the Council for International Organizations of Medical Sciences (CIOMS) guidelines.

### Patients

**Fig. 1:**
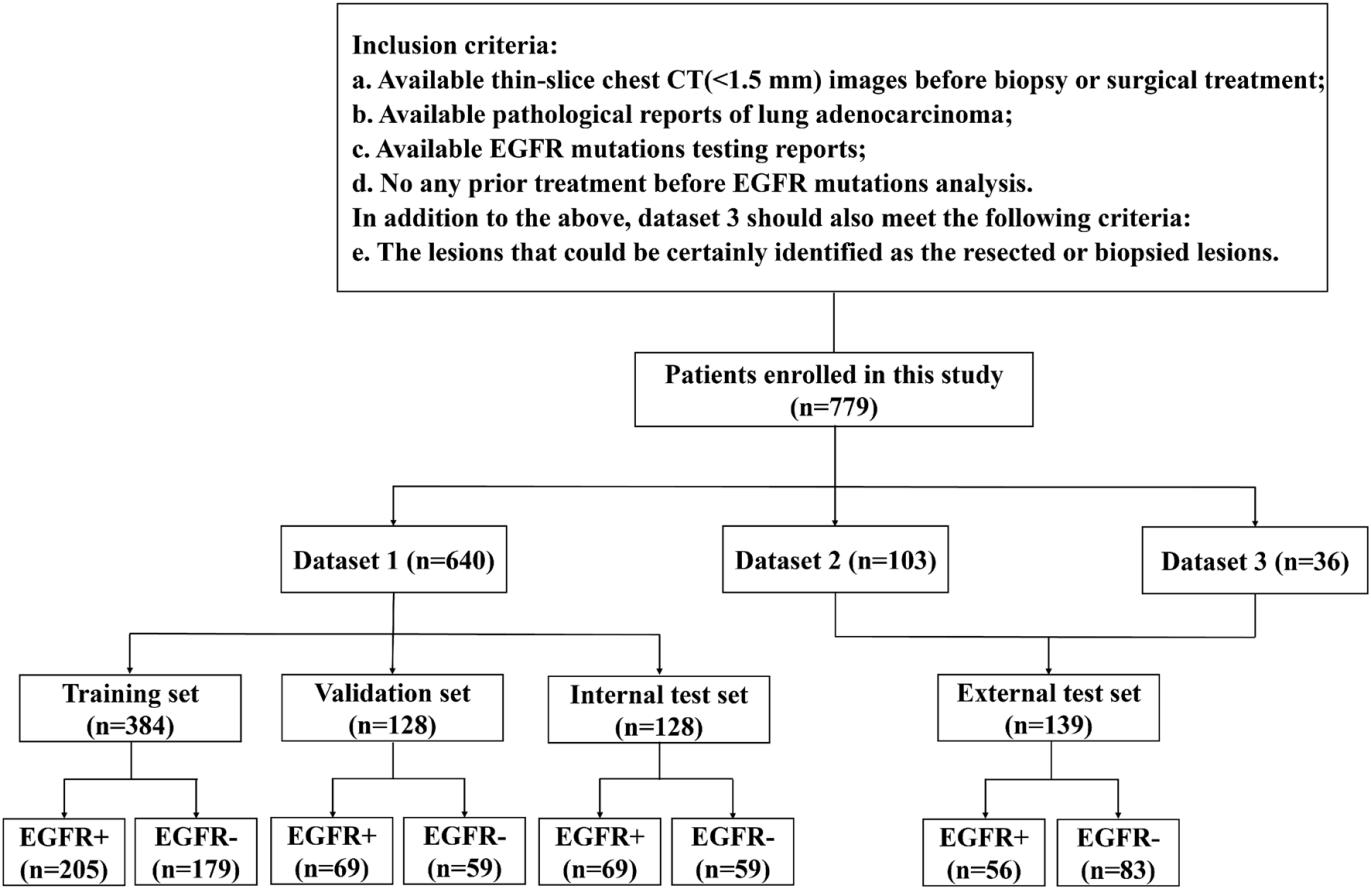
Patients’ inclusion flowchart and datasets partition. EGFR+— EGFR mutant; EGFR-—EGFR wild-type

We finally collected three datasets for analysis. Figure 1 shows the patients’ inclusion flowchart and datasets partition. Dataset 1 and dataset 2 were collected from two hospitals with the following inclusion criteria: (1) available non-contrast enhanced thin-slice chest CT (0.75–1.5 mm) scan before biopsy or surgical treatment; (2) available pathological reports of lung adenocarcinoma; (3) available EGFR mutation testing reports; and (4) no any prior treatment before EGFR mutation analysis. Dataset 3 was collected from the Cancer Imaging Archive (TCIA) public database with the following inclusion criteria: (1) available non-contrast enhanced CT images with slice thickness ≤ 1.5 mm (to avoid data inconsistency); (2) available pathological reports of lung adenocarcinoma; (3) available EGFR mutations testing reports; and (4) the lesions that could be certainly identified as the resected or biopsied lesions. Patients with CT images slice thickness > 1.5 mm, pathologically confirmed non-lung adenocarcinoma, and without EGFR mutations testing reports were excluded. CT acquisition and scanning parameters for dataset 1 and dataset 2 were presented in Supplementary Material 1.

A total of 779 patients were included in this study which were divided into EGFR + or EGFR– groups. Dataset 1, including 640 patients collected from the Huadong Hospital from January 2013 to December 2018, was randomly divided into a training set (384 patients, 60.0%), a validation set (128 patients, 20.0%), and an internal testing set (128 patients, 20.0%). Dataset 2, including 103 patients collected from the Second Xiangya Hospital from January 2020 to March 2021. Dataset 3, including 36 patients from TCIA. Dataset 2 and Dataset 3 were combined as an independent external testing set.

### Tumor segmentation and radiomic feature extraction

**Fig. 2:**
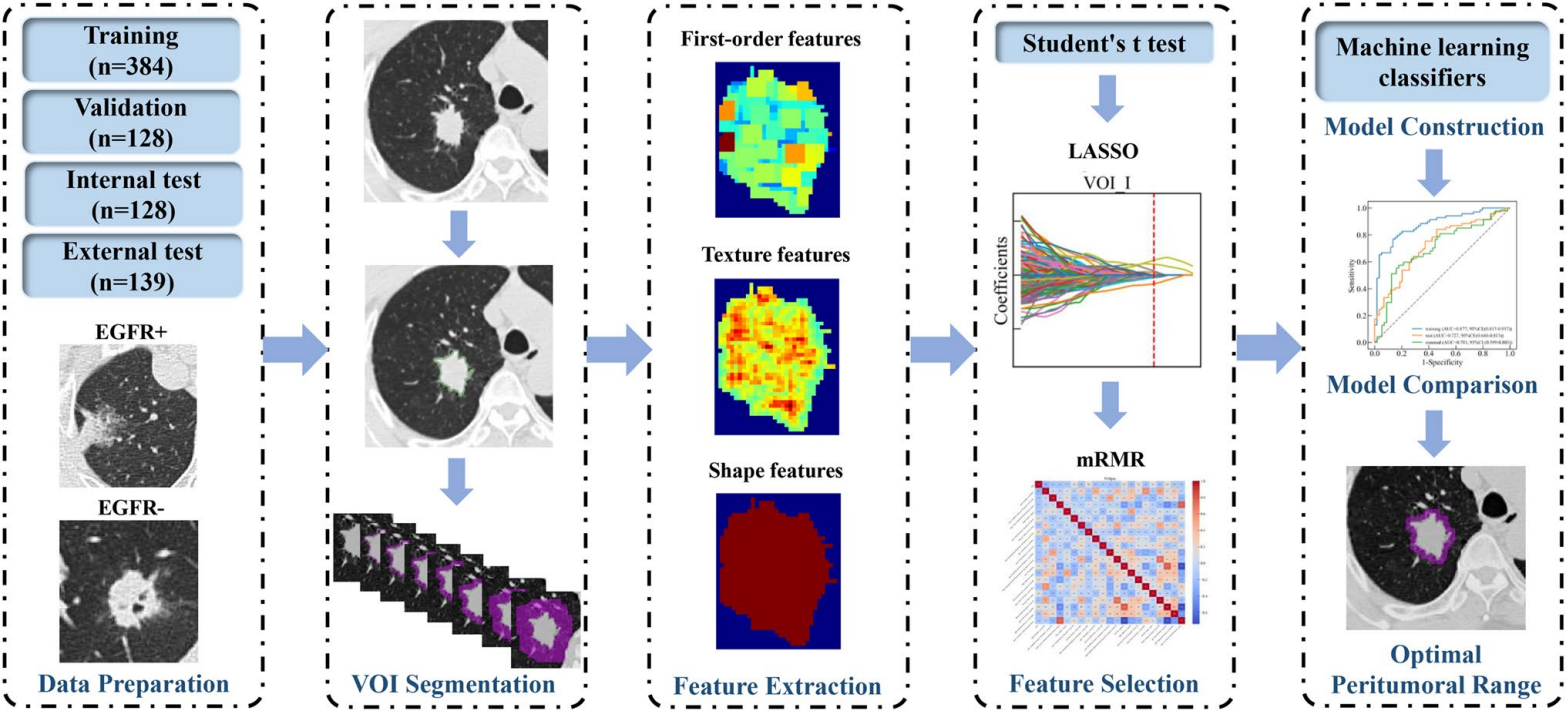
The workflow of the study. VOI—volume of interest; EGFR—Epidermal growth factor receptor; EGFR+—EGFR mutant; EGFR−—EGFR wild-type; LASSO—the least absolute shrinkage and selection operator; mRMR—the minimum redundancy maximum relevance algorithm

Firstly, intratumoral VOIs (VOI_I) were delineated manually along the lesion on every slice until the entire lesion was covered by a radiologist with 5-year experience in chest radiology and then confirmed or modified by a radiologist with 10-year experience in chest radiology using 3Dslicer software (version 4.10.1, Brigham and Women’s Hospital). In patients with multiple lesions, only one lesion was delineated due to the limited availability of EGFR testing reports. Secondly, to augment the spatial dimensions of tumor regions in our dataset, we employed a dilation technique facilitated by the “SimpleITK” library in Python to automatically expand VOI_I by 1 mm, 2 mm, 3 mm, 4 mm, 5 mm, 10 mm, and 15 mm. In essence, this approach involves enlarging the tumor mask by a specified distance in millimeters. The tumor region was represented as a binary mask, where the tumor cells were marked as 1 and the background regions were denoted as 0. The dilation of the tumor mask was then achieved using a spherical structuring element, corresponding to the desired extension distance. These peritumoral regions included air in the lungs, pulmonary vessels, and bronchi and did not include the chest wall and mediastinum. Figure 2 shows the process of tumor segmentation and its expansion into the peritumoral region. Finally, three kinds of regions were created: (1) intratumoral regions only (VOI_I); (2) peritumoral regions only (VOI_P), VOI_P1, VOI_P2, VOI_P3, VOI_P4, VOI_P5, VOI_P10, and VOI_P15; (3) intratumoral and peritumoral regions (combined), VOI1, VOI2, VOI3, VOI4, VOI5, VOI10, and VOI15. Images with VOI information were exported with NII format for the next step of analysis.

The original images were resampled at the same voxel size of 1*1*1 mm^3^ by cubic interpolation to achieve spatial resolution. Hounsfield Units (HU) were standardized by setting consistent window levels across all images, typically ranging from -1000 HU (air) to 1000 HU (bone). Bias in intensity non-uniformities was corrected to account for variations in scanner characteristics. Then, the Wavelet filter, Laplacian of Gaussian filter, Square filter, SquareRoot filter, Logarithm filter, and Exponential filter were used to pre-process the original images.

International Biomarker Standardization Initiative compliant radiomic features were extracted from these VOIs using Pyradiomics package (version 3.0.1) in Python. From original images and filtered images, a total of 1454 radiomic features were extracted from each VOI, including 288 first-order features, 14 shape features, and 1152 texture features. Texture features included Gray Level Co-occurrence Matrix (GLCM), Gray Level Size Zone Matrix (GLSZM), Gray Level Run Length Matrix (GLRLM), Neighboring Gray Tone Difference Matrix (NGTDM), and Gray Level Dependence Matrix (GLDM) features. The details of these features were presented in Supplementary Table S6.

### Feature selection and model construction

A three-step method was used to select radiomic features. First, the student’s *t*-test initially selected significantly different features between the EGFR + and EGFR- groups (*p* < 0.05). Next, the features with *p* < 0.05 were further selected by the least absolute shrinkage and selection operator (LASSO), tenfold cross-validation was applied to determine the optimal tuning parameter *λ* value, and then features with nonzero coefficients were selected. After removing the irrelevant or redundant features, we used the minimum redundancy maximum relevance (mRMR) algorithm to identify the most important features based on a heuristic scoring criterion and retained only the top-ranked features.

The optimal selected features were used to construct three kinds of radiomics models: (1) VOI_I model, a model with intratumoral radiomics alone; (2) VOI_P model, a model with peritumoral radiomics alone; (3) combined model, a model combining intratumoral and peritumoral radiomics. Multiple machine learning classifier algorithms, including Random Forest (RF), K-nearest neighbors (KNN), Logistic Regression (LR), Extremely Randomized Trees (ExtraTrees), CatBoost, eXtreme Gradient Boosting (XGBoost), NeuralNetFastAI, NeuralNetTorch, and Light Gradient Boosting Machine (LightGBM) were analyzed to determine the optimal classifier algorithm. Descriptions of these classifier algorithms and the optimal classifier algorithm corresponding to each VOI were shown in Supplementary Material 2 and Supplementary Table S1. For each VOI, the respective optimal classifier algorithm was selected to construct the radiomics models, respectively. The predictive performance of each model was evaluated using the area under the receiver operating characteristic curve (AUC), accuracy, sensitivity, specificity, and F1 score.

### Statistical analysis and model evaluation

The mean and standard deviations were expressed for continuous variables and frequency (percentage) for categorical variables. ANOVA and the chi-square test (or Fisher’s exact test) were used to assess statistical differences in continuous and categorical variables across three datasets, respectively. Statistical analyses were performed using the SPSS 27.0 software (IBM Corp, Armonk, USA). The predictive performance of the models was evaluated using AUC, accuracy, sensitivity, specificity, and F1 score. The DeLong test was used to assess the differences in AUC between different models. *p* < 0.05 indicated a significant difference.

## Results

### Clinical characteristics of patients

The clinical characteristics of patients are shown in Table 1. A total of 779 patients (345 males, 434 females) were included in this study. 399 patients (51.2%) were classified as EGFR mutant (EGFR+), while 380 (48.8%) were classified as wild-type (EGFR−). There were significant differences in smoking status, tumor subtype, and EGFR mutation status among patients in the three datasets.

**Table 1:** Clinical characteristics of patients

Variable	Dataset 1 (*n* = 640)	Dataset 2 (*n* = 103)	Dataset 3 (*n* = 36)	*p* value
Age (years)	59.8 ± 12.0	60.1 ± 12.4	66.8 ± 11.5	0.196
Gender				0.090
Male	272 (42.5%)	53 (51.5%)	20 (55.6%)	
Female	368 (57.5%)	50 (48.5%)	16 (44.4%)	
Smoking status				< 0.001
Current or former	49 (7.6%)	26 (25.2%)	26 (72.2%)	
Never	588 (91.9%)	52 (50.5%)	10 (27.8%)	
Missing	3 (0.5%)	25 (24.3%)	–	
Location				0.383
Right lobe	379 (59.2%)	58 (56.3%)	25 (69.4%)	
Left lobe	261 (40.8%)	45 (43.7%)	11 (30.6%)	
Tumor subtype				0.006
^#^GGN	143 (22.3%)	8 (7.8%)	3 (8.3%)	
Part solid	245 (38.3%)	13 (12.6%)	10 (27.8%)	
Solid	192 (30.0%)	16 (15.5%)	22 (61.1%)	
Missing	60 (9.4%)	66 (64.1%)	1 (2.8%)	
*EGFR mutation status				0.002
+	343 (53.6%)	47 (45.6%)	9 (25.0%)	
−	297 (46.4%)	56 (54.4%)	27 (75.0%)	
*EGFR mutation subtype				–
19 Del	120 (35.0%)	–	–	
L858R	185 (54.0%)	–	–	
Others	38 (11.0%)	–	–	

*EGFR—Epidermal growth factor receptor

^#^GGN—ground-glass nodule

### Feature selection

**Fig. 3:**
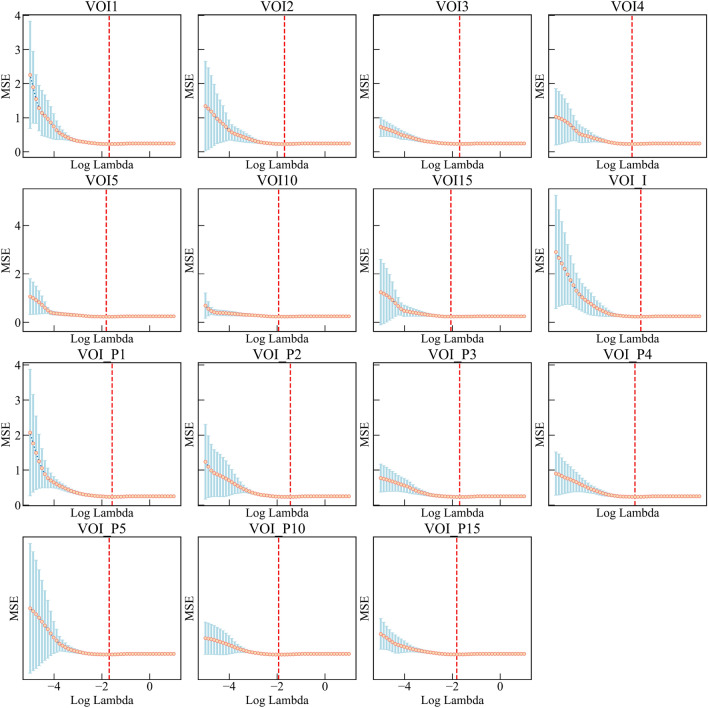
Feature selection based on the least absolute shrinkage and selection operator (LASSO) method. Identification of the optimal parameter *λ* in the LASSO model using tenfold cross-validation, drawing vertical lines at the optimal values via minimum criteria

**Fig. 4:**
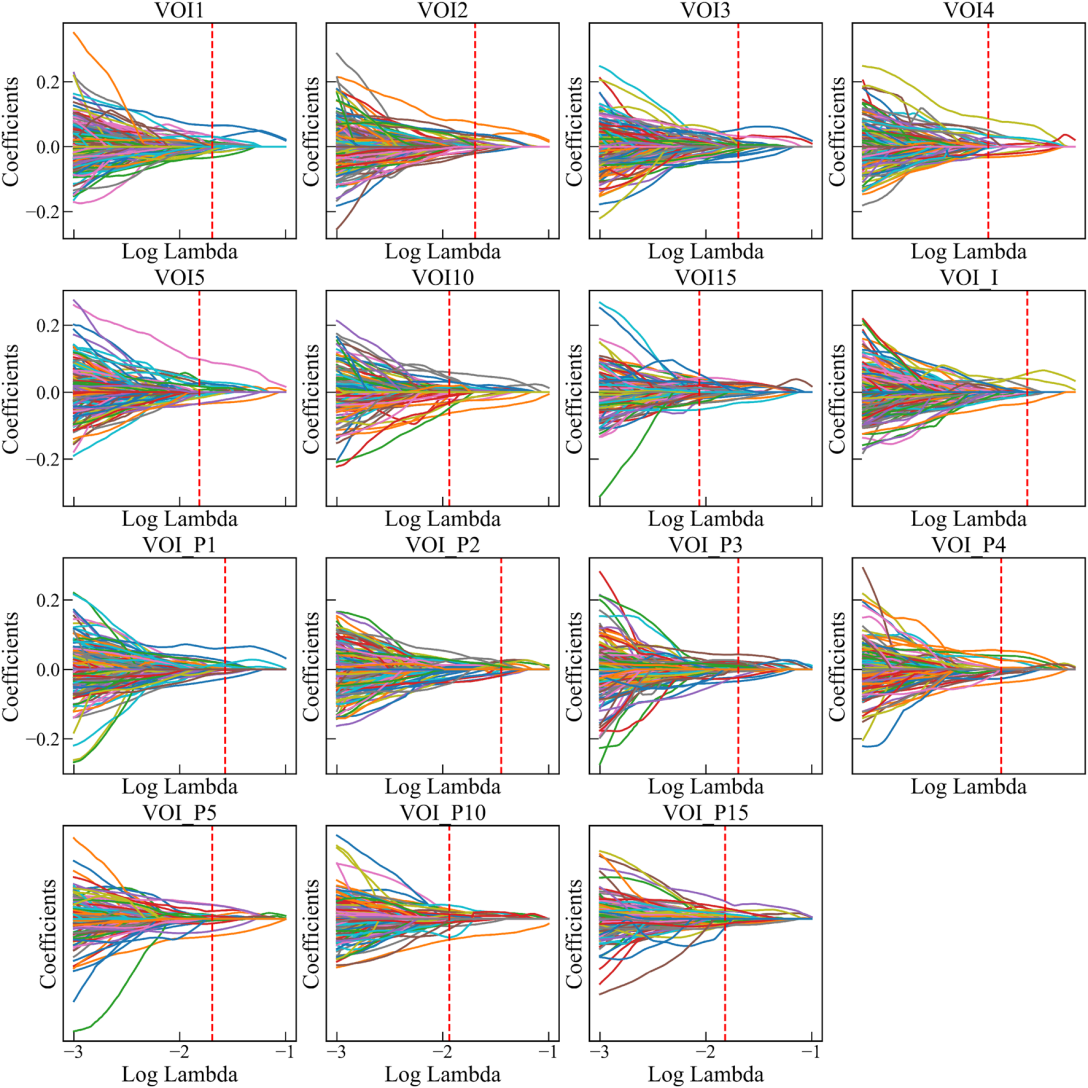
LASSO coefficient distributions of radiomic features for each VOI. Drawing vertical lines at the values selected using tenfold cross-validation, and features with nonzero coefficients in the LASSO regression model were the most predictive features

**Fig. 5:**
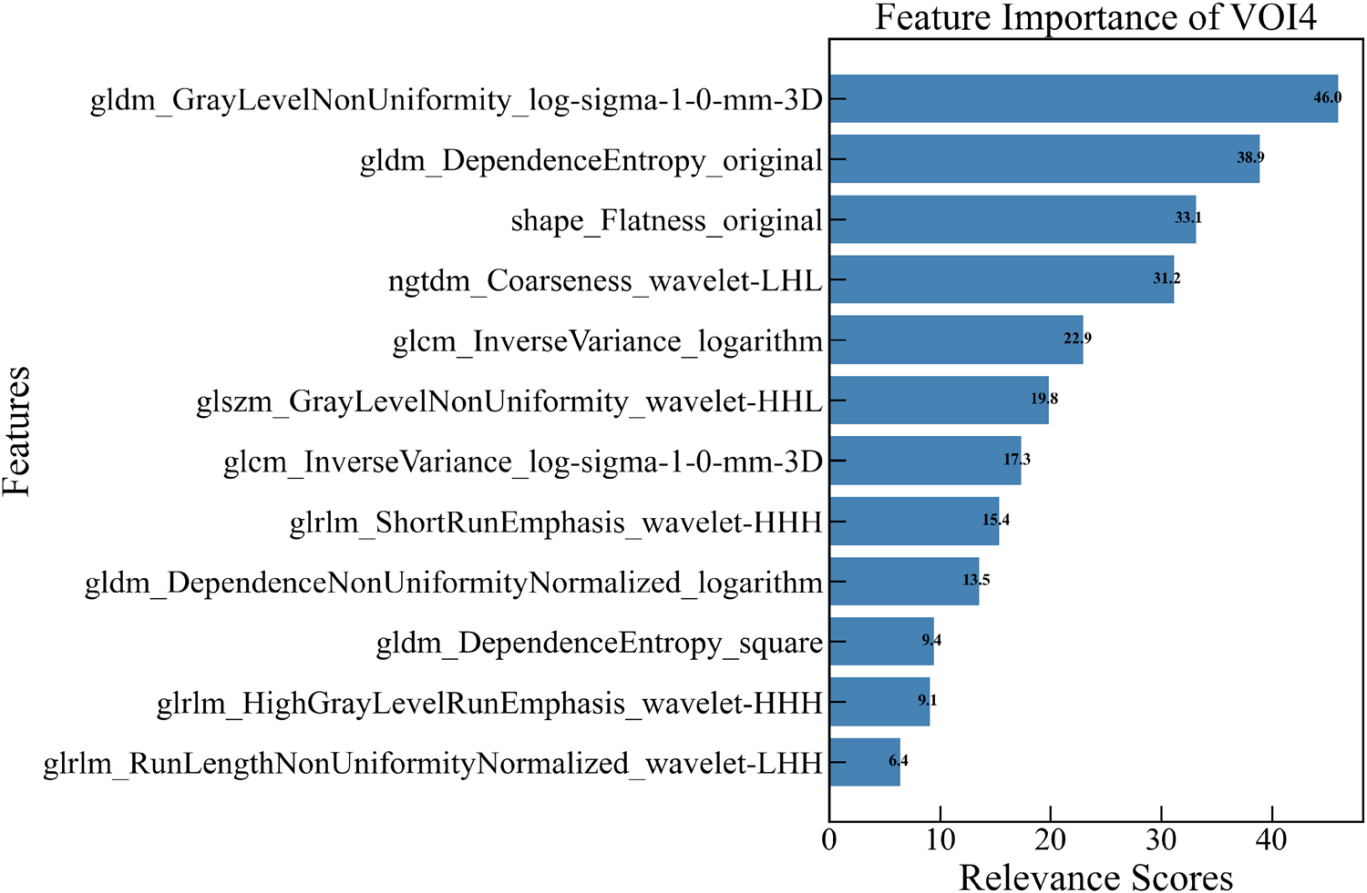
Radiomic features selected for VOI4 and their importance

After performing t-test, LASSO (Figs. 3, 4), and mRMR (Supplementary Fig. S1), a total of 262 highly predictive radiomic features were selected from 15 VOIs, including 10 first-order features, 17 shape features, and 235 texture features. The details of finally selected features and their importance for each VOI are presented in Table 2 and Supplementary Fig. S2, and features selected for VOI4 and their importance are presented in Fig. 5. Noting that each selected feature group of 15 VOIs included texture features and one shape feature (shape_Flatness feature), while only VOI_P1, VOI_P2, VOI_I, VOI1, VOI10, and VOI15 included first-order features.

**Table 2:** The details of finally selected features for each VOI

*VOI	Number of features			
	First-order features	^#^Shape features	Texture features	Total
VOI_P1	2	1	17	20
VOI_P2	1	1	18	20
VOI_P3	0	1	19	20
VOI_P4	0	1	19	20
VOI_P5	0	1	19	20
VOI_P10	0	1	19	20
VOI_P15	0	2	18	20
VOI_I	2	1	8	11
VOI1	2	1	12	15
VOI2	0	1	9	10
VOI3	0	1	19	20
VOI4	0	1	11	12
VOI5	0	1	13	14
VOI10	2	1	17	20
VOI15	1	2	17	20
Total	10	17	235	262

*VOI—volume of interest

^#^The final selected shape features for each VOI had “shape_Flatness feature”

### Predictive performance of VOI_I model and combined models

**Fig. 6:**
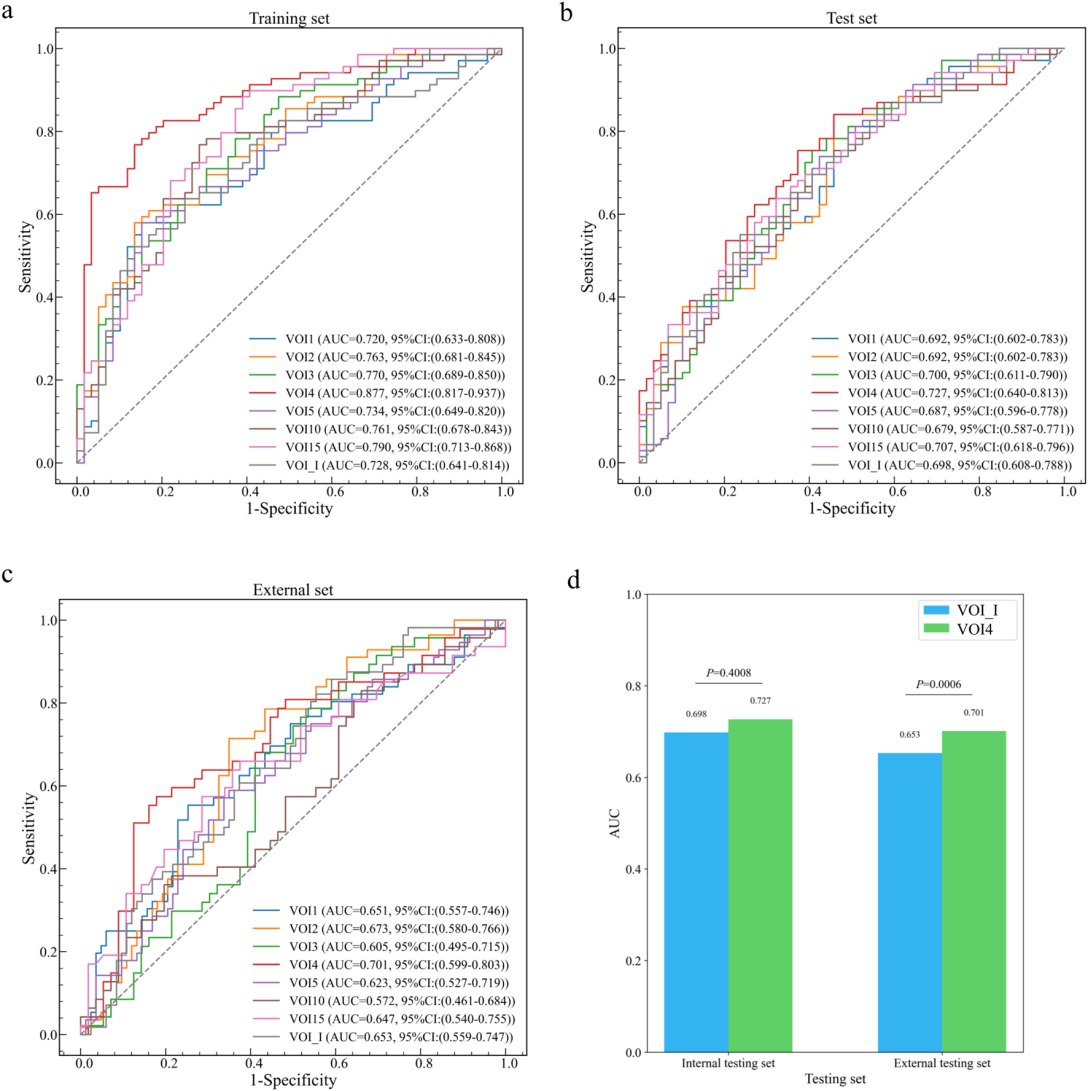
ROC curves of VOI_I model and combined models in the training and validation sets (**a**), internal (**b**) and external (**c**) testing sets, and the difference in AUC between VOI_I model and VOI4 model in the internal and external testing sets, respectively. Blue represents VOI_I model and green represents VOI4 model. VOI—volume of interest; AUC—area under the curve; ROC—Receiver operating characteristic; CI—confidence interval

In the training and validation sets, the VOI_I model performed well with an AUC of 0.728, and the AUCs of VOI2, VOI3, VOI4, VOI5, VOI10, and VOI15 models were higher than that of VOI_I model, which were 0.763, 0.770, 0.877, 0.734, 0.761, 0.790, respectively, with VOI4 model having the highest AUC, accuracy, sensitivity, and F1 score (Table 3, Fig. 6a).

**Table 3:** Predictive performance of VOI_I model and combined models

*VOI	^#^AUC	Accuracy	Sensitivity	Specificity	F1
*Training and validation sets*					
VOI_I	0.728	68.0%	71.0%	64.4%	70.5%
VOI1	0.720	64.1%	62.3%	66.1%	65.2%
VOI2	0.763	68.0%	71.0%	64.4%	70.5%
VOI3	0.770	68.8%	72.5%	64.4%	71.4%
VOI4	0.877	81.3%	82.6%	79.7%	82.6%
VOI5	0.734	65.6%	68.1%	62.7%	68.1%
VOI10	0.761	71.9%	72.5%	71.2%	73.5%
VOI15	0.790	71.9%	72.5%	71.2%	73.5%
*Internal testing set*					
VOI_I	0.698	64.1%	73.9%	52.5%	68.9%
VOI1	0.692	60.9%	66.7%	54.2%	64.8%
VOI2	0.692	62.5%	68.1%	55.9%	66.2%
VOI3	0.700	66.4%	78.3%	52.5%	71.5%
VOI4	0.727	68.0%	79.7%	54.2%	72.8%
VOI5	0.687	64.8%	76.8%	50.8%	70.2%
VOI10	0.679	63.3%	71.0%	54.2%	67.6%
VOI15	0.707	64.1%	71.0%	55.9%	68.1%
*External testing set*					
VOI_I	0.653	59.0%	78.6%	45.8%	60.7%
VOI1	0.651	50.4%	82.1%	28.9%	57.1%
VOI2	0.673	59.0%	80.4%	44.6%	61.2%
VOI3	0.605	58.3%	78.7%	41.1%	63.2%
VOI4	0.701	62.1%	66.0%	58.9%	61.4%
VOI5	0.623	57.6%	75.0%	45.8%	58.7%
VOI10	0.572	50.5%	57.4%	44.6%	51.4%
VOI15	0.647	56.3%	74.5%	41.1%	60.9%

^*^VOI—volume of interest

^#^AUC—area under the curve

In the internal testing set, the AUCs of VOI3, VOI4, and VOI15 models were higher than that of VOI_I model (AUC = 0.698), which were 0.700, 0.727, and 0.707, respectively, with VOI4 model having the highest AUC, accuracy, sensitivity, and F1 score (Table 3, Fig. 6b).

In the external testing set, the AUCs of VOI2, and VOI4 models were higher than that of VOI_I model (AUC = 0.653), which were 0.673, and 0.701, respectively, with VOI4 model having the highest AUC, accuracy, and specificity (Table 3, Fig. 6c).

In addition, we used the DeLong test to evaluate the difference in AUC between models in the internal and external testing sets, respectively (Supplementary Table S2, Supplementary Table S3). For VOI4 model, the AUC was significantly different from that of VOI_I in the external test set (*p* = 0.0006) (Fig. 6d).

### Predictive performance of VOI_P models

**Fig. 7:**
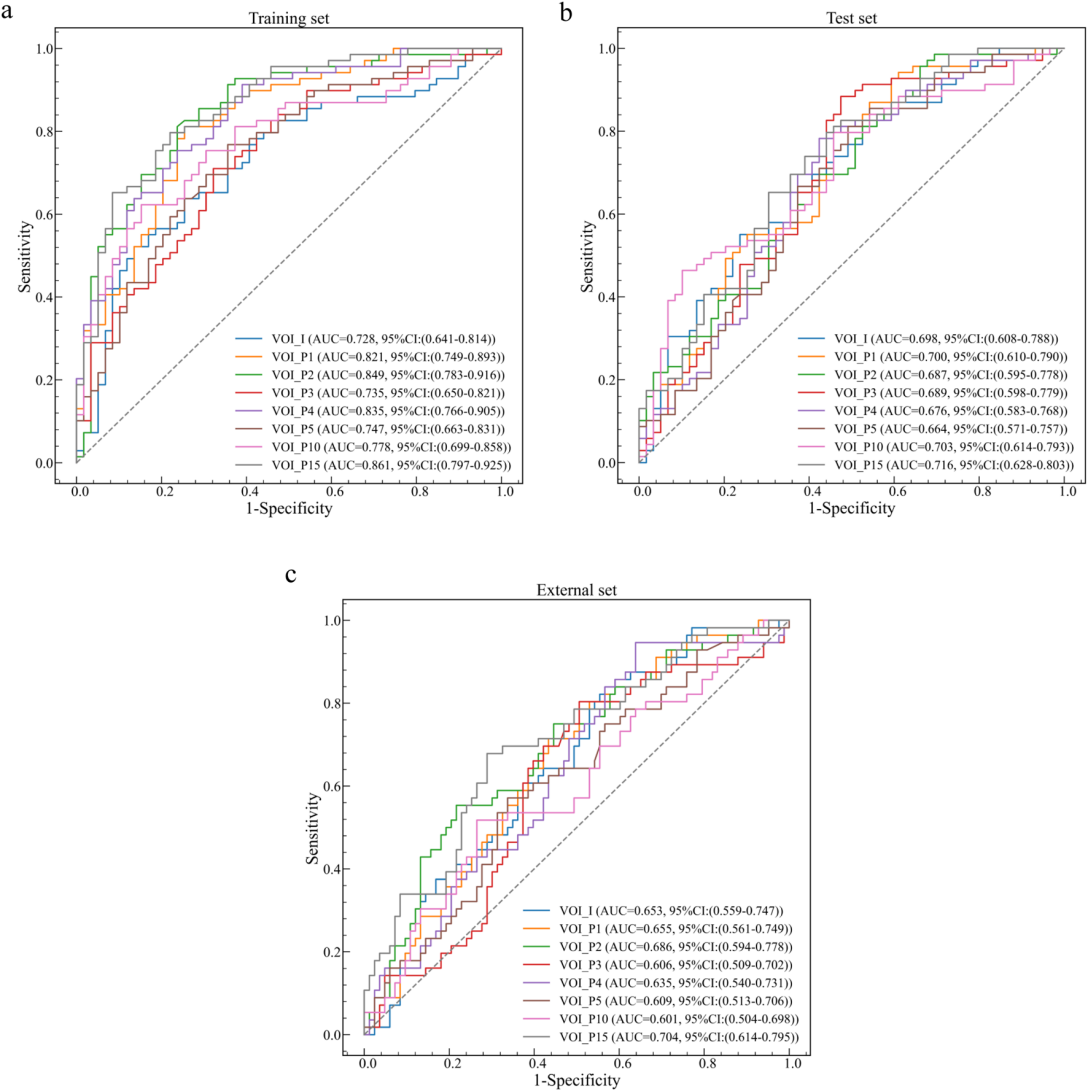
ROC curves of VOI_P models in the training and validation sets (**a**), internal (**b**) and external (**c**) testing sets. ROC—Receiver operating characteristic; VOI—volume of interest; AUC—area under the curve; CI—confidence interval

Compared to other VOI_P models, the model based on the peritumoral 15 mm (VOI_P15) features alone achieved the best performance in the training and validation sets, internal testing set, and external testing set, with AUCs of 0.861, 0.716, and 0.704, respectively (Table 4, Fig. 7). The results of DeLong test are presented in Supplementary Table S4 and Supplementary Table S5.

**Table 4:** Predictive performance of VOI_P models

*VOI	^#^AUC	Accuracy	Sensitivity	Specificity	F1
*Training and validation sets*					
VOI_P1	0.821	77.3%	78.3%	76.3%	78.8%
VOI_P2	0.849	78.9%	82.6%	74.6%	80.9%
VOI_P3	0.735	59.4%	27.5%	96.6%	42.2%
VOI_P4	0.835	75.0%	81.2%	67.8%	77.8%
VOI_P5	0.747	57.0%	27.5%	91.5%	40.9%
VOI_P10	0.778	68.8%	62.3%	76.3%	68.3%
VOI_P15	0.861	76.6%	81.2%	71.2%	78.9%
*Internal testing set*					
VOI_P1	0.700	67.2%	79.7%	52.5%	72.4%
VOI_P2	0.687	61.7%	72.5%	49.2%	67.1%
VOI_P3	0.689	52.3%	21.7%	88.1%	33.0%
VOI_P4	0.676	68.0%	79.7%	54.2%	72.8%
VOI_P5	0.664	50.0%	17.4%	88.1%	27.3%
VOI_P10	0.703	60.9%	55.1%	67.8%	60.3%
VOI_P15	0.716	66.4%	75.4%	55.9%	70.7%
*External testing set*					
VOI_P1	0.655	59.0%	73.2%	49.4%	59.0%
VOI_P2	0.686	56.8%	78.6%	42.2%	59.5%
VOI_P3	0.606	59.7%	7.1%	95.2%	12.5%
VOI_P4	0.635	59.0%	75.0%	48.2%	59.6%
VOI_P5	0.609	59.0%	1.8%	97.6%	3.4%
VOI_P10	0.601	63.3%	48.2%	73.5%	51.4%
VOI_P15	0.704	61.9%	78.6%	50.6%	62.4%

^*^VOI—volume of interest

^#^AUC—area under the curve

## Discussion

In this study, we constructed three kinds of radiomics models: (1) intratumoral model (VOI_I model); (2) peritumoral model (VOI_P model); (3) intratumoral and peritumoral model (combined model). We found that combined models showed great promise in predicting the EGFR mutation status of lung adenocarcinoma patients. The best prediction performance was obtained by VOI4 model, with the highest AUCs of 0.877, 0.727, and 0.701 in the training and validation sets, the internal testing set, and the external testing set, respectively.

To our knowledge, few studies have revealed the added value of peritumoral radiomics in predicting EGFR mutation status in lung cancer. Choe et al. demonstrated that the predictive model combining intratumoral and peritumoral radiomic features performed slightly better in the training set than the intratumoral model, but the difference was not statistically significant (AUC = 0.66 vs. 0.64, *p* = 0.504), whereas, in the validation set, the AUC was lower than that of the intratumoral model (AUC = 0.56 vs. 0.62) [21]. Another study showed that compared to intratumoral radiomics alone, combining intratumoral and peritumoral 3 mm radiomic features significantly improved the predictive performance of EGFR mutation status in primary lung cancer (AUC = 0.730 vs. 0.774, *p* < 0.001), and in lung adenocarcinoma only (AUC = 0.687 vs. 0.630, *p* < 0.001) [22]. However, this study did not determine whether the 3 mm peritumoral region was optimal for evaluating peritumoral features. Ideally, to determine the best peritumoral range, we should extract features from different peritumoral ranges to construct models separately and compare their predictive performance. A recent study compared radiomic features of multiple peritumoral regions (3 mm, 5 mm, 7 mm) and constructed three machine learning models to predict EGFR mutation status in NSCLC. The results showed that combining intratumoral and peritumoral 3 mm radiomic features could better distinguish EGFR+ from EGFR− groups than 5 mm and 7 mm (training, *p* = 0.0000, test, *p* = 0.0025), but this study included only 164 patients and did not validate models with an external dataset [23]. Based on this, we expanded VOI_I outwards by 1 mm, 2 mm, 3 mm, 4 mm, 5 mm, 10 mm, and 15 mm to identify seven peritumoral regions and combined them with intratumoral regions to generate seven intratumoral and peritumoral regions, respectively, to compare the complementary value of different peritumoral regions to the predictive performance of radiomic models. In addition, compared to the previous studies, our study used a larger training cohort and was tested in an independent internal testing set and an external testing set. As a result, our model may be more effective in illustrating the differences in radiomic features between EGFR+ and EGFR− groups.

According to the results, the peritumoral region of lung adenocarcinoma may also provide important predictive information about EGFR mutations, with the best predictive performance achieved by combining intratumoral and peritumoral 4 mm radiomic features. Tumor cells are usually highly invasive and tend to migrate from the primary tumor to the surrounding parenchyma, disrupting the normal structure and causing morphological and textural changes in the peritumoral region. These changes are difficult to detect on medical images, whereas radiomic features extracted from CT images can quantitatively reflect subtle changes in the microenvironment surrounding the tumor that cannot be recognized by the naked eye, this may be the pathophysiological basis for the improved predictive performance of the combined models over the VOI_I model. Lung adenocarcinomas have obvious cellular and mutational heterogeneity. The concept of tumor heterogeneity applies not only to tumor epithelial cells but also to the various microenvironments with which the tumor cells interact, such as vasculature, cancer-associated fibroblasts, extracellular matrix, and infiltrating immune cells. Tumor cells can influence their microenvironment by releasing cell signaling molecules that promote tumor angiogenesis and induce immunological tolerance. Meanwhile, immunocytes infiltrated in the tumor microenvironment can secrete a large number of cytokines and chemokines to promote the epithelial-mesenchymal transition of tumor cells, which allows tumor cells to invade and metastasis [24].

The tumor margin is an important meeting place in the tumor microenvironment where immune and stromal cells are highly active and interact with the tumor. The microenvironment at tumor invasion edges differs from that of the tumor core. Hypoxia tends to be associated with the center of the tumor, whereas oxygen is primarily present at the tumor periphery. Monocytes in the blood are recruited around tumor cells by various chemokines and cytokines, thus becoming tumor-associated macrophages, which can promote the invasion of tumor cells by supplying pro-migratory factors such as epidermal growth factor, or by promoting extracellular matrix proteolytic remodeling, and play an important role in the invasion process of the tumor margin. Furthermore, under hypoxic conditions, tumor-associated macrophages promote tumor cell release of vascular endothelial growth factor and platelet-derived growth factor via the activation of the hypoxia-inducible factor-1 pathway, thus promoting tumor angiogenesis, providing oxygen and nutrients for tumor growth, and contributing to tumor cell invasion and metastasis. In addition, tumor-associated fibroblasts are also abundant at the tumor margin, promoting tumor proliferation, angiogenesis, invasion, and metastasis by secreting various growth factors, cytokines, and inflammatory chemokines [25, 26].

As in several previous studies, the most predictive radiomic features finally selected in our study included a significant number of texture features (235 in total), which reflect the pattern and spatial distribution of voxel intensities within the VOI, indicating its biological heterogeneity [15]. Therefore, our results may suggest that tumor heterogeneity is associated with EGFR mutation status in lung adenocarcinoma. Regarding the shape features, the shape_Flatness feature was found in all of the final selected features of 15 VOIs, which shows the relationship between the largest and smallest principal components in the VOI shape, suggesting that this feature plays an important role in predicting EGFR mutation status. However, unlike most other studies [16, 22, 27, 28] there were no first-order features in our best predictive model (VOI4). The first-order features describe the distribution of voxel intensities within the target region through commonly used and basic metrics, but it is difficult to measure the spatial distribution characteristics of voxels without considering the neighborhood relationship between voxels [29]. In our best predictive model, they are not critical predictive features.

In addition, we found that features from independent peritumoral regions also had predictive value for the prediction of EGFR mutations. Compared to other peritumoral radiomics models, the model based on the peritumoral 15 mm (VOI_P15) features achieved the best performance in the training and validation sets, the internal testing set, and the external testing set, with AUCs of 0.861, 0.716, and 0.704, respectively. However, this was inconsistent with findings that as peritumoral distance increased, the VOI comprised more normal lung tissue and relatively less tumor tissue, making the predictive performance of the model decreased [30]. The probable explanation was that radiomic features were more stable as peritumoral distance increased [31]. Tunali et al. also demonstrated that some radiomic features, including statistical features, histograms, and some texture features (GLCM, GLRLM, GLSZM, and NGTDM), had good stability and reproducibility regardless of peritumoral distance, indicating that these features were less influenced by changes in the size or shape of peritumoral regions caused by different segmentation and image acquisition [31]. It was generally consistent with the features eventually selected in our study, and these stable and reproducible features were more likely to construct robust radiomics models, allowing multicenter studies to maximize the clinical utility of radiomics models [32].

To achieve more generalizable and impactful results in radiomics, researchers need to obtain large patient cohorts by combining images from multiple institutions. However, most current radiomics studies collect imaging data retrospectively, and image acquisition protocols, processing or reconstruction settings, and imaging scanners may be different from different institutions, resulting in poor reproducibility and repeatability of radiomic features [33–35]. Therefore, in order to discover more reliable and stable radiomic features and apply them in multicenter clinical practice, image consistency must be improved by controlling imaging protocols in order to build a public database with a large amount of high-quality data [36]. In addition, several studies have demonstrated that the use of harmonization methods in the image domain (prior to feature extraction) or spatial domain (within or after feature extraction) would be beneficial in the design of multicenter studies. According to recent studies, ComBat harmonization is a fast and easy-to-use feature harmonization method in the feature domain that allows the correction of radiomic features to reduce the variation caused by different imaging protocols [37–39]. It was first proposed by Johnsond et al. [40] for genetic studies and was later used by Fortin et al. for medical imaging applications [41], and by Orlhac et al. [42] for PET radiomics studies, and had produced great results in several subsequent studies [39, 43, 44]. Among them, Shiri et al. demonstrated that ComBat harmonization could significantly improve the prediction performance when radiomics to predict EGFR mutation status in NSCLC, and the range of mean AUC increased from 0.87–0.90 to 0.92–0.94, which proved the effectiveness of ComBat harmonization [43]. Therefore, we can try to apply ComBat harmonization to further improve the prediction performance of the model in future.

Despite the encouraging results, there are still some limitations. First, we included some lung adenocarcinoma patients as an external testing set to validate the reliability and stability of the model, however, due to the small sample size, its predictive efficiency may be limited, and multi-institutional image data are needed to assess the generalizability of our findings in future; second, the incidence of EGFR mutation varies greatly across different races, with a significantly higher incidence in Asian populations [45]. The patients used for model training in our study were all Asians, making the results lacking in generalizability and requiring further validation in patients of other races; furthermore, some other potentially valuable factors such as smoking status and gender were not included in this study, and we will combine radiomic features with these clinical features for further research to improve the predictive performance of the model in future.

In conclusion, radiomic features extracted from the peritumoral region can add extra value in predicting the EGFR mutation status of lung adenocarcinoma patients, with the optimal peritumoral range of 4 mm. This may partially prove the clinical value of peritumoral microenvironment in cancer diagnosis.

### Supplementary Information

Below is the link to the electronic supplementary material.

Supplementary file1 (PDF 3163 kb)

## References

[1] Pao W, Girard N (2011). New driver mutations in non-small-cell lung cancer. Lancet Oncol.

[2] Remon J, Steuer CE, Ramalingam SS, Felip E (2018). Osimertinib and other third-generation EGFR TKI in EGFR-mutant NSCLC patients. Ann Oncol.

[3] Moore S, Wheatley-Price P (2021). EGFR combination therapy should become the new standard first-line treatment in advanced EGFR-mutant NSCLC. J Thorac Oncol.

[4] Stock-Martineau S, Shepherd FA (2021). EGFR tyrosine kinase inhibitor monotherapy should remain the standard first-line treatment in advanced EGFR-mutant NSCLC. J Thorac Oncol.

[5] Tang W, Li X, Xie X (2019). EGFR inhibitors as adjuvant therapy for resected non-small cell lung cancer harboring EGFR mutations. Lung Cancer.

[6] Wu S-G, Shih J-Y (2018). Management of acquired resistance to EGFR TKI-targeted therapy in advanced non-small cell lung cancer. Mol Cancer.

[7] Ettinger DS, Wood DE, Aisner DL (2022). Non-small cell lung cancer, version 3.2022, NCCN clinical practice guidelines in oncology. J Natl Compr Canc Netw.

[8] Rossi G, Barabino E, Fedeli A (2021). Radiomic detection of EGFR mutations in NSCLC. Cancer Res.

[9] Wang C, Ma J, Shao J (2022). Predicting EGFR and PD-L1 status in NSCLC patients using multitask AI system based on CT images. Front Immunol.

[10] Cheng B, Deng H, Zhao Y (2022). Predicting EGFR mutation status in lung adenocarcinoma presenting as ground-glass opacity: utilizing radiomics model in clinical translation. Eur Radiol.

[11] Tan X, Li Y, Wang S (2022). Predicting EGFR mutation, ALK rearrangement, and uncommon EGFR mutation in NSCLC patients by driverless artificial intelligence: a cohort study. Resp Res.

[12] Scapicchio C, Gabelloni M, Barucci A (2021). A deep look into radiomics. Radiol med.

[13] Lambin P, Rios-Velazquez E, Leijenaar R (2012). Radiomics: extracting more information from medical images using advanced feature analysis. Eur J Cancer.

[14] Tang X, Li Y, Yan W-F (2021). Machine learning-based CT radiomics analysis for prognostic prediction in metastatic non-small cell lung cancer patients with EGFR-T790M mutation receiving third-generation EGFR-TKI osimertinib treatment. Front Oncol.

[15] Hou S, Fan Y, Wang X (2022). Radiomics for detection of the EGFR mutation in liver metastatic NSCLC. Acad Radiol.

[16] Lu X, Li M, Zhang H (2020). A novel radiomic nomogram for predicting epidermal growth factor receptor mutation in peripheral lung adenocarcinoma. Phys Med Biol.

[17] Wu S, Shen G, Mao J, Gao B (2020). CT radiomics in predicting EGFR mutation in non-small cell lung cancer: a single institutional study. Front Oncol.

[18] Chen W, Hua Y, Mao D (2021). A computed tomography-derived radiomics approach for predicting uncommon EGFR mutation in patients with NSCLC. Front Oncol.

[19] Wang S, Yu H, Gan Y (2022). Mining whole-lung information by artificial intelligence for predicting EGFR genotype and targeted therapy response in lung cancer: a multicohort study. Lancet Digit Health.

[20] Uthoff J, Stephens MJ, Newell JD (2019). Machine learning approach for distinguishing malignant and benign lung nodules utilizing standardized perinodular parenchymal features from CT. Med Phys.

[21] Choe J, Lee SM, Kim W (2021). CT radiomics-based prediction of anaplastic lymphoma kinase and epidermal growth factor receptor mutations in lung adenocarcinoma. Eur J Radiol.

[22] Yamazaki M, Yagi T, Tominaga M (2022). Role of intratumoral and peritumoral CT radiomics for the prediction of EGFR gene mutation in primary lung cancer. Br J Radiol.

[23] Kawazoe Y, Shiinoki T, Fujimoto K (2023). Investigation of the combination of intratumoral and peritumoral radiomic signatures for predicting epidermal growth factor receptor mutation in lung adenocarcinoma. J Appl Clin Med Phys.

[24] Chen Z, Fillmore CM, Hammerman PS (2014). Non-small-cell lung cancers: a heterogeneous set of diseases. Nat Rev Cancer.

[25] Quail DF, Joyce JA (2013). Microenvironmental regulation of tumor progression and metastasis. Nat Med.

[26] Christofides A, Strauss L, Yeo A (2022). The complex role of tumor-infiltrating macrophages. Nat Immunol.

[27] Zhang G, Deng L, Zhang J (2022). Development of a nomogram based on 3D CT radiomics signature to predict the mutation status of EGFR molecular subtypes in lung adenocarcinoma: a multicenter study. Front Oncol.

[28] Zhang X, Lu B, Yang X (2022). Prognostic analysis and risk stratification of lung adenocarcinoma undergoing EGFR-TKI therapy with time-serial CT-based radiomics signature. Eur Radiol.

[29] Bera K, Braman N, Gupta A (2022). Predicting cancer outcomes with radiomics and artificial intelligence in radiology. Nat Rev Clin Oncol.

[30] Wu L, Lou X, Kong N (2023). Can quantitative peritumoral CT radiomics features predict the prognosis of patients with non-small cell lung cancer? A systematic review. Eur Radiol.

[31] Tunali I, Hall LO, Napel S (2019). Stability and reproducibility of computed tomography radiomic features extracted from peritumoral regions of lung cancer lesions. Med Phys.

[32] Lambin P, Leijenaar RTH, Deist TM (2017). Radiomics: the bridge between medical imaging and personalized medicine. Nat Rev Clin Oncol.

[33] Emaminejad N, Wahi-Anwar MW, Kim GHJ (2021). Reproducibility of lung nodule radiomic features: multivariable and univariable investigations that account for interactions between CT acquisition and reconstruction parameters. Med Phys.

[34] Edalat-Javid M, Shiri I, Hajianfar G (2021). Cardiac SPECT radiomic features repeatability and reproducibility: a multi-scanner phantom study. J Nucl Cardiol.

[35] Zou K, Chen Z, Yuan X (2023). A review of uncertainty estimation and its application in medical imaging. Meta-Radiol.

[36] Zhao B (2021). Understanding sources of variation to improve the reproducibility of radiomics. Front Oncol.

[37] Zwanenburg A (2019). Radiomics in nuclear medicine: robustness, reproducibility, standardization, and how to avoid data analysis traps and replication crisis. Eur J Nucl Med Mol I.

[38] Da-Ano R, Visvikis D, Hatt M (2020). Harmonization strategies for multicenter radiomics investigations. Phys Med Biol.

[39] Orlhac F, Eertink JJ, Cottereau A-S (2022). A guide to ComBat harmonization of imaging biomarkers in multicenter studies. J Nucl Med.

[40] Johnson WE, Li C, Rabinovic A (2007). Adjusting batch effects in microarray expression data using empirical Bayes methods. Biostatistics.

[41] Fortin J-P, Cullen N, Sheline YI (2018). Harmonization of cortical thickness measurements across scanners and sites. Neuroimage.

[42] Orlhac F, Boughdad S, Philippe C (2018). A postreconstruction harmonization method for multicenter radiomic studies in PET. J Nucl Med.

[43] Shiri I, Amini M, Nazari M (2022). Impact of feature harmonization on radiogenomics analysis: prediction of EGFR and KRAS mutations from non-small cell lung cancer PET/CT images. Comput Biol Med.

[44] Mahon RN, Ghita M, Hugo GD, Weiss E (2020). ComBat harmonization for radiomic features in independent phantom and lung cancer patient computed tomography datasets. Phys Med Biol.

[45] Midha A, Dearden S, McCormack R (2015). EGFR mutation incidence in non-small-cell lung cancer of adenocarcinoma histology: a systematic review and global map by ethnicity (mutMapII). Am J Cancer Res.

