# Supplementary material for: Computed Tomography-derived intratumoral and peritumoral radiomics in predicting EGFR mutation in lung adenocarcinoma
Source: Radiol Med. 2023 Sep 25;128(12):1483–96. doi: 10.1007/s11547-023-01722-6 (PMC10700425; doi:10.1007/s11547-023-01722-6)
Supplement: Supplementary file 1 — Supplementary file1 (PDF 3163 kb) [file 11547_2023_1722_MOESM1_ESM.pdf]

## Supplementary Information

### Computed Tomography-derived intratumoral and peritumoral radiomics in predicting EGFR mutation in lung adenocarcinoma

Youlan Shang[1]*, Weidao Chen[2]*, Ge Li[3], Yijie Huang[1], Yisong Wang[1], Xiaoyan Kui[4], Ming Li[5], Hairong Zheng[6], Wei Zhao[1,6,7†], Jun Liu[1,7†]

1. Department of Radiology, The Second Xiangya Hospital, Central South University, No. 139 Middle Renmin Road, Changsha 410011, Hunan, P. R. China
2. 18 / F, Seat E, Ocean International Center, Infervision, Chaoyang District, Beijing, China 100025
3. Department of Radiology, Xiangya Hospital, Central South University, No. 87 Xiangya Rd, Changsha 410008, Hunan, P. R. China
4. School of Computer Science and Engineering, Central South University, Changsha, 410083, Hunan, P. R. China
5. Department of Radiology, Huadong Hospital Affiliated to Fudan University, Shanghai, China
6. Paul C. Lauterbur Research Center for Biomedical Imaging, Institute of Biomedical and Health Engineering, Shenzhen Institutes of Advanced Technology, Chinese Academy of Sciences, Shenzhen 518055, China
7. Clinical Research Center for Medical Imaging in Hunan Province, Changsha, Hunan Province, China

*These authors contributed equally to this work.
[†]These authors contributed equally as co-corresponding authors.

**Corresponding Authors:**
Wei Zhao, E-mail: wei.zhao@csu.edu.cn, Tel: 18670496192
Jun Liu, E-mail: junliu123@csu.edu.cn, Tel: 13787085002

**Supplementary Material 1.** CT acquisition and scanning parameters.

For Dataset 1, patients were performed with the following six scanners: GE Discovery CT750 HD, 64-slice LightSpeed VCT, Revolution CT (GE Medical Systems); Somatom Definition flash, Somatom Sensation-16, Somatom Force (Siemens Medical Solutions). For Dataset 2, patients were performed with the following four scanners: Somatom Definition Flash, Siemens, German; uCT780, United Imaging, Shanghai; Somatom Perspective 128, Siemens, German; Somatom Definition Force, Siemens, German. The acquisition parameters were as follows: tube voltage, 120 kVp; tube current, 100-200 mAs; pitch, 0.75–1.5. CT images were acquired in the supine position at full inspiration for all patients. Only plain and thin CT images were used in this study.

**Supplementary Material 2.** Descriptions of nine machine learning classifiers.

**a. Random Forest (RF)**

RF is used for both classification and regression tasks. It is an ensemble learning method that combines multiple decision trees to improve the accuracy and generalization of the model. The idea behind the RF algorithm is to create a large number of decision trees, each of which is trained on a random subset of the data and a random subset of the features. The predictions from all of the trees are then aggregated to produce a final output. This approach helps to reduce overfitting and improve the generalization of the model.

One of the key advantages of the RF algorithm is its ability to handle high-dimensional datasets with a large number of features. It is also relatively robust to noisy data and can handle missing values. Rf models are often used in applications such as image classification, text classification, and financial forecasting. They are also a popular choice for feature selection, as they can provide information about which features are the most important for making accurate predictions.

Overall, the RF algorithm is a powerful and versatile machine learning method that is widely used in both research and industry. Its ability to handle complex datasets and produce accurate predictions has made it a popular choice for a wide range of applications.

**b. K-nearest neighbors (KNN)**

KNN is a non-parametric and instance-based method, meaning that it makes predictions based on the characteristics of the closest training examples in the feature space. The KNN algorithm works by calculating the distance between the query instance and all of the training instances. The K nearest neighbors of the query instance are then identified, and the output of the algorithm is determined based on the majority

class (in classification tasks) or the average of the values (in regression tasks) of these neighbors.

One of the key advantages of the KNN algorithm is its simplicity and ease of implementation. It is also a non-parametric method, which means that it can handle data that does not conform to a specific distribution. KNN is also a very interpretable algorithm, as the predictions are based on the actual data points rather than a black-box model.

However, the KNN algorithm can be sensitive to the choice of K, which can greatly affect the quality of the predictions. It can also be computationally expensive for large datasets, as it requires calculating the distance between the query instance and all of the training instances.

**c. Logistic Regression (LR)**

LR is a statistical method used for binary classification, where the goal is to predict the probability of an event occurring. It is a type of linear regression that uses a logistic function to transform the output of the linear regression into a probability value between 0 and 1. In LR, the input features are used to calculate a linear combination, which is then transformed by the logistic function to produce the predicted probability of the event. The logistic function is a non-linear function that produces an S-shaped curve, which is useful for mapping the linear combination onto the probability scale.

The LR algorithm is trained using a labeled dataset, where each instance is associated with a binary label indicating the presence or absence of the event. The algorithm estimates the parameters of the logistic function using maximum likelihood estimation, which is a statistical method for finding the parameters that are most likely to have produced the observed data.

One of the key advantages of LR is its interpretability. The output of the algorithm is a probability value, which can be easily understood and interpreted by humans. LR is also computationally efficient and can be trained on large datasets.

LR is commonly used in applications such as credit scoring, fraud detection, and medical diagnosis. It is a simple yet powerful machine learning method that is well-suited to binary classification tasks.

**d. Extremely Randomized Trees (ExtraTrees)**

A machine learning algorithm that is used for both classification and regression problems. It is a type of ensemble learning method, meaning it combines multiple models together to produce a more accurate final prediction. ExtraTrees is similar to other decision tree-based algorithms, such as Random Forest, but it has some important differences. In ExtraTrees, the trees are built using a random subset of the features, and at each split in the tree, the algorithm randomly selects a subset of candidate thresholds

to determine the best split. These two randomization techniques help to reduce overfitting and improve the robustness of the model to noise and outliers.

ExtraTrees is also computationally efficient, since the trees are built independently of each other, and the randomization reduces the number of candidate thresholds that need to be evaluated. As a result, it can handle large datasets with high-dimensional feature spaces. It is particularly useful in situations where other methods may overfit or struggle with noisy data.

**e. CatBoost**

CatBoost is an open-source machine learning algorithm that is designed to work well with both categorical and numerical data. It was developed by Yandex, a Russian search engine company, and was first released in 2017.

One of the key features of CatBoost is its ability to handle categorical features with high cardinality, which is a common challenge in many real-world datasets. It does this by using an algorithm called "ordered boosting", which works by constructing decision trees that split on categorical features in a way that preserves the natural ordering of the categories.

In addition to its strong performance on datasets with categorical features, CatBoost also has several other useful features, such as built-in cross-validation, early stopping, and the ability to handle missing values in the data.

**f. eXtreme Gradient Boosting (XGBoost)**

XGBoost is used for supervised learning tasks, such as classification, regression, and ranking. XGBoost is a type of gradient boosting algorithm, which works by iteratively training a series of weak models (usually decision trees) and combining their predictions to produce a final output. XGBoost uses a regularized version of gradient boosting that includes both L1 and L2 regularization, which helps to prevent overfitting and improve generalization.

One of the key features of XGBoost is its scalability, which allows it to handle very large datasets and train models quickly. It also has built-in cross-validation, early stopping, and support for missing values in the data.

Overall, XGBoost is a powerful and flexible algorithm that has become very popular in the machine learning community, winning many machine learning competitions on platforms such as Kaggle. It is widely used in industry and academia for a wide range of tasks, and is known for its high predictive accuracy and speed.

**g. NeuralNetFastAI**

NeuralNetFastAI is a machine learning library that provides an easy-to-use interface for building and training neural networks. It is built on top of PyTorch, one of

the most popular deep learning frameworks, and is designed to make it easy for practitioners to build state-of-the-art deep learning models without requiring extensive knowledge of the underlying math and programming.

One of the key features of NeuralNetFastAI is its flexibility, which allows users to customize their neural network architectures and training processes in a variety of ways. NeuralNetFastAI is a powerful and flexible library that is well-suited to a wide range of deep learning tasks, including image and text classification, object detection, and natural language processing.

**h. NeuralNetTorch**

NeuralNetTorch is a machine learning library that provides an interface for building and training neural networks using PyTorch, one of the most popular deep learning frameworks. It is designed to make it easier for practitioners to build, train, and experiment with neural network models, while also providing flexibility for customizing the architecture and training process.

One of the key features of NeuralNetTorch is its modular design, which allows users to build complex neural network architectures by stacking together a variety of different layer types. Users can choose from a range of activation functions, convolutional layers, recurrent layers, and pooling layers, among other options, to build customized networks that are tailored to their specific needs.

NeuralNetTorch also provides a number of useful tools for working with large datasets, including support for data augmentation and distributed training. It also has built-in functionality for visualizing and interpreting the results of model training, making it easier for practitioners to debug and fine-tune their models.

**i. Light Gradient Boosting Machine (LightGBM)**

LightGBM is designed to train gradient boosting decision tree models. It was developed by Microsoft and is known for its speed, scalability, and high accuracy.

One of the key features of LightGBM is its ability to handle large datasets with high dimensionality. It does this by using a technique called "gradient-based one-side sampling", which samples the data based on the gradients of the loss function and the distribution of the data, resulting in faster and more efficient training.

LightGBM also uses a technique called "leaf-wise tree growth", which grows the decision trees in a way that prioritizes the leaf nodes with the most significant gradients, resulting in deeper trees and more accurate predictions.

In addition to its speed and accuracy, LightGBM has a number of other useful features, such as built-in support for categorical features, early stopping, and parallel training on multi-core CPUs.

# Supplementary Figures

**Supplementary Fig.S1** Use minimum redundancy maximum relevance (mRMR) to identify the most important features on the basis of a heuristic scoring criterion, and only the top ranked features were kept. Clearer images are shown in separate figures (Fig.S1-1, Fig.S1-2)

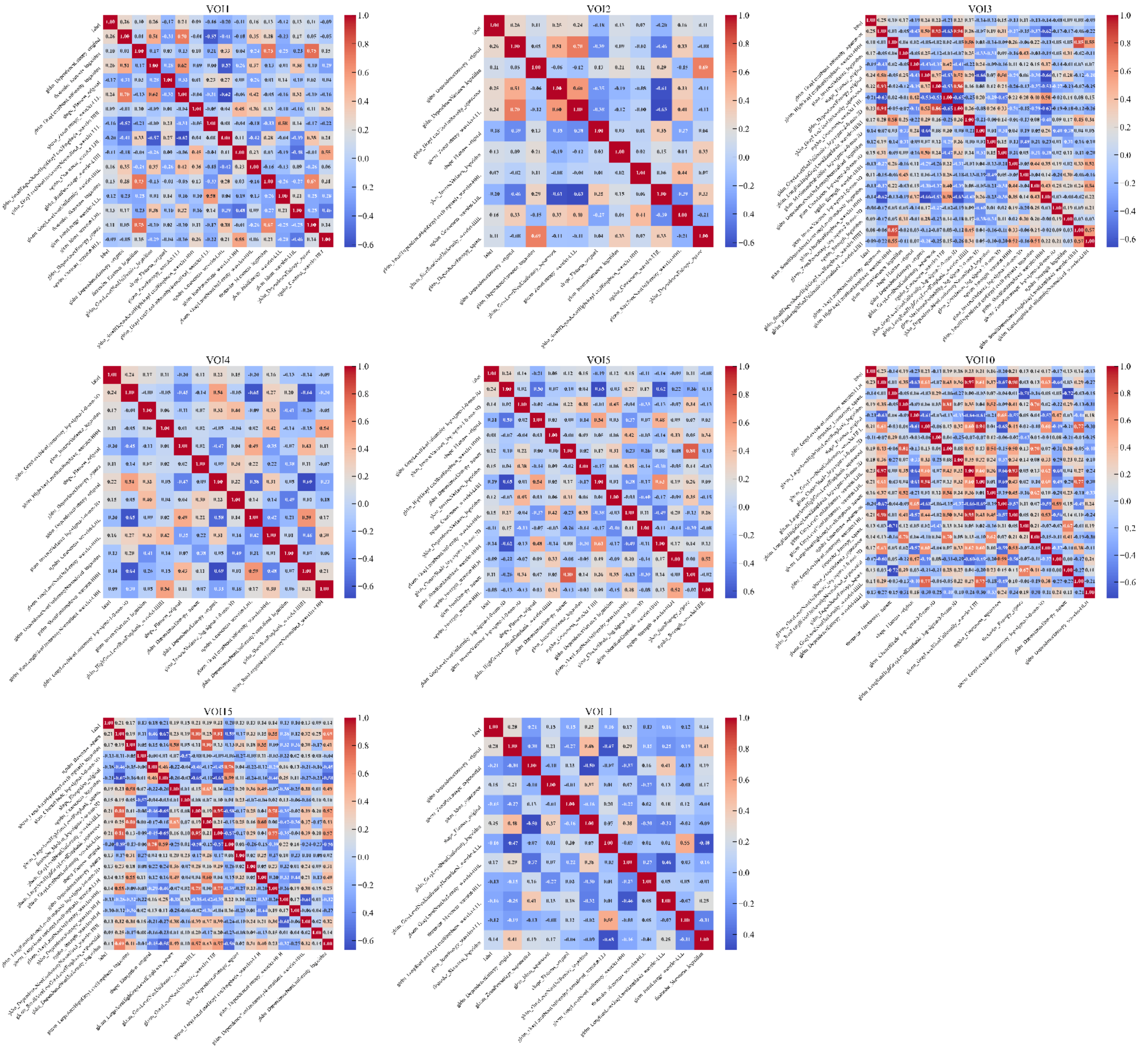

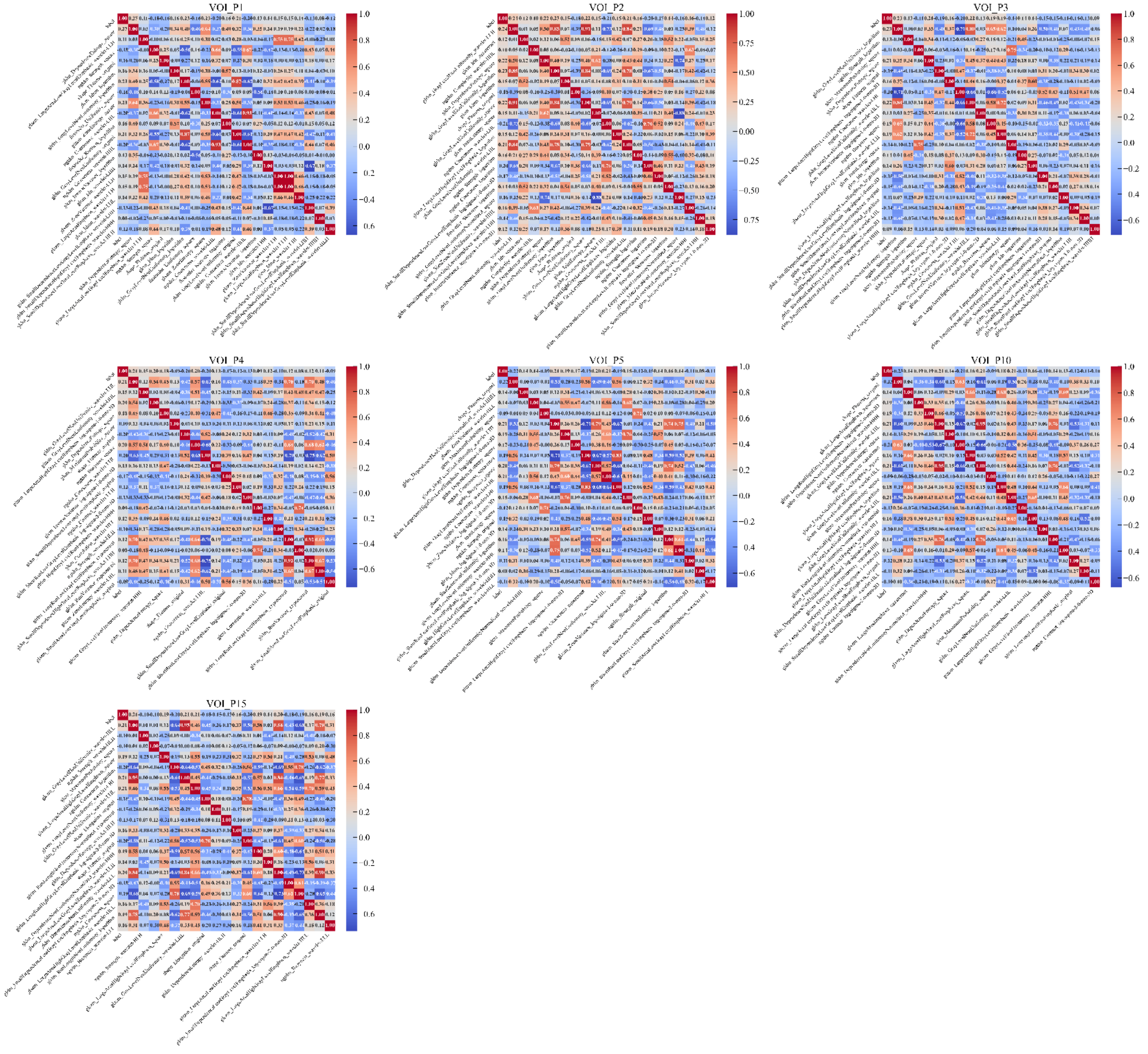
VOI_P1
VOI_P2
VOI_P3
VOI_P4
VOI_P5
VOI_P10
VOI_P15

**Supplementary Fig.S2. The final selected radiomic features in each VOI.**

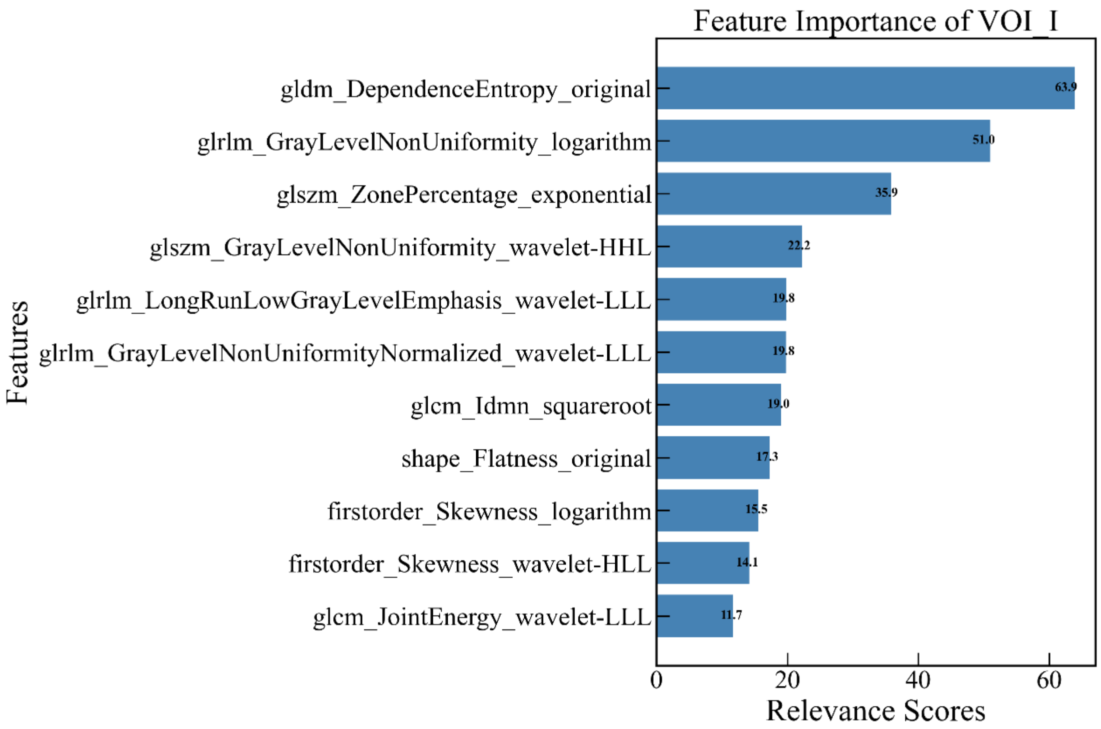


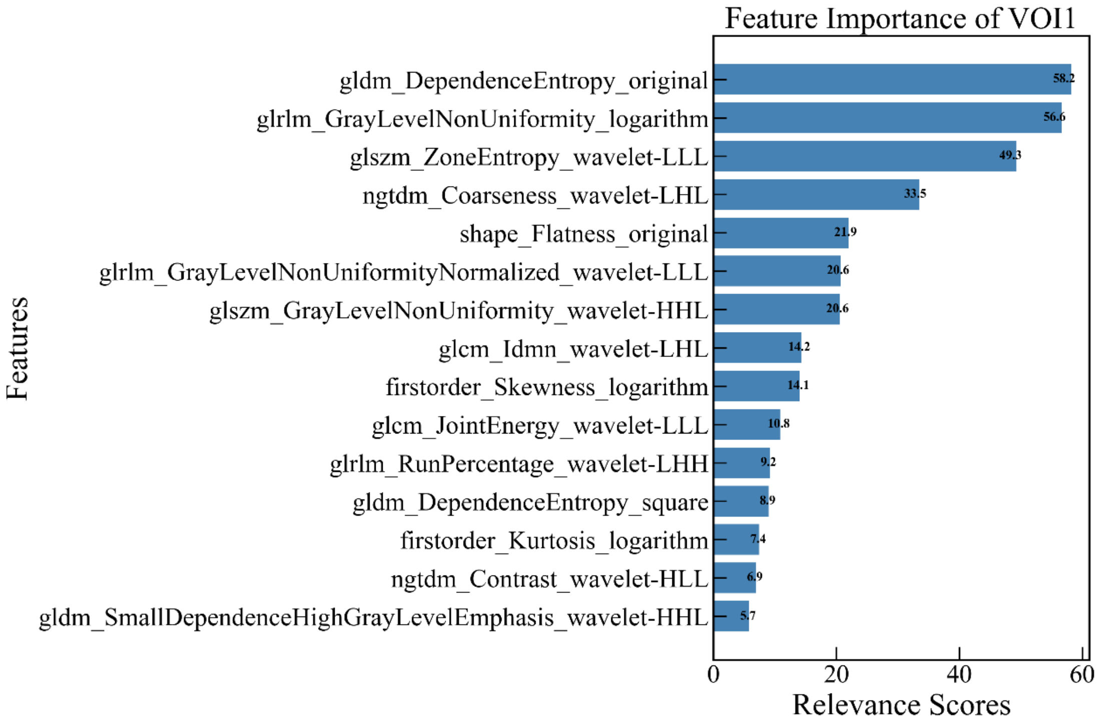

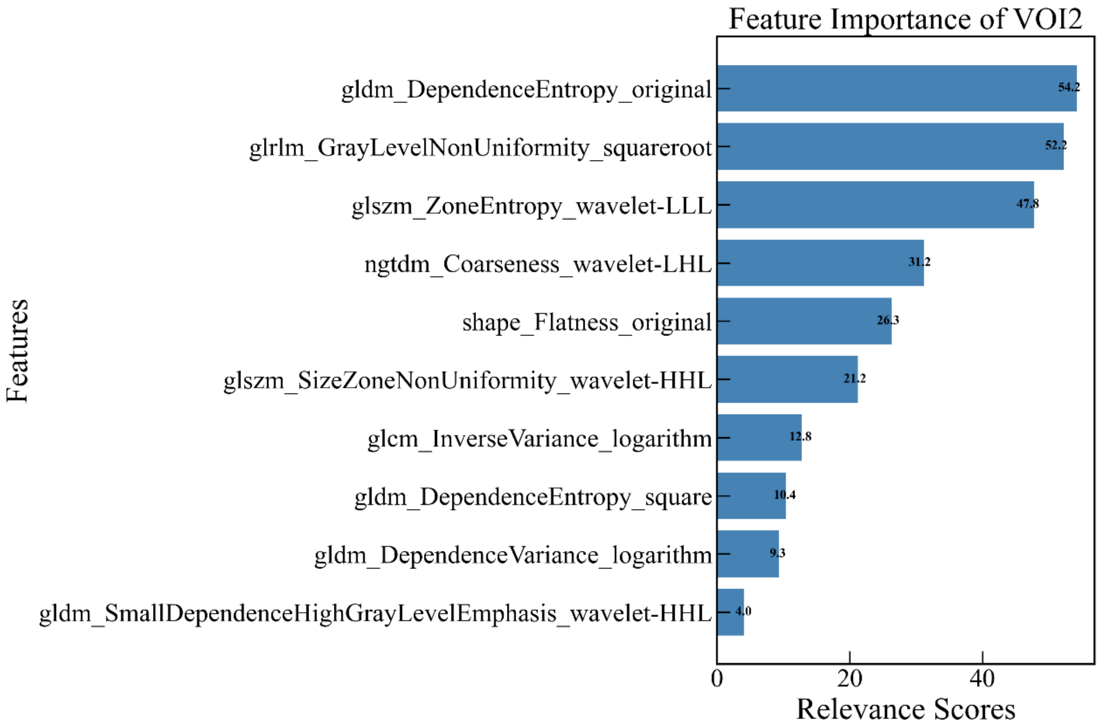
Feature Importance of VOI2
Features
gldm_DependenceEntropy_original
glrlm_GrayLevelNonUniformity_squareroot
glszm_ZoneEntropy_wavelet-LLL
ngtdm_Coarseness_wavelet-LHL
shape_Flatness_original
glszm_SizeZoneNonUniformity_wavelet-HHL
glcm_InverseVariance_logarithm
gldm_DependenceEntropy_square
gldm_DependenceVariance_logarithm
gldm_SmallDependenceHighGrayLevelEmphasis_wavelet-HHL
54.2
52.2
47.8
31.2
26.3
21.2
12.8
10.4
9.3
4.0
0
20
40
Relevance Scores

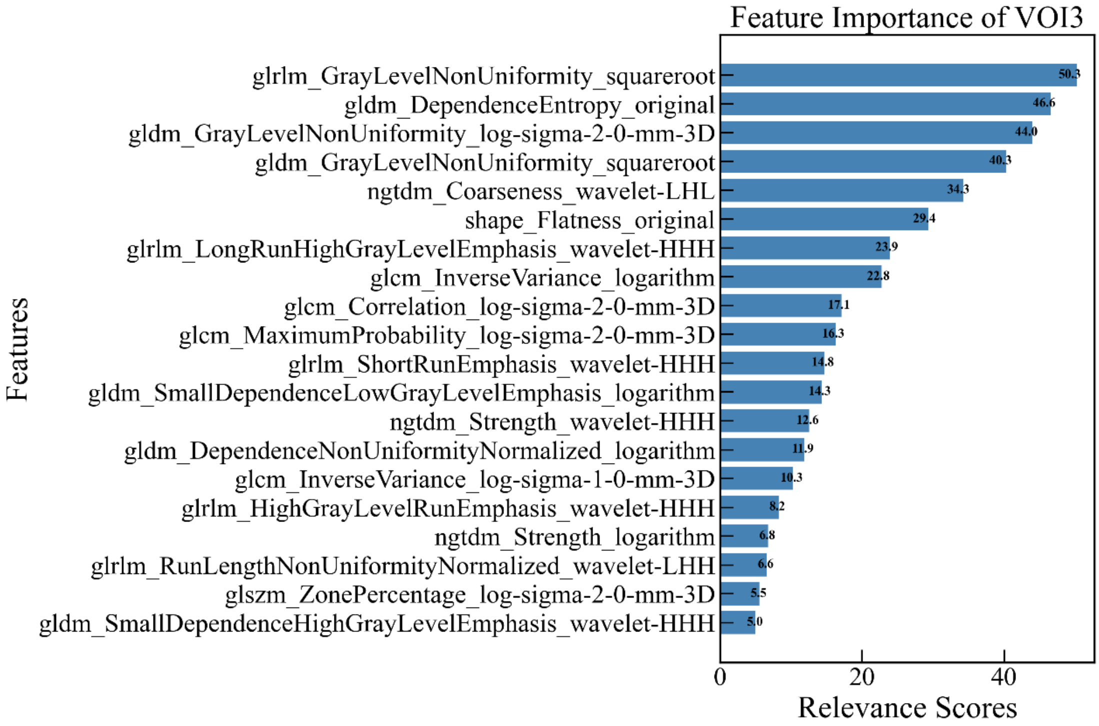
Feature Importance of VOI3
Features
glrlm_GrayLevelNonUniformity_squareroot
gldm_DependenceEntropy_original
gldm_GrayLevelNonUniformity_log-sigma-2-0-mm-3D
gldm_GrayLevelNonUniformity_squareroot
ngtdm_Coarseness_wavelet-LHL
shape_Flatness_original
glrlm_LongRunHighGrayLevelEmphasis_wavelet-HHH
glcm_InverseVariance_logarithm
glcm_Correlation_log-sigma-2-0-mm-3D
glcm_MaximumProbability_log-sigma-2-0-mm-3D
glrlm_ShortRunEmphasis_wavelet-HHH
gldm_SmallDependenceLowGrayLevelEmphasis_logarithm
ngtdm_Strength_wavelet-HHH
gldm_DependenceNonUniformityNormalized_logarithm
glcm_InverseVariance_log-sigma-1-0-mm-3D
glrlm_HighGrayLevelRunEmphasis_wavelet-HHH
ngtdm_Strength_logarithm
glrlm_RunLengthNonUniformityNormalized_wavelet-LHH
glszm_ZonePercentage_log-sigma-2-0-mm-3D
gldm_SmallDependenceHighGrayLevelEmphasis_wavelet-HHH
50.3
46.6
44.0
40.3
34.3
29.4
23.9
22.8
17.1
16.3
14.8
14.3
12.6
11.9
10.3
8.2
6.8
6.6
5.5
5.0
0
20
40
Relevance Scores

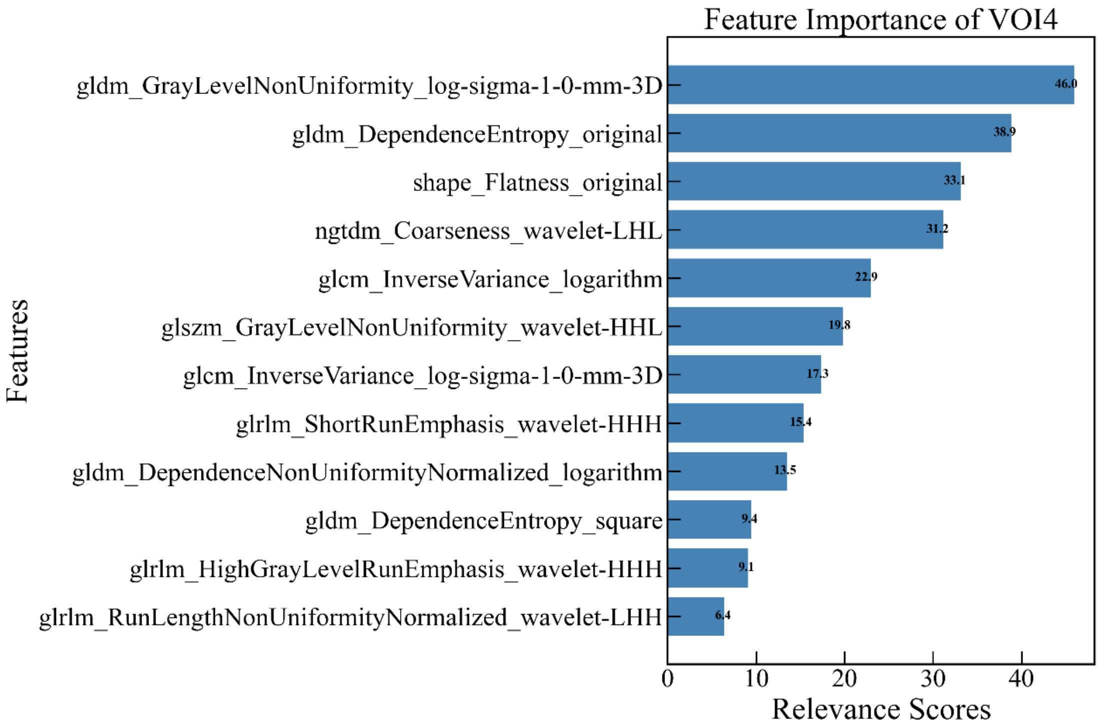
Feature Importance of VOI4
gldm_GrayLevelNonUniformity_log-sigma-1-0-mm-3D
gldm_DependenceEntropy_original
shape_Flatness_original
ngtdm_Coarseness_wavelet-LHL
glcm_InverseVariance_logarithm
glszm_GrayLevelNonUniformity_wavelet-HHL
glcm_InverseVariance_log-sigma-1-0-mm-3D
glrlm_ShortRunEmphasis_wavelet-HHH
gldm_DependenceNonUniformityNormalized_logarithm
gldm_DependenceEntropy_square
glrlm_HighGrayLevelRunEmphasis_wavelet-HHH
glrlm_RunLengthNonUniformityNormalized_wavelet-LHH
46.0
38.9
33.1
31.2
22.9
19.8
17.3
15.4
13.5
9.4
9.1
6.4
Features
0
10
20
30
40
Relevance Scores

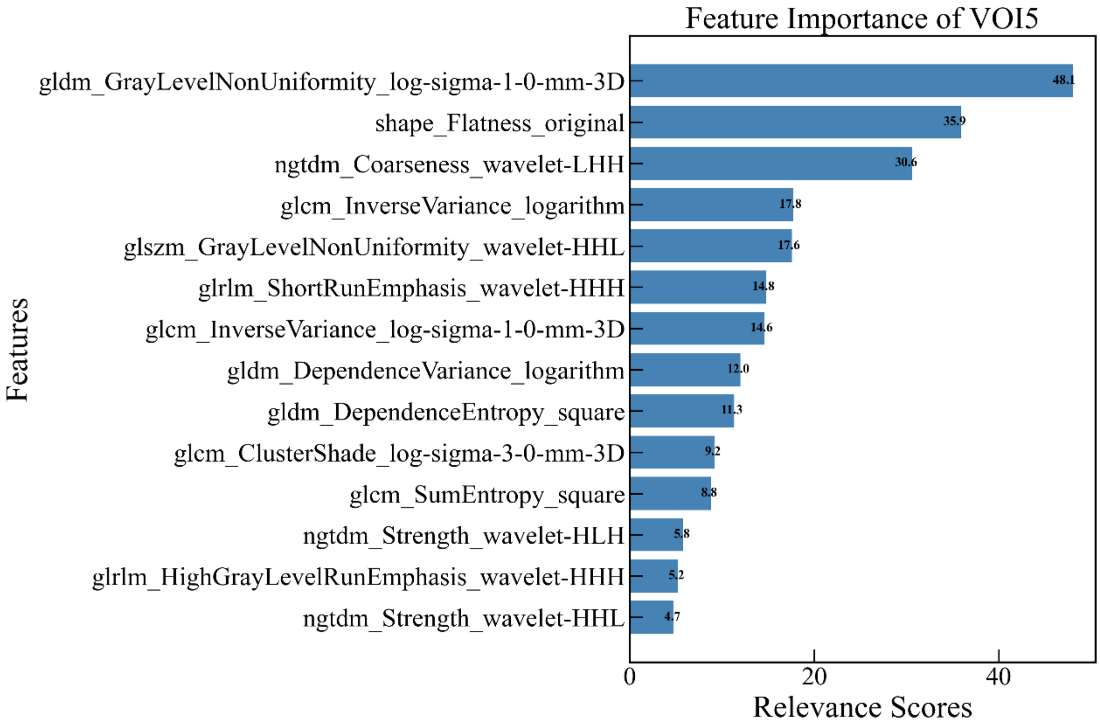
Feature Importance of VOI5
gldm_GrayLevelNonUniformity_log-sigma-1-0-mm-3D
shape_Flatness_original
ngtdm_Coarseness_wavelet-LHH
glcm_InverseVariance_logarithm
glszm_GrayLevelNonUniformity_wavelet-HHL
glrlm_ShortRunEmphasis_wavelet-HHH
glcm_InverseVariance_log-sigma-1-0-mm-3D
gldm_DependenceVariance_logarithm
gldm_DependenceEntropy_square
glcm_ClusterShade_log-sigma-3-0-mm-3D
glcm_SumEntropy_square
ngtdm_Strength_wavelet-HLH
glrlm_HighGrayLevelRunEmphasis_wavelet-HHH
ngtdm_Strength_wavelet-HHL
48.1
35.9
30.6
17.8
17.6
14.8
14.6
12.0
11.3
9.2
8.8
5.8
5.2
4.7
Features
0
20
40
Relevance Scores

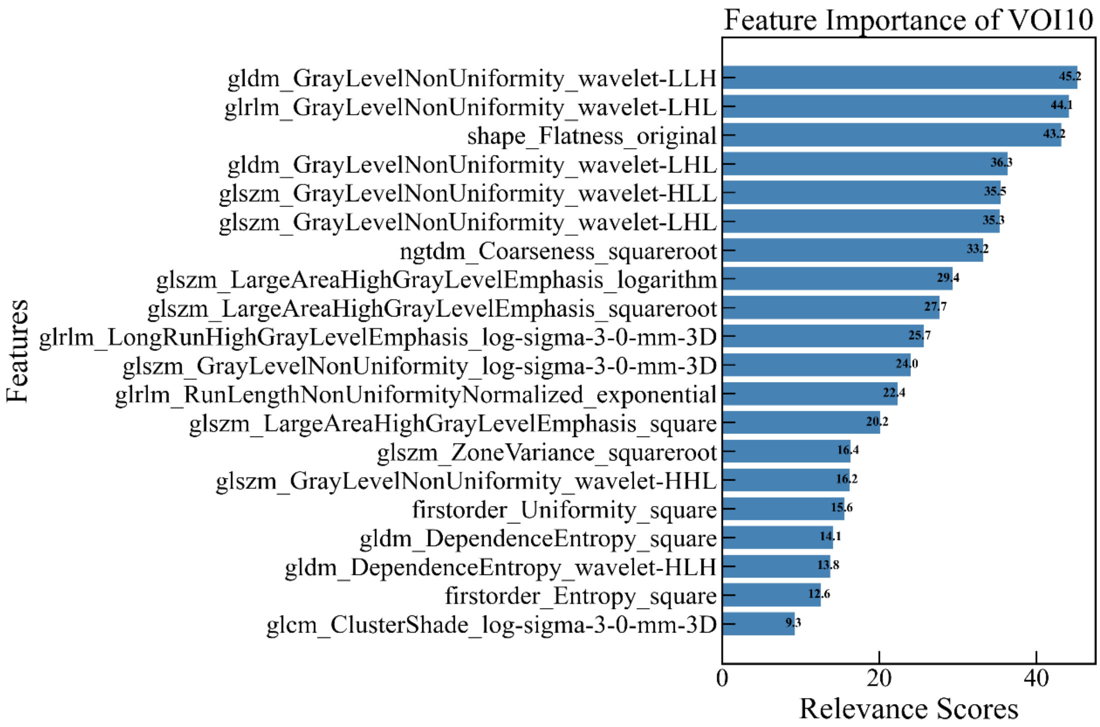
Feature Importance of VOI10
gldm_GrayLevelNonUniformity_wavelet-LLH
glrlm_GrayLevelNonUniformity_wavelet-LHL
shape_Flatness_original
gldm_GrayLevelNonUniformity_wavelet-LHL
glszm_GrayLevelNonUniformity_wavelet-HLL
glszm_GrayLevelNonUniformity_wavelet-LHL
ngtdm_Coarseness_squareroot
glszm_LargeAreaHighGrayLevelEmphasis_logarithm
glszm_LargeAreaHighGrayLevelEmphasis_squareroot
glrlm_LongRunHighGrayLevelEmphasis_log-sigma-3-0-mm-3D
glszm_GrayLevelNonUniformity_log-sigma-3-0-mm-3D
glrlm_RunLengthNonUniformityNormalized_exponential
glszm_LargeAreaHighGrayLevelEmphasis_square
glszm_ZoneVariance_squareroot
glszm_GrayLevelNonUniformity_wavelet-HHL
firstorder_Uniformity_square
gldm_DependenceEntropy_square
gldm_DependenceEntropy_wavelet-HLH
firstorder_Entropy_square
glcm_ClusterShade_log-sigma-3-0-mm-3D
45.2
44.1
43.2
36.3
35.5
35.3
33.2
29.4
27.7
25.7
24.0
22.4
20.2
16.4
16.2
15.6
14.1
13.8
12.6
9.3
Features
0
20
40
Relevance Scores

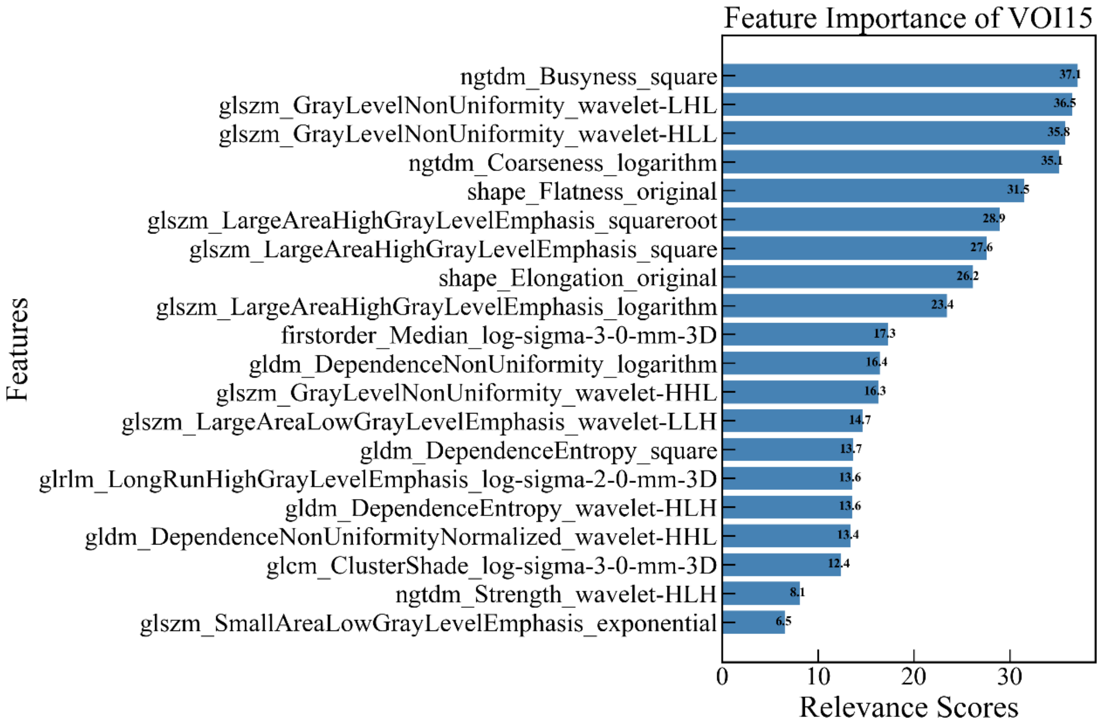
Feature Importance of VOI15
ngtdm_Busyness_square
glszm_GrayLevelNonUniformity_wavelet-LHL
glszm_GrayLevelNonUniformity_wavelet-HLL
ngtdm_Coarseness_logarithm
shape_Flatness_original
glszm_LargeAreaHighGrayLevelEmphasis_squareroot
glszm_LargeAreaHighGrayLevelEmphasis_square
shape_Elongation_original
glszm_LargeAreaHighGrayLevelEmphasis_logarithm
firstorder_Median_log-sigma-3-0-mm-3D
gldm_DependenceNonUniformity_logarithm
glszm_GrayLevelNonUniformity_wavelet-HHL
glszm_LargeAreaLowGrayLevelEmphasis_wavelet-LLH
gldm_DependenceEntropy_square
glrlm_LongRunHighGrayLevelEmphasis_log-sigma-2-0-mm-3D
gldm_DependenceEntropy_wavelet-HLH
gldm_DependenceNonUniformityNormalized_wavelet-HHL
glcm_ClusterShade_log-sigma-3-0-mm-3D
ngtdm_Strength_wavelet-HLH
glszm_SmallAreaLowGrayLevelEmphasis_exponential
37.1
36.5
35.8
35.1
31.5
28.9
27.6
26.2
23.4
17.3
16.4
16.3
14.7
13.7
13.6
13.6
13.4
12.4
8.1
6.5
Features
0
10
20
30
Relevance Scores

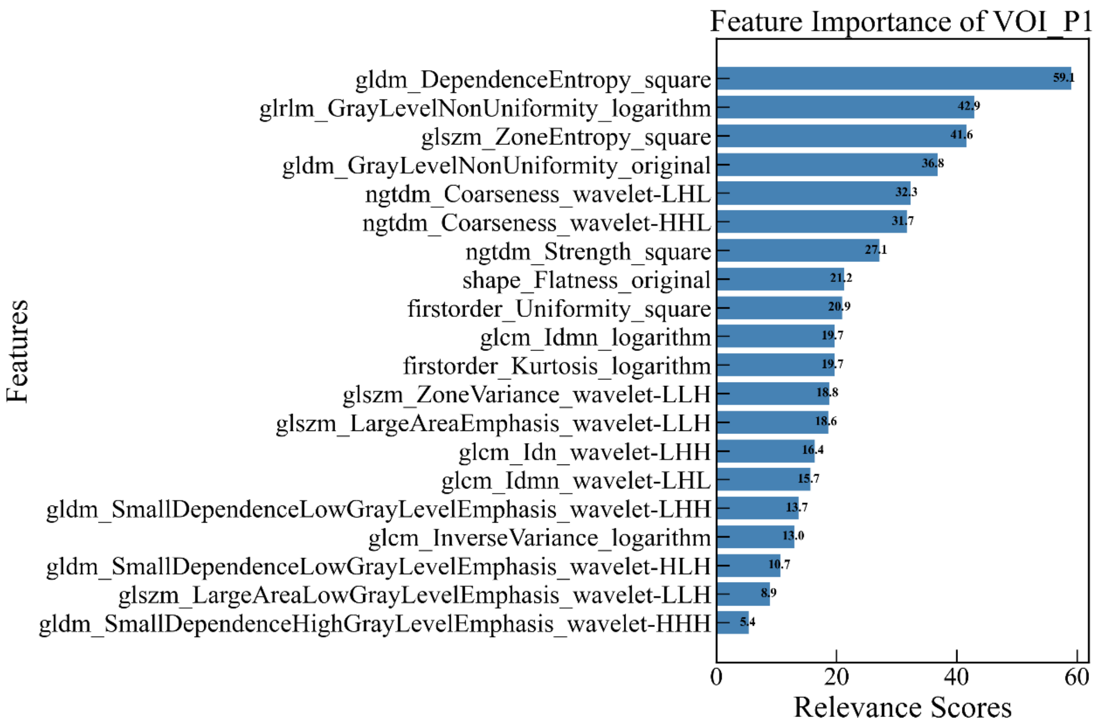
Feature Importance of VOI_P1
Features
Relevance Scores
gldm_DependenceEntropy_square 59.1
glrlm_GrayLevelNonUniformity_logarithm 42.9
glszm_ZoneEntropy_square 41.6
gldm_GrayLevelNonUniformity_original 36.8
ngtdm_Coarseness_wavelet-LHL 32.3
ngtdm_Coarseness_wavelet-HHL 31.7
ngtdm_Strength_square 27.1
shape_Flatness_original 21.2
firstorder_Uniformity_square 20.9
glcm_Idmn_logarithm 19.7
firstorder_Kurtosis_logarithm 19.7
glszm_ZoneVariance_wavelet-LLH 18.8
glszm_LargeAreaEmphasis_wavelet-LLH 18.6
glcm_Idn_wavelet-LHH 16.4
glcm_Idmn_wavelet-LHL 15.7
gldm_SmallDependenceLowGrayLevelEmphasis_wavelet-LHH 13.7
glcm_InverseVariance_logarithm 13.0
gldm_SmallDependenceLowGrayLevelEmphasis_wavelet-HLH 10.7
glszm_LargeAreaLowGrayLevelEmphasis_wavelet-LLH 8.9
gldm_SmallDependenceHighGrayLevelEmphasis_wavelet-HHH 5.4
0
20
40
60

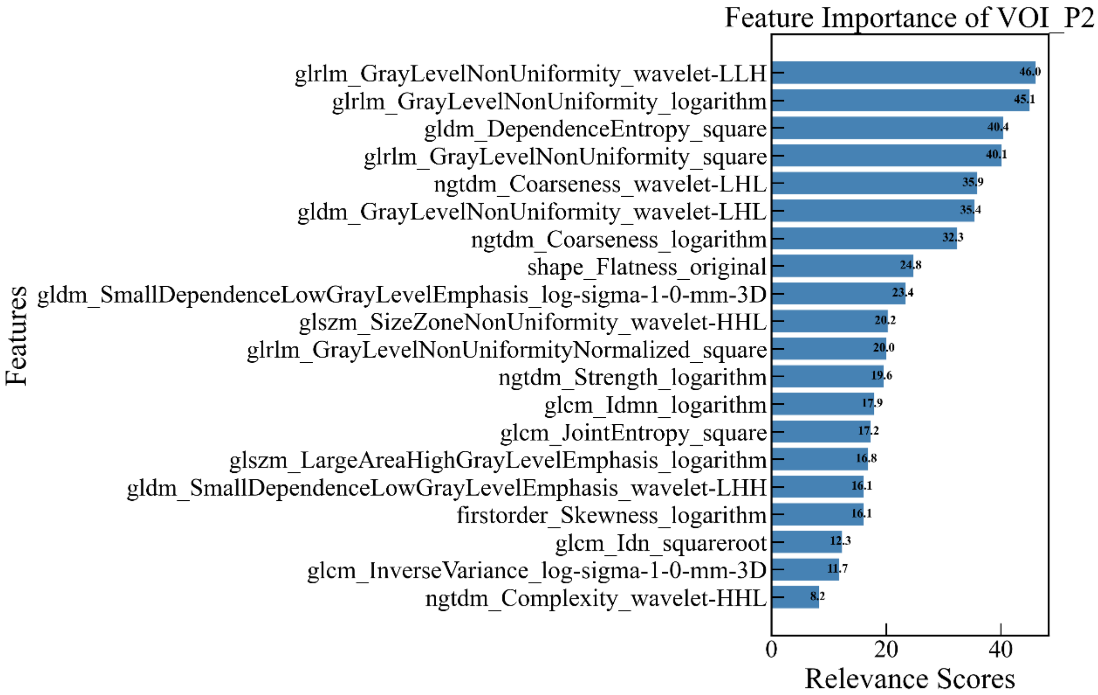
Feature Importance of VOI_P2
Features
Relevance Scores
glrlm_GrayLevelNonUniformity_wavelet-LLH 46.0
glrlm_GrayLevelNonUniformity_logarithm 45.1
gldm_DependenceEntropy_square 40.4
glrlm_GrayLevelNonUniformity_square 40.1
ngtdm_Coarseness_wavelet-LHL 35.9
gldm_GrayLevelNonUniformity_wavelet-LHL 35.4
ngtdm_Coarseness_logarithm 32.3
shape_Flatness_original 24.8
gldm_SmallDependenceLowGrayLevelEmphasis_log-sigma-1-0-mm-3D 23.4
glszm_SizeZoneNonUniformity_wavelet-HHL 20.2
glrlm_GrayLevelNonUniformityNormalized_square 20.0
ngtdm_Strength_logarithm 19.6
glcm_Idmn_logarithm 17.9
glcm_JointEntropy_square 17.2
glszm_LargeAreaHighGrayLevelEmphasis_logarithm 16.8
gldm_SmallDependenceLowGrayLevelEmphasis_wavelet-LHH 16.1
firstorder_Skewness_logarithm 16.1
glcm_Idn_squareroot 12.3
glcm_InverseVariance_log-sigma-1-0-mm-3D 11.7
ngtdm_Complexity_wavelet-HHL 8.2
0
20
40

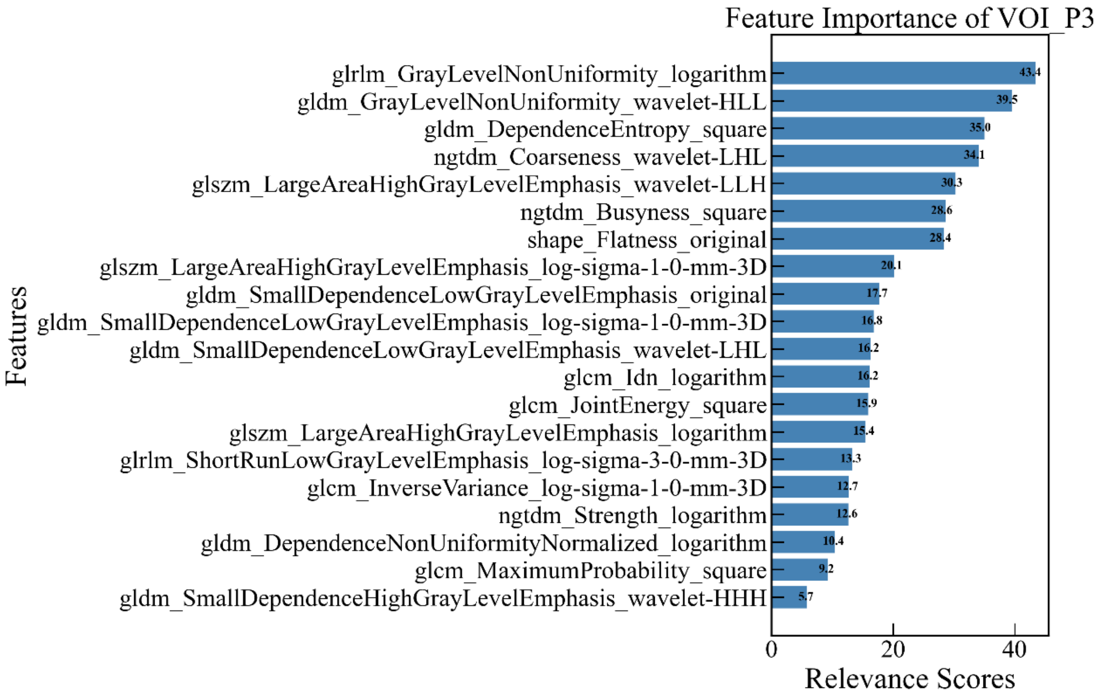
Feature Importance of VOI_P3
glrlm_GrayLevelNonUniformity_logarithm
gldm_GrayLevelNonUniformity_wavelet-HLL
gldm_DependenceEntropy_square
ngtdm_Coarseness_wavelet-LHL
glszm_LargeAreaHighGrayLevelEmphasis_wavelet-LLH
ngtdm_Busyness_square
shape_Flatness_original
glszm_LargeAreaHighGrayLevelEmphasis_log-sigma-1-0-mm-3D
gldm_SmallDependenceLowGrayLevelEmphasis_original
gldm_SmallDependenceLowGrayLevelEmphasis_log-sigma-1-0-mm-3D
gldm_SmallDependenceLowGrayLevelEmphasis_wavelet-LHL
glcm_Idn_logarithm
glcm_JointEnergy_square
glszm_LargeAreaHighGrayLevelEmphasis_logarithm
glrlm_ShortRunLowGrayLevelEmphasis_log-sigma-3-0-mm-3D
glcm_InverseVariance_log-sigma-1-0-mm-3D
ngtdm_Strength_logarithm
gldm_DependenceNonUniformityNormalized_logarithm
glcm_MaximumProbability_square
gldm_SmallDependenceHighGrayLevelEmphasis_wavelet-HHH
43.4
39.5
35.0
34.1
30.3
28.6
28.4
20.1
17.7
16.8
16.2
16.2
15.9
15.4
13.3
12.7
12.6
10.4
9.2
5.7
Features
0
20
40
Relevance Scores

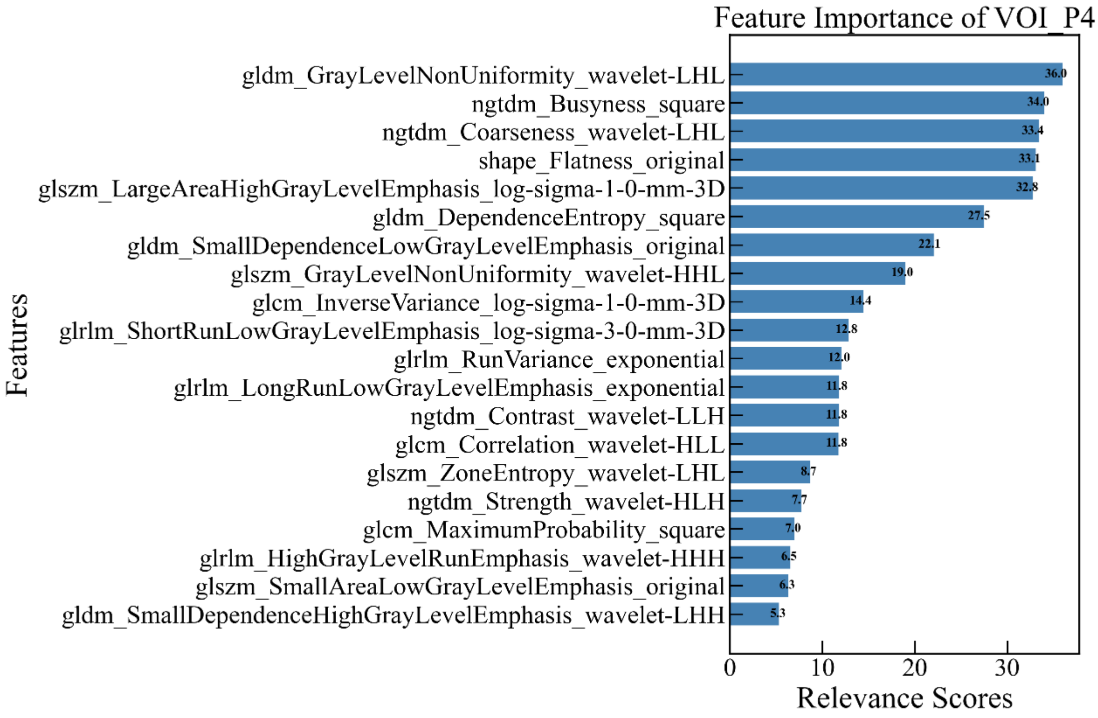
Feature Importance of VOI_P4
gldm_GrayLevelNonUniformity_wavelet-LHL
ngtdm_Busyness_square
ngtdm_Coarseness_wavelet-LHL
shape_Flatness_original
glszm_LargeAreaHighGrayLevelEmphasis_log-sigma-1-0-mm-3D
gldm_DependenceEntropy_square
gldm_SmallDependenceLowGrayLevelEmphasis_original
glszm_GrayLevelNonUniformity_wavelet-HHL
glcm_InverseVariance_log-sigma-1-0-mm-3D
glrlm_ShortRunLowGrayLevelEmphasis_log-sigma-3-0-mm-3D
glrlm_RunVariance_exponential
glrlm_LongRunLowGrayLevelEmphasis_exponential
ngtdm_Contrast_wavelet-LLH
glcm_Correlation_wavelet-HLL
glszm_ZoneEntropy_wavelet-LHL
ngtdm_Strength_wavelet-HLH
glcm_MaximumProbability_square
glrlm_HighGrayLevelRunEmphasis_wavelet-HHH
glszm_SmallAreaLowGrayLevelEmphasis_original
gldm_SmallDependenceHighGrayLevelEmphasis_wavelet-LHH
36.0
34.0
33.4
33.1
32.8
27.5
22.1
19.0
14.4
12.8
12.0
11.8
11.8
11.8
8.7
7.7
7.0
6.5
6.3
5.3
Features
0
10
20
30
Relevance Scores

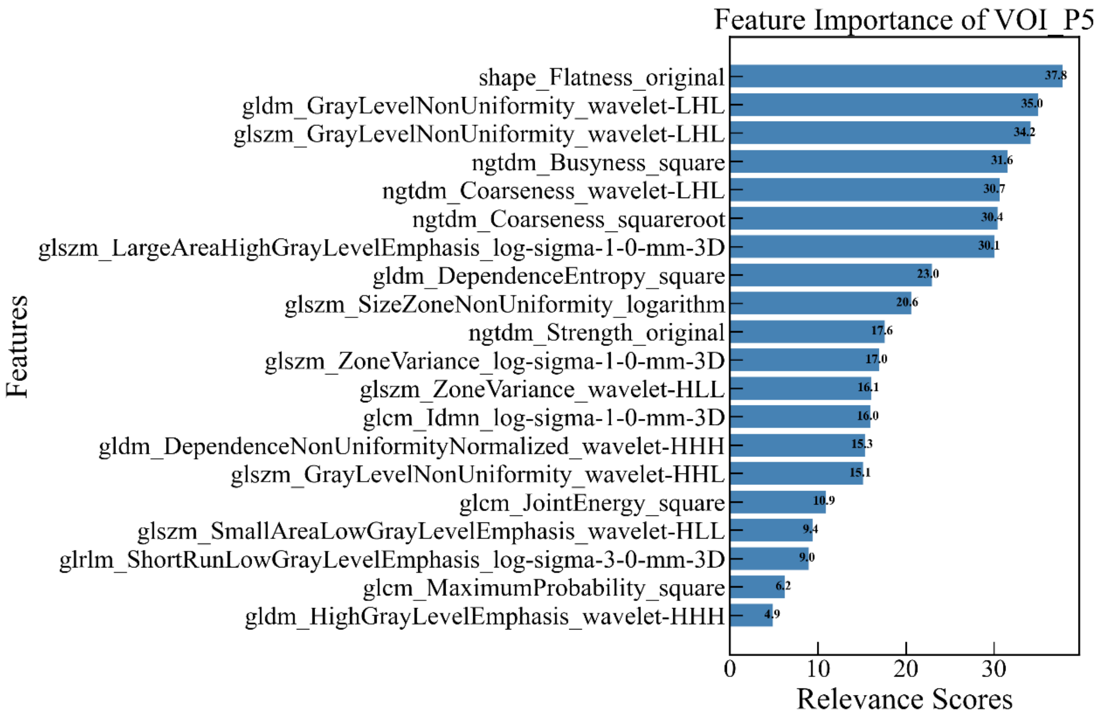
Feature Importance of VOI_P5
Features
shape_Flatness_original
gldm_GrayLevelNonUniformity_wavelet-LHL
glszm_GrayLevelNonUniformity_wavelet-LHL
ngtdm_Busyness_square
ngtdm_Coarseness_wavelet-LHL
ngtdm_Coarseness_squareroot
glszm_LargeAreaHighGrayLevelEmphasis_log-sigma-1-0-mm-3D
gldm_DependenceEntropy_square
glszm_SizeZoneNonUniformity_logarithm
ngtdm_Strength_original
glszm_ZoneVariance_log-sigma-1-0-mm-3D
glszm_ZoneVariance_wavelet-HLL
glcm_Idmn_log-sigma-1-0-mm-3D
gldm_DependenceNonUniformityNormalized_wavelet-HHH
glszm_GrayLevelNonUniformity_wavelet-HHL
glcm_JointEnergy_square
glszm_SmallAreaLowGrayLevelEmphasis_wavelet-HLL
glrlm_ShortRunLowGrayLevelEmphasis_log-sigma-3-0-mm-3D
glcm_MaximumProbability_square
gldm_HighGrayLevelEmphasis_wavelet-HHH
37.8
35.0
34.2
31.6
30.7
30.4
30.1
23.0
20.6
17.6
17.0
16.1
16.0
15.3
15.1
10.9
9.4
9.0
6.2
4.9
0
10
20
30
Relevance Scores

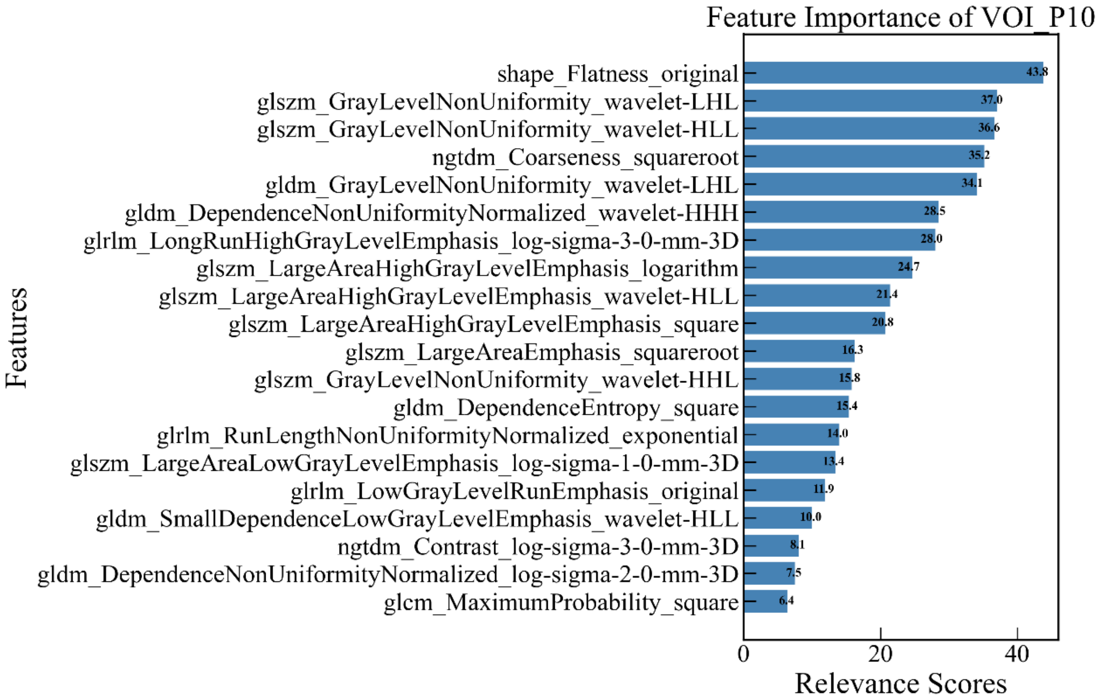
Feature Importance of VOI_P10
Features
shape_Flatness_original
glszm_GrayLevelNonUniformity_wavelet-LHL
glszm_GrayLevelNonUniformity_wavelet-HLL
ngtdm_Coarseness_squareroot
gldm_GrayLevelNonUniformity_wavelet-LHL
gldm_DependenceNonUniformityNormalized_wavelet-HHH
glrlm_LongRunHighGrayLevelEmphasis_log-sigma-3-0-mm-3D
glszm_LargeAreaHighGrayLevelEmphasis_logarithm
glszm_LargeAreaHighGrayLevelEmphasis_wavelet-HLL
glszm_LargeAreaHighGrayLevelEmphasis_square
glszm_LargeAreaEmphasis_squareroot
glszm_GrayLevelNonUniformity_wavelet-HHL
gldm_DependenceEntropy_square
glrlm_RunLengthNonUniformityNormalized_exponential
glszm_LargeAreaLowGrayLevelEmphasis_log-sigma-1-0-mm-3D
glrlm_LowGrayLevelRunEmphasis_original
gldm_SmallDependenceLowGrayLevelEmphasis_wavelet-HLL
ngtdm_Contrast_log-sigma-3-0-mm-3D
gldm_DependenceNonUniformityNormalized_log-sigma-2-0-mm-3D
glcm_MaximumProbability_square
43.8
37.0
36.6
35.2
34.1
28.5
28.0
24.7
21.4
20.8
16.3
15.8
15.4
14.0
13.4
11.9
10.0
8.1
7.5
6.4
0
20
40
Relevance Scores

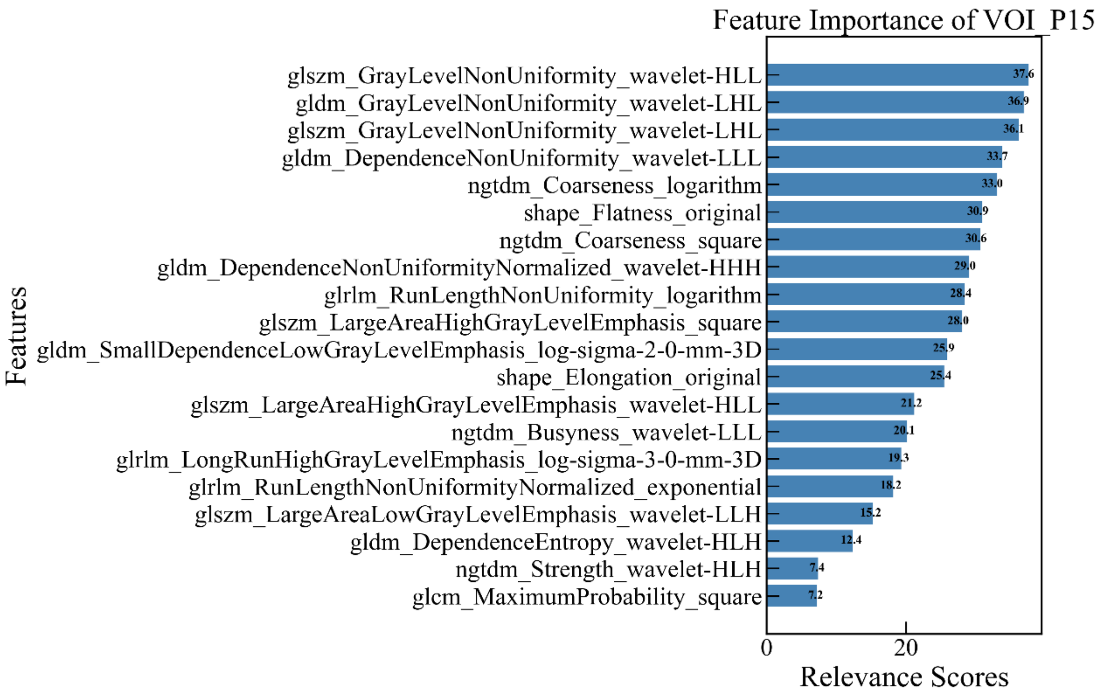
Feature Importance of VOI_P15
glszm_GrayLevelNonUniformity_wavelet-HLL
gldm_GrayLevelNonUniformity_wavelet-LHL
glszm_GrayLevelNonUniformity_wavelet-LHL
gldm_DependenceNonUniformity_wavelet-LLL
ngtdm_Coarseness_logarithm
shape_Flatness_original
ngtdm_Coarseness_square
gldm_DependenceNonUniformityNormalized_wavelet-HHH
glrlm_RunLengthNonUniformity_logarithm
glszm_LargeAreaHighGrayLevelEmphasis_square
gldm_SmallDependenceLowGrayLevelEmphasis_log-sigma-2-0-mm-3D
shape_Elongation_original
glszm_LargeAreaHighGrayLevelEmphasis_wavelet-HLL
ngtdm_Busyness_wavelet-LLL
glrlm_LongRunHighGrayLevelEmphasis_log-sigma-3-0-mm-3D
glrlm_RunLengthNonUniformityNormalized_exponential
glszm_LargeAreaLowGrayLevelEmphasis_wavelet-LLH
gldm_DependenceEntropy_wavelet-HLH
ngtdm_Strength_wavelet-HLH
glcm_MaximumProbability_square
37.6
36.9
36.1
33.7
33.0
30.9
30.6
29.0
28.4
28.0
25.9
25.4
21.2
20.1
19.3
18.2
15.2
12.4
7.4
7.2
Features
0
20
Relevance Scores

## Supplementary Tables

**Supplementary Table S1.** The optimal classifier corresponding to each VOI.

| *VOI | The optimal model |
|---|---|
| VOI_I | NeuralNetFastAI |
| VOI_P1 | ExtraTrees |
| VOI_P2 | CatBoost |
| VOI_P3 | XGBoost |
| VOI_P4 | ExtraTrees |
| VOI_P5 | XGBoost |
| VOI_P10 | XGBoost |
| VOI_P15 | CatBoost |
| VOI1 | NeuralNetTorch |
| VOI2 | ExtraTrees |
| VOI3 | ExtraTrees |
| VOI4 | LightGBM |
| VOI5 | NeuralNetTorch |
| VOI10 | NeuralNetTorch |
| VOI15 | CatBoost |

*VOI, volume of interest

**Supplementary Table S2.** Difference of AUC between VOI_I model and combined models in the internal testing set.

| | *VOI_I | VOI1 | VOI2 | VOI3 | VOI4 | VOI5 | VOI10 | VOI15 |
|---|---|---|---|---|---|---|---|---|
| VOI_I | 1.000 | 0.833 | 0.830 | 0.940 | 0.401 | 0.719 | 0.609 | 0.843 |
| VOI1 | 0.833 | 1.000 | 1.000 | 0.765 | 0.338 | 0.855 | 0.666 | 0.721 |
| VOI2 | 0.830 | 1.000 | 1.000 | 0.697 | 0.285 | 0.843 | 0.662 | 0.696 |
| VOI3 | 0.940 | 0.765 | 0.697 | 1.000 | 0.357 | 0.581 | 0.473 | 0.863 |
| VOI4 | 0.401 | 0.338 | 0.285 | 0.357 | 1.000 | 0.206 | 0.246 | 0.658 |
| VOI5 | 0.719 | 0.855 | 0.843 | 0.581 | 0.206 | 1.000 | 0.793 | 0.641 |
| VOI10 | 0.609 | 0.666 | 0.662 | 0.473 | 0.246 | 0.793 | 1.000 | 0.415 |
| VOI15 | 0.843 | 0.721 | 0.696 | 0.863 | 0.658 | 0.641 | 0.415 | 1.000 |

*VOI, volume of interest

**Supplementary Table S3.** Difference of AUC between VOI_I model and combined models in the external testing set.

| | *VOI_I | VOI1 | VOI2 | VOI3 | VOI4 | VOI5 | VOI10 | VOI15 |
|---|---|---|---|---|---|---|---|---|
| VOI_I | 1.000 | 0.971 | 0.629 | 0.002 | 0.000 | 0.597 | 0.008 | 0.001 |
| VOI1 | 0.971 | 1.000 | 0.597 | 0.001 | 0.000 | 0.574 | 0.007 | 0.001 |
| VOI2 | 0.629 | 0.597 | 1.000 | 0.001 | 0.000 | 0.308 | 0.003 | 0.000 |
| VOI3 | 0.003 | 0.001 | 0.001 | 1.000 | 0.502 | 0.005 | 0.409 | 0.917 |
| VOI4 | 0.001 | 0.000 | 0.000 | 0.502 | 1.000 | 0.001 | 0.231 | 0.708 |
| VOI5 | 0.597 | 0.574 | 0.308 | 0.004 | 0.001 | 1.000 | 0.020 | 0.002 |
| VOI10 | 0.010 | 0.008 | 0.005 | 0.409 | 0.231 | 0.020 | 1.000 | 0.445 |
| VOI15 | 0.001 | 0.001 | 0.001 | 0.917 | 0.708 | 0.002 | 0.445 | 1.000 |

*VOI, volume of interest

**Supplementary Table S4.** Difference of AUC between VOI_I model and VOI_P models in the internal testing set.

| | *VOI_P1 | VOI_P2 | VOI_P3 | VOI_P4 | VOI_P5 | VOI_P10 | VOI_P15 | VOI_I |
|---|---|---|---|---|---|---|---|---|
| VOI_P1 | 1.000 | 0.663 | 0.610 | 0.289 | 0.103 | 0.938 | 0.654 | 0.953 |
| VOI_P2 | 0.663 | 1.000 | 0.950 | 0.769 | 0.548 | 0.721 | 0.489 | 0.776 |
| VOI_P3 | 0.610 | 0.950 | 1.000 | 0.636 | 0.255 | 0.710 | 0.412 | 0.792 |
| VOI_P4 | 0.289 | 0.769 | 0.636 | 1.000 | 0.631 | 0.482 | 0.234 | 0.529 |
| VOI_P5 | 0.103 | 0.548 | 0.255 | 0.631 | 1.000 | 0.292 | 0.068 | 0.375 |
| VOI_P10 | 0.938 | 0.721 | 0.710 | 0.482 | 0.292 | 1.000 | 0.712 | 0.918 |
| VOI_P15 | 0.654 | 0.489 | 0.412 | 0.234 | 0.068 | 0.712 | 1.000 | 0.693 |
| VOI1_I | 0.953 | 0.776 | 0.792 | 0.529 | 0.375 | 0.918 | 0.693 | 1.000 |

*VOI, volume of interest

**Supplementary Table S5.** Difference of AUC between VOI_I model and VOI_P models in the external testing set.

| | *VOI_P1 | VOI_P2 | VOI_P3 | VOI_P4 | VOI_P5 | VOI_P10 | VOI_P15 | VOI_I |
|---|---|---|---|---|---|---|---|---|
| VOI_P1 | 1.000 | 0.521 | 0.191 | 0.571 | 0.239 | 0.276 | 0.323 | 0.968 |
| VOI_P2 | 0.521 | 1.000 | 0.098 | 0.255 | 0.140 | 0.162 | 0.689 | 0.547 |
| VOI_P3 | 0.191 | 0.098 | 1.000 | 0.430 | 0.931 | 0.932 | 0.077 | 0.381 |
| VOI_P4 | 0.571 | 0.255 | 0.430 | 1.000 | 0.503 | 0.490 | 0.142 | 0.731 |
| VOI_P5 | 0.239 | 0.140 | 0.931 | 0.503 | 1.000 | 0.847 | 0.048 | 0.398 |
| VOI_P10 | 0.276 | 0.162 | 0.932 | 0.490 | 0.847 | 1.000 | 0.021 | 0.363 |
| VOI_P15 | 0.323 | 0.689 | 0.077 | 0.142 | 0.048 | 0.021 | 1.000 | 0.346 |
| VOI1_I | 0.968 | 0.547 | 0.381 | 0.731 | 0.398 | 0.363 | 0.346 | 1.000 |

*VOI, volume of interest

**Supplementary Table S6.** 1454 radiomic features extracted from each VOI, including 288 first-order features, 14 shape features, and 1152 texture features.

| | |
|---|---|
| **Shape features** | |
| shape_Elongation_original | shape_Flatness_original |
| shape_LeastAxisLength_original | shape_MajorAxisLength_original |
| shape_Maximum2DDiameterColumn_original | shape_Maximum2DDiameterRow_original |
| shape_Maximum2DDiameterSlice_original | shape_Maximum3DDiameter_original |
| shape_MeshVolume_original | shape_MinorAxisLength_original |
| shape_Sphericity_original | shape_SurfaceArea_original |
| shape_SurfaceVolumeRatio_original | shape_VoxelVolume_original |
| **First-order features** | |
| firstorder_10Percentile_original | firstorder_90Percentile_original |
| firstorder_Energy_original | firstorder_Entropy_original |
| firstorder_InterquartileRange_original | firstorder_Kurtosis_original |
| firstorder_Maximum_original | firstorder_MeanAbsoluteDeviation_original |
| firstorder_Mean_original | firstorder_Median_original |
| firstorder_Minimum_original | firstorder_Range_original |
| firstorder_RobustMeanAbsoluteDeviation_original | firstorder_RootMeanSquared_original |
| firstorder_Skewness_original | firstorder_TotalEnergy_original |
| firstorder_Uniformity_original | firstorder_Variance_original |
| firstorder_10Percentile_log-sigma-1-0-mm-3D | firstorder_90Percentile_log-sigma-1-0-mm-3D |
| firstorder_Energy_log-sigma-1-0-mm-3D | firstorder_Entropy_log-sigma-1-0-mm-3D |
| firstorder_InterquartileRange_log-sigma-1-0-mm-3D | firstorder_Kurtosis_log-sigma-1-0-mm-3D |
| firstorder_Maximum_log-sigma-1-0-mm-3D | firstorder_MeanAbsoluteDeviation_log-sigma-1-0-mm-3D |
| firstorder_Mean_log-sigma-1-0-mm-3D | firstorder_Median_log-sigma-1-0-mm-3D |
| firstorder_Minimum_log-sigma-1-0-mm-3D | firstorder_Range_log-sigma-1-0-mm-3D |
| firstorder_RobustMeanAbsoluteDeviation_log-sigma-1-0-mm-3D | firstorder_RootMeanSquared_log-sigma-1-0-mm-3D |
| firstorder_Skewness_log-sigma-1-0-mm-3D | firstorder_TotalEnergy_log-sigma-1-0-mm-3D |
| firstorder_Uniformity_log-sigma-1-0-mm-3D | firstorder_Variance_log-sigma-1-0-mm-3D |
| firstorder_10Percentile_log-sigma-3-0-mm-3D | firstorder_90Percentile_log-sigma-3-0-mm-3D |
| firstorder_Energy_log-sigma-3-0-mm-3D | firstorder_Entropy_log-sigma-3-0-mm-3D |
| firstorder_InterquartileRange_log-sigma-3-0-mm-3D | firstorder_Kurtosis_log-sigma-3-0-mm-3D |
| firstorder_Maximum_log-sigma-3-0-mm-3D | firstorder_MeanAbsoluteDeviation_log-sigma-3-0-mm-3D |
| firstorder_Mean_log-sigma-3-0-mm-3D | firstorder_Median_log-sigma-3-0-mm-3D |
| firstorder_Minimum_log-sigma-3-0-mm-3D | firstorder_Range_log-sigma-3-0-mm-3D |
| firstorder_RobustMeanAbsoluteDeviation_log-sigma-3-0-mm-3D | firstorder_RootMeanSquared_log-sigma-3-0-mm-3D |
| firstorder_Skewness_log-sigma-3-0-mm-3D | firstorder_TotalEnergy_log-sigma-3-0-mm-3D |
| firstorder_Uniformity_log-sigma-3-0-mm-3D | firstorder_Variance_log-sigma-3-0-mm-3D |
| firstorder_10Percentile_log-sigma-2-0-mm-3D | firstorder_90Percentile_log-sigma-2-0-mm-3D |
| firstorder_Energy_log-sigma-2-0-mm-3D | firstorder_Entropy_log-sigma-2-0-mm-3D |
| firstorder_InterquartileRange_log-sigma-2-0-mm-3D | firstorder_Kurtosis_log-sigma-2-0-mm-3D |
| firstorder_Maximum_log-sigma-2-0-mm-3D | firstorder_MeanAbsoluteDeviation_log-sigma-2-0-mm-3D |

| | |
|---|---|
| firstorder_Mean_log-sigma-2-0-mm-3D | firstorder_Median_log-sigma-2-0-mm-3D |
| firstorder_Minimum_log-sigma-2-0-mm-3D | firstorder_Range_log-sigma-2-0-mm-3D |
| firstorder_RobustMeanAbsoluteDeviation_log-sigma-2-0-mm-3D | firstorder_RootMeanSquared_log-sigma-2-0-mm-3D |
| firstorder_Skewness_log-sigma-2-0-mm-3D | firstorder_TotalEnergy_log-sigma-2-0-mm-3D |
| firstorder_Uniformity_log-sigma-2-0-mm-3D | firstorder_Variance_log-sigma-2-0-mm-3D |
| firstorder_10Percentile_wavelet-LLH | firstorder_90Percentile_wavelet-LLH |
| firstorder_Energy_wavelet-LLH | firstorder_Entropy_wavelet-LLH |
| firstorder_InterquartileRange_wavelet-LLH | firstorder_Kurtosis_wavelet-LLH |
| firstorder_Maximum_wavelet-LLH | firstorder_MeanAbsoluteDeviation_wavelet-LLH |
| firstorder_Mean_wavelet-LLH | firstorder_Median_wavelet-LLH |
| firstorder_Minimum_wavelet-LLH | firstorder_Range_wavelet-LLH |
| firstorder_RobustMeanAbsoluteDeviation_wavelet-LLH | firstorder_RootMeanSquared_wavelet-LLH |
| firstorder_Skewness_wavelet-LLH | firstorder_TotalEnergy_wavelet-LLH |
| firstorder_Uniformity_wavelet-LLH | firstorder_Variance_wavelet-LLH |
| firstorder_10Percentile_wavelet-HLL | firstorder_90Percentile_wavelet-HLL |
| firstorder_Energy_wavelet-HLL | firstorder_Entropy_wavelet-HLL |
| firstorder_InterquartileRange_wavelet-HLL | firstorder_Kurtosis_wavelet-HLL |
| firstorder_Maximum_wavelet-HLL | firstorder_MeanAbsoluteDeviation_wavelet-HLL |
| firstorder_Mean_wavelet-HLL | firstorder_Median_wavelet-HLL |
| firstorder_Minimum_wavelet-HLL | firstorder_Range_wavelet-HLL |
| firstorder_RobustMeanAbsoluteDeviation_wavelet-HLL | firstorder_RootMeanSquared_wavelet-HLL |
| firstorder_Skewness_wavelet-HLL | firstorder_TotalEnergy_wavelet-HLL |
| firstorder_Uniformity_wavelet-HLL | firstorder_Variance_wavelet-HLL |
| firstorder_10Percentile_wavelet-LHL | firstorder_90Percentile_wavelet-LHL |
| firstorder_Energy_wavelet-LHL | firstorder_Entropy_wavelet-LHL |
| firstorder_InterquartileRange_wavelet-LHL | firstorder_Kurtosis_wavelet-LHL |
| firstorder_Maximum_wavelet-LHL | firstorder_MeanAbsoluteDeviation_wavelet-LHL |
| firstorder_Mean_wavelet-LHL | firstorder_Median_wavelet-LHL |
| firstorder_Minimum_wavelet-LHL | firstorder_Range_wavelet-LHL |
| firstorder_RobustMeanAbsoluteDeviation_wavelet-LHL | firstorder_RootMeanSquared_wavelet-LHL |
| firstorder_Skewness_wavelet-LHL | firstorder_TotalEnergy_wavelet-LHL |
| firstorder_Uniformity_wavelet-LHL | firstorder_Variance_wavelet-LHL |
| firstorder_10Percentile_wavelet-LHH | firstorder_90Percentile_wavelet-LHH |
| firstorder_Energy_wavelet-LHH | firstorder_Entropy_wavelet-LHH |
| firstorder_InterquartileRange_wavelet-LHH | firstorder_Kurtosis_wavelet-LHH |
| firstorder_Maximum_wavelet-LHH | firstorder_MeanAbsoluteDeviation_wavelet-LHH |
| firstorder_Mean_wavelet-LHH | firstorder_Median_wavelet-LHH |
| firstorder_Minimum_wavelet-LHH | firstorder_Range_wavelet-LHH |
| firstorder_RobustMeanAbsoluteDeviation_wavelet-LHH | firstorder_RootMeanSquared_wavelet-LHH |
| firstorder_Skewness_wavelet-LHH | firstorder_TotalEnergy_wavelet-LHH |
| firstorder_Uniformity_wavelet-LHH | firstorder_Variance_wavelet-LHH |
| firstorder_10Percentile_wavelet-HLH | firstorder_90Percentile_wavelet-HLH |
| firstorder_Energy_wavelet-HLH | firstorder_Entropy_wavelet-HLH |

| | |
|---|---|
| firstorder_InterquartileRange_wavelet-HLH | firstorder_Kurtosis_wavelet-HLH |
| firstorder_Maximum_wavelet-HLH | firstorder_MeanAbsoluteDeviation_wavelet-HLH |
| firstorder_Mean_wavelet-HLH | firstorder_Median_wavelet-HLH |
| firstorder_Minimum_wavelet-HLH | firstorder_Range_wavelet-HLH |
| firstorder_RobustMeanAbsoluteDeviation_wavelet-HLH | firstorder_RootMeanSquared_wavelet-HLH |
| firstorder_Skewness_wavelet-HLH | firstorder_TotalEnergy_wavelet-HLH |
| firstorder_Uniformity_wavelet-HLH | firstorder_Variance_wavelet-HLH |
| firstorder_10Percentile_wavelet-HHL | firstorder_90Percentile_wavelet-HHL |
| firstorder_Energy_wavelet-HHL | firstorder_Entropy_wavelet-HHL |
| firstorder_InterquartileRange_wavelet-HHL | firstorder_Kurtosis_wavelet-HHL |
| firstorder_Maximum_wavelet-HHL | firstorder_MeanAbsoluteDeviation_wavelet-HHL |
| firstorder_Mean_wavelet-HHL | firstorder_Median_wavelet-HHL |
| firstorder_Minimum_wavelet-HHL | firstorder_Range_wavelet-HHL |
| firstorder_RobustMeanAbsoluteDeviation_wavelet-HHL | firstorder_RootMeanSquared_wavelet-HHL |
| firstorder_Skewness_wavelet-HHL | firstorder_TotalEnergy_wavelet-HHL |
| firstorder_Uniformity_wavelet-HHL | firstorder_Variance_wavelet-HHL |
| firstorder_10Percentile_wavelet-HHH | firstorder_90Percentile_wavelet-HHH |
| firstorder_Energy_wavelet-HHH | firstorder_Entropy_wavelet-HHH |
| firstorder_InterquartileRange_wavelet-HHH | firstorder_Kurtosis_wavelet-HHH |
| firstorder_Maximum_wavelet-HHH | firstorder_MeanAbsoluteDeviation_wavelet-HHH |
| firstorder_Mean_wavelet-HHH | firstorder_Median_wavelet-HHH |
| firstorder_Minimum_wavelet-HHH | firstorder_Range_wavelet-HHH |
| firstorder_RobustMeanAbsoluteDeviation_wavelet-HHH | firstorder_RootMeanSquared_wavelet-HHH |
| firstorder_Skewness_wavelet-HHH | firstorder_TotalEnergy_wavelet-HHH |
| firstorder_Uniformity_wavelet-HHH | firstorder_Variance_wavelet-HHH |
| firstorder_10Percentile_square | firstorder_90Percentile_square |
| firstorder_Energy_square | firstorder_Entropy_square |
| firstorder_InterquartileRange_square | firstorder_Kurtosis_square |
| firstorder_Maximum_square | firstorder_MeanAbsoluteDeviation_square |
| firstorder_Mean_square | firstorder_Median_square |
| firstorder_Minimum_square | firstorder_Range_square |
| firstorder_RobustMeanAbsoluteDeviation_square | firstorder_RootMeanSquared_square |
| firstorder_Skewness_square | firstorder_TotalEnergy_square |
| firstorder_Uniformity_square | firstorder_Variance_square |
| firstorder_10Percentile_squareroot | firstorder_90Percentile_squareroot |
| firstorder_Energy_squareroot | firstorder_Entropy_squareroot |
| firstorder_InterquartileRange_squareroot | firstorder_Kurtosis_squareroot |
| firstorder_Maximum_squareroot | firstorder_MeanAbsoluteDeviation_squareroot |
| firstorder_Mean_squareroot | firstorder_Median_squareroot |
| firstorder_Minimum_squareroot | firstorder_Range_squareroot |
| firstorder_RobustMeanAbsoluteDeviation_squareroot | firstorder_RootMeanSquared_squareroot |
| firstorder_Skewness_squareroot | firstorder_TotalEnergy_squareroot |
| firstorder_Uniformity_squareroot | firstorder_Variance_squareroot |

| | |
|---|---|
| firstorder_10Percentile_logarithm | firstorder_90Percentile_logarithm |
| firstorder_Energy_logarithm | firstorder_Entropy_logarithm |
| firstorder_InterquartileRange_logarithm | firstorder_Kurtosis_logarithm |
| firstorder_Maximum_logarithm | firstorder_MeanAbsoluteDeviation_logarithm |
| firstorder_Mean_logarithm | firstorder_Median_logarithm |
| firstorder_Minimum_logarithm | firstorder_Range_logarithm |
| firstorder_RobustMeanAbsoluteDeviation_logarithm | firstorder_RootMeanSquared_logarithm |
| firstorder_Skewness_logarithm | firstorder_TotalEnergy_logarithm |
| firstorder_Uniformity_logarithm | firstorder_Variance_logarithm |
| **Texture features** | |
| glcm_Autocorrelation_original | glcm_JointAverage_original |
| glcm_ClusterProminence_original | glcm_ClusterShade_original |
| glcm_ClusterTendency_original | glcm_Contrast_original |
| glcm_Correlation_original | glcm_DifferenceAverage_original |
| glcm_DifferenceEntropy_original | glcm_DifferenceVariance_original |
| glcm_JointEnergy_original | glcm_JointEntropy_original |
| glcm_Imc1_original | glcm_Imc2_original |
| glcm_Idm_original | glcm_Idmn_original |
| glcm_Id_original | glcm_Idn_original |
| glcm_InverseVariance_original | glcm_MaximumProbability_original |
| glcm_SumEntropy_original | gldm_DependenceEntropy_original |
| gldm_DependenceNonUniformity_original | gldm_DependenceNonUniformityNormalized_original |
| gldm_DependenceVariance_original | gldm_GrayLevelNonUniformity_original |
| gldm_GrayLevelVariance_original | gldm_HighGrayLevelEmphasis_original |
| gldm_LargeDependenceEmphasis_original | gldm_LargeDependenceHighGrayLevelEmphasis_original |
| gldm_LargeDependenceLowGrayLevelEmphasis_original | gldm_LowGrayLevelEmphasis_original |
| gldm_SmallDependenceEmphasis_original | gldm_SmallDependenceHighGrayLevelEmphasis_original |
| gldm_SmallDependenceLowGrayLevelEmphasis_original | glrlm_GrayLevelNonUniformity_original |
| glrlm_GrayLevelNonUniformityNormalized_original | glrlm_GrayLevelVariance_original |
| glrlm_HighGrayLevelRunEmphasis_original | glrlm_LongRunEmphasis_original |
| glrlm_LongRunHighGrayLevelEmphasis_original | glrlm_LongRunLowGrayLevelEmphasis_original |
| glrlm_LowGrayLevelRunEmphasis_original | glrlm_RunEntropy_original |
| glrlm_RunLengthNonUniformity_original | glrlm_RunLengthNonUniformityNormalized_original |
| glrlm_RunPercentage_original | glrlm_RunVariance_original |
| glrlm_ShortRunEmphasis_original | glrlm_ShortRunHighGrayLevelEmphasis_original |
| glrlm_ShortRunLowGrayLevelEmphasis_original | glszm_GrayLevelNonUniformity_original |
| glszm_GrayLevelNonUniformityNormalized_original | glszm_GrayLevelVariance_original |
| glszm_HighGrayLevelZoneEmphasis_original | glszm_LargeAreaEmphasis_original |
| glszm_LargeAreaHighGrayLevelEmphasis_original | glszm_LargeAreaLowGrayLevelEmphasis_original |
| glszm_LowGrayLevelZoneEmphasis_original | glszm_SizeZoneNonUniformity_original |
| glszm_SizeZoneNonUniformityNormalized_original | glszm_SmallAreaEmphasis_original |
| glszm_SmallAreaHighGrayLevelEmphasis_original | glszm_SmallAreaLowGrayLevelEmphasis_original |
| glszm_ZoneEntropy_original | glszm_ZonePercentage_original |

| | |
|---|---|
| glszm_ZoneVariance_original | ngtdm_Busyness_original |
| ngtdm_Coarseness_original | ngtdm_Complexity_original |
| ngtdm_Contrast_original | ngtdm_Strength_original |
| glcm_Autocorrelation_log-sigma-1-0-mm-3D | glcm_JointAverage_log-sigma-1-0-mm-3D |
| glcm_ClusterProminence_log-sigma-1-0-mm-3D | glcm_ClusterShade_log-sigma-1-0-mm-3D |
| glcm_ClusterTendency_log-sigma-1-0-mm-3D | glcm_Contrast_log-sigma-1-0-mm-3D |
| glcm_Correlation_log-sigma-1-0-mm-3D | glcm_DifferenceAverage_log-sigma-1-0-mm-3D |
| glcm_DifferenceEntropy_log-sigma-1-0-mm-3D | glcm_DifferenceVariance_log-sigma-1-0-mm-3D |
| glcm_JointEnergy_log-sigma-1-0-mm-3D | glcm_JointEntropy_log-sigma-1-0-mm-3D |
| glcm_Imc1_log-sigma-1-0-mm-3D | glcm_Imc2_log-sigma-1-0-mm-3D |
| glcm_Idm_log-sigma-1-0-mm-3D | glcm_Idmn_log-sigma-1-0-mm-3D |
| glcm_Id_log-sigma-1-0-mm-3D | glcm_Idn_log-sigma-1-0-mm-3D |
| glcm_InverseVariance_log-sigma-1-0-mm-3D | glcm_MaximumProbability_log-sigma-1-0-mm-3D |
| glcm_SumEntropy_log-sigma-1-0-mm-3D | gldm_DependenceEntropy_log-sigma-1-0-mm-3D |
| gldm_DependenceNonUniformity_log-sigma-1-0-mm-3D | gldm_DependenceNonUniformityNormalized_log-sigma-1-0-mm-3D |
| gldm_DependenceVariance_log-sigma-1-0-mm-3D | gldm_GrayLevelNonUniformity_log-sigma-1-0-mm-3D |
| gldm_GrayLevelVariance_log-sigma-1-0-mm-3D | gldm_HighGrayLevelEmphasis_log-sigma-1-0-mm-3D |
| gldm_LargeDependenceEmphasis_log-sigma-1-0-mm-3D | gldm_LargeDependenceHighGrayLevelEmphasis_log-sigma-1-0-mm-3D |
| gldm_LargeDependenceLowGrayLevelEmphasis_log-sigma-1-0-mm-3D | gldm_LowGrayLevelEmphasis_log-sigma-1-0-mm-3D |
| gldm_SmallDependenceEmphasis_log-sigma-1-0-mm-3D | gldm_SmallDependenceHighGrayLevelEmphasis_log-sigma-1-0-mm-3D |
| gldm_SmallDependenceLowGrayLevelEmphasis_log-sigma-1-0-mm-3D | glrlm_GrayLevelNonUniformity_log-sigma-1-0-mm-3D |
| glrlm_GrayLevelNonUniformityNormalized_log-sigma-1-0-mm-3D | glrlm_GrayLevelVariance_log-sigma-1-0-mm-3D |
| glrlm_HighGrayLevelRunEmphasis_log-sigma-1-0-mm-3D | glrlm_LongRunEmphasis_log-sigma-1-0-mm-3D |
| glrlm_LongRunHighGrayLevelEmphasis_log-sigma-1-0-mm-3D | glrlm_LongRunLowGrayLevelEmphasis_log-sigma-1-0-mm-3D |
| glrlm_LowGrayLevelRunEmphasis_log-sigma-1-0-mm-3D | glrlm_RunEntropy_log-sigma-1-0-mm-3D |
| glrlm_RunLengthNonUniformity_log-sigma-1-0-mm-3D | glrlm_RunLengthNonUniformityNormalized_log-sigma-1-0-mm-3D |
| glrlm_RunPercentage_log-sigma-1-0-mm-3D | glrlm_RunVariance_log-sigma-1-0-mm-3D |
| glrlm_ShortRunEmphasis_log-sigma-1-0-mm-3D | glrlm_ShortRunHighGrayLevelEmphasis_log-sigma-1-0-mm-3D |
| glrlm_ShortRunLowGrayLevelEmphasis_log-sigma-1-0-mm-3D | glszm_GrayLevelNonUniformity_log-sigma-1-0-mm-3D |
| glszm_GrayLevelNonUniformityNormalized_log-sigma-1-0-mm-3D | glszm_GrayLevelVariance_log-sigma-1-0-mm-3D |
| glszm_HighGrayLevelZoneEmphasis_log-sigma-1-0-mm-3D | glszm_LargeAreaEmphasis_log-sigma-1-0-mm-3D |
| glszm_LargeAreaHighGrayLevelEmphasis_log-sigma-1-0-mm-3D | glszm_LargeAreaLowGrayLevelEmphasis_log-sigma-1-0-mm-3D |
| glszm_LowGrayLevelZoneEmphasis_log-sigma-1-0-mm-3D | glszm_SizeZoneNonUniformity_log-sigma-1-0-mm-3D |
| glszm_SizeZoneNonUniformityNormalized_log-sigma-1-0-mm-3D | glszm_SmallAreaEmphasis_log-sigma-1-0-mm-3D |
| glszm_SmallAreaHighGrayLevelEmphasis_log-sigma-1-0-mm-3D | glszm_SmallAreaLowGrayLevelEmphasis_log-sigma-1-0-mm-3D |
| glszm_ZoneEntropy_log-sigma-1-0-mm-3D | glszm_ZonePercentage_log-sigma-1-0-mm-3D |
| glszm_ZoneVariance_log-sigma-1-0-mm-3D | ngtdm_Busyness_log-sigma-1-0-mm-3D |
| ngtdm_Coarseness_log-sigma-1-0-mm-3D | ngtdm_Complexity_log-sigma-1-0-mm-3D |
| ngtdm_Contrast_log-sigma-1-0-mm-3D | ngtdm_Strength_log-sigma-1-0-mm-3D |
| glcm_Autocorrelation_log-sigma-2-0-mm-3D | glcm_JointAverage_log-sigma-2-0-mm-3D |
| glcm_ClusterProminence_log-sigma-2-0-mm-3D | glcm_ClusterShade_log-sigma-2-0-mm-3D |
| glcm_ClusterTendency_log-sigma-2-0-mm-3D | glcm_Contrast_log-sigma-2-0-mm-3D |
| glcm_Correlation_log-sigma-2-0-mm-3D | glcm_DifferenceAverage_log-sigma-2-0-mm-3D |

| | |
|---|---|
| glcm_DifferenceEntropy_log-sigma-2-0-mm-3D | glcm_DifferenceVariance_log-sigma-2-0-mm-3D |
| glcm_JointEnergy_log-sigma-2-0-mm-3D | glcm_JointEntropy_log-sigma-2-0-mm-3D |
| glcm_Imc1_log-sigma-2-0-mm-3D | glcm_Imc2_log-sigma-2-0-mm-3D |
| glcm_Idm_log-sigma-2-0-mm-3D | glcm_Idmn_log-sigma-2-0-mm-3D |
| glcm_Id_log-sigma-2-0-mm-3D | glcm_Idn_log-sigma-2-0-mm-3D |
| glcm_InverseVariance_log-sigma-2-0-mm-3D | glcm_MaximumProbability_log-sigma-2-0-mm-3D |
| glcm_SumEntropy_log-sigma-2-0-mm-3D | gldm_DependenceEntropy_log-sigma-2-0-mm-3D |
| gldm_DependenceNonUniformity_log-sigma-2-0-mm-3D | gldm_DependenceNonUniformityNormalized_log-sigma-2-0-mm-3D |
| gldm_DependenceVariance_log-sigma-2-0-mm-3D | gldm_GrayLevelNonUniformity_log-sigma-2-0-mm-3D |
| gldm_GrayLevelVariance_log-sigma-2-0-mm-3D | gldm_HighGrayLevelEmphasis_log-sigma-2-0-mm-3D |
| gldm_LargeDependenceEmphasis_log-sigma-2-0-mm-3D | gldm_LargeDependenceHighGrayLevelEmphasis_log-sigma-2-0-mm-3D |
| gldm_LargeDependenceLowGrayLevelEmphasis_log-sigma-2-0-mm-3D | gldm_LowGrayLevelEmphasis_log-sigma-2-0-mm-3D |
| gldm_SmallDependenceEmphasis_log-sigma-2-0-mm-3D | gldm_SmallDependenceHighGrayLevelEmphasis_log-sigma-2-0-mm-3D |
| gldm_SmallDependenceLowGrayLevelEmphasis_log-sigma-2-0-mm-3D | glrlm_GrayLevelNonUniformity_log-sigma-2-0-mm-3D |
| glrlm_GrayLevelNonUniformityNormalized_log-sigma-2-0-mm-3D | glrlm_GrayLevelVariance_log-sigma-2-0-mm-3D |
| glrlm_HighGrayLevelRunEmphasis_log-sigma-2-0-mm-3D | glrlm_LongRunEmphasis_log-sigma-2-0-mm-3D |
| glrlm_LongRunHighGrayLevelEmphasis_log-sigma-2-0-mm-3D | glrlm_LongRunLowGrayLevelEmphasis_log-sigma-2-0-mm-3D |
| glrlm_LowGrayLevelRunEmphasis_log-sigma-2-0-mm-3D | glrlm_RunEntropy_log-sigma-2-0-mm-3D |
| glrlm_RunLengthNonUniformity_log-sigma-2-0-mm-3D | glrlm_RunLengthNonUniformityNormalized_log-sigma-2-0-mm-3D |
| glrlm_RunPercentage_log-sigma-2-0-mm-3D | glrlm_RunVariance_log-sigma-2-0-mm-3D |
| glrlm_ShortRunEmphasis_log-sigma-2-0-mm-3D | glrlm_ShortRunHighGrayLevelEmphasis_log-sigma-2-0-mm-3D |
| glrlm_ShortRunLowGrayLevelEmphasis_log-sigma-2-0-mm-3D | glszm_GrayLevelNonUniformity_log-sigma-2-0-mm-3D |
| glszm_GrayLevelNonUniformityNormalized_log-sigma-2-0-mm-3D | glszm_GrayLevelVariance_log-sigma-2-0-mm-3D |
| glszm_HighGrayLevelZoneEmphasis_log-sigma-2-0-mm-3D | glszm_LargeAreaEmphasis_log-sigma-2-0-mm-3D |
| glszm_LargeAreaHighGrayLevelEmphasis_log-sigma-2-0-mm-3D | glszm_LargeAreaLowGrayLevelEmphasis_log-sigma-2-0-mm-3D |
| glszm_LowGrayLevelZoneEmphasis_log-sigma-2-0-mm-3D | glszm_SizeZoneNonUniformity_log-sigma-2-0-mm-3D |
| glszm_SizeZoneNonUniformityNormalized_log-sigma-2-0-mm-3D | glszm_SmallAreaEmphasis_log-sigma-2-0-mm-3D |
| glszm_SmallAreaHighGrayLevelEmphasis_log-sigma-2-0-mm-3D | glszm_SmallAreaLowGrayLevelEmphasis_log-sigma-2-0-mm-3D |
| glszm_ZoneEntropy_log-sigma-2-0-mm-3D | glszm_ZonePercentage_log-sigma-2-0-mm-3D |
| glszm_ZoneVariance_log-sigma-2-0-mm-3D | ngtdm_Busyness_log-sigma-2-0-mm-3D |
| ngtdm_Coarseness_log-sigma-2-0-mm-3D | ngtdm_Complexity_log-sigma-2-0-mm-3D |
| ngtdm_Contrast_log-sigma-2-0-mm-3D | ngtdm_Strength_log-sigma-2-0-mm-3D |
| glcm_Autocorrelation_log-sigma-3-0-mm-3D | glcm_JointAverage_log-sigma-3-0-mm-3D |
| glcm_ClusterProminence_log-sigma-3-0-mm-3D | glcm_ClusterShade_log-sigma-3-0-mm-3D |
| glcm_ClusterTendency_log-sigma-3-0-mm-3D | glcm_Contrast_log-sigma-3-0-mm-3D |
| glcm_Correlation_log-sigma-3-0-mm-3D | glcm_DifferenceAverage_log-sigma-3-0-mm-3D |
| glcm_DifferenceEntropy_log-sigma-3-0-mm-3D | glcm_DifferenceVariance_log-sigma-3-0-mm-3D |
| glcm_JointEnergy_log-sigma-3-0-mm-3D | glcm_JointEntropy_log-sigma-3-0-mm-3D |
| glcm_Imc1_log-sigma-3-0-mm-3D | glcm_Imc2_log-sigma-3-0-mm-3D |
| glcm_Idm_log-sigma-3-0-mm-3D | glcm_Idmn_log-sigma-3-0-mm-3D |
| glcm_Id_log-sigma-3-0-mm-3D | glcm_Idn_log-sigma-3-0-mm-3D |
| glcm_InverseVariance_log-sigma-3-0-mm-3D | glcm_MaximumProbability_log-sigma-3-0-mm-3D |
| glcm_SumEntropy_log-sigma-3-0-mm-3D | gldm_DependenceEntropy_log-sigma-3-0-mm-3D |

| | |
|---|---|
| gldm_DependenceNonUniformity_log-sigma-3-0-mm-3D | gldm_DependenceNonUniformityNormalized_log-sigma-3-0-mm-3D |
| gldm_DependenceVariance_log-sigma-3-0-mm-3D | gldm_GrayLevelNonUniformity_log-sigma-3-0-mm-3D |
| gldm_GrayLevelVariance_log-sigma-3-0-mm-3D | gldm_HighGrayLevelEmphasis_log-sigma-3-0-mm-3D |
| gldm_LargeDependenceEmphasis_log-sigma-3-0-mm-3D | gldm_LargeDependenceHighGrayLevelEmphasis_log-sigma-3-0-mm-3D |
| gldm_LargeDependenceLowGrayLevelEmphasis_log-sigma-3-0-mm-3D | gldm_LowGrayLevelEmphasis_log-sigma-3-0-mm-3D |
| gldm_SmallDependenceEmphasis_log-sigma-3-0-mm-3D | gldm_SmallDependenceHighGrayLevelEmphasis_log-sigma-3-0-mm-3D |
| gldm_SmallDependenceLowGrayLevelEmphasis_log-sigma-3-0-mm-3D | glrlm_GrayLevelNonUniformity_log-sigma-3-0-mm-3D |
| glrlm_GrayLevelNonUniformityNormalized_log-sigma-3-0-mm-3D | glrlm_GrayLevelVariance_log-sigma-3-0-mm-3D |
| glrlm_HighGrayLevelRunEmphasis_log-sigma-3-0-mm-3D | glrlm_LongRunEmphasis_log-sigma-3-0-mm-3D |
| glrlm_LongRunHighGrayLevelEmphasis_log-sigma-3-0-mm-3D | glrlm_LongRunLowGrayLevelEmphasis_log-sigma-3-0-mm-3D |
| glrlm_LowGrayLevelRunEmphasis_log-sigma-3-0-mm-3D | glrlm_RunEntropy_log-sigma-3-0-mm-3D |
| glrlm_RunLengthNonUniformity_log-sigma-3-0-mm-3D | glrlm_RunLengthNonUniformityNormalized_log-sigma-3-0-mm-3D |
| glrlm_RunPercentage_log-sigma-3-0-mm-3D | glrlm_RunVariance_log-sigma-3-0-mm-3D |
| glrlm_ShortRunEmphasis_log-sigma-3-0-mm-3D | glrlm_ShortRunHighGrayLevelEmphasis_log-sigma-3-0-mm-3D |
| glrlm_ShortRunLowGrayLevelEmphasis_log-sigma-3-0-mm-3D | glszm_GrayLevelNonUniformity_log-sigma-3-0-mm-3D |
| glszm_GrayLevelNonUniformityNormalized_log-sigma-3-0-mm-3D | glszm_GrayLevelVariance_log-sigma-3-0-mm-3D |
| glszm_HighGrayLevelZoneEmphasis_log-sigma-3-0-mm-3D | glszm_LargeAreaEmphasis_log-sigma-3-0-mm-3D |
| glszm_LargeAreaHighGrayLevelEmphasis_log-sigma-3-0-mm-3D | glszm_LargeAreaLowGrayLevelEmphasis_log-sigma-3-0-mm-3D |
| glszm_LowGrayLevelZoneEmphasis_log-sigma-3-0-mm-3D | glszm_SizeZoneNonUniformity_log-sigma-3-0-mm-3D |
| glszm_SizeZoneNonUniformityNormalized_log-sigma-3-0-mm-3D | glszm_SmallAreaEmphasis_log-sigma-3-0-mm-3D |
| glszm_SmallAreaHighGrayLevelEmphasis_log-sigma-3-0-mm-3D | glszm_SmallAreaLowGrayLevelEmphasis_log-sigma-3-0-mm-3D |
| glszm_ZoneEntropy_log-sigma-3-0-mm-3D | glszm_ZonePercentage_log-sigma-3-0-mm-3D |
| glszm_ZoneVariance_log-sigma-3-0-mm-3D | ngtdm_Busyness_log-sigma-3-0-mm-3D |
| ngtdm_Coarseness_log-sigma-3-0-mm-3D | ngtdm_Complexity_log-sigma-3-0-mm-3D |
| ngtdm_Contrast_log-sigma-3-0-mm-3D | ngtdm_Strength_log-sigma-3-0-mm-3D |
| glcm_Autocorrelation_wavelet-LLH | glcm_JointAverage_wavelet-LLH |
| glcm_ClusterProminence_wavelet-LLH | glcm_ClusterShade_wavelet-LLH |
| glcm_ClusterTendency_wavelet-LLH | glcm_Contrast_wavelet-LLH |
| glcm_Correlation_wavelet-LLH | glcm_DifferenceAverage_wavelet-LLH |
| glcm_DifferenceEntropy_wavelet-LLH | glcm_DifferenceVariance_wavelet-LLH |
| glcm_JointEnergy_wavelet-LLH | glcm_JointEntropy_wavelet-LLH |
| glcm_Imc1_wavelet-LLH | glcm_Imc2_wavelet-LLH |
| glcm_Idm_wavelet-LLH | glcm_Idmn_wavelet-LLH |
| glcm_Id_wavelet-LLH | glcm_Idn_wavelet-LLH |
| glcm_InverseVariance_wavelet-LLH | glcm_MaximumProbability_wavelet-LLH |
| glcm_SumEntropy_wavelet-LLH | gldm_DependenceEntropy_wavelet-LLH |
| gldm_DependenceNonUniformity_wavelet-LLH | gldm_DependenceNonUniformityNormalized_wavelet-LLH |
| gldm_DependenceVariance_wavelet-LLH | gldm_GrayLevelNonUniformity_wavelet-LLH |
| gldm_GrayLevelVariance_wavelet-LLH | gldm_HighGrayLevelEmphasis_wavelet-LLH |
| gldm_LargeDependenceEmphasis_wavelet-LLH | gldm_LargeDependenceHighGrayLevelEmphasis_wavelet-LLH |
| gldm_LargeDependenceLowGrayLevelEmphasis_wavelet-LLH | gldm_LowGrayLevelEmphasis_wavelet-LLH |
| gldm_SmallDependenceEmphasis_wavelet-LLH | gldm_SmallDependenceHighGrayLevelEmphasis_wavelet-LLH |
| gldm_SmallDependenceLowGrayLevelEmphasis_wavelet-LLH | glrlm_GrayLevelNonUniformity_wavelet-LLH |

| | |
|---|---|
| glrlm_GrayLevelNonUniformityNormalized_wavelet-LLH | glrlm_GrayLevelVariance_wavelet-LLH |
| glrlm_HighGrayLevelRunEmphasis_wavelet-LLH | glrlm_LongRunEmphasis_wavelet-LLH |
| glrlm_LongRunHighGrayLevelEmphasis_wavelet-LLH | glrlm_LongRunLowGrayLevelEmphasis_wavelet-LLH |
| glrlm_LowGrayLevelRunEmphasis_wavelet-LLH | glrlm_RunEntropy_wavelet-LLH |
| glrlm_RunLengthNonUniformity_wavelet-LLH | glrlm_RunLengthNonUniformityNormalized_wavelet-LLH |
| glrlm_RunPercentage_wavelet-LLH | glrlm_RunVariance_wavelet-LLH |
| glrlm_ShortRunEmphasis_wavelet-LLH | glrlm_ShortRunHighGrayLevelEmphasis_wavelet-LLH |
| glrlm_ShortRunLowGrayLevelEmphasis_wavelet-LLH | glszm_GrayLevelNonUniformity_wavelet-LLH |
| glszm_GrayLevelNonUniformityNormalized_wavelet-LLH | glszm_GrayLevelVariance_wavelet-LLH |
| glszm_HighGrayLevelZoneEmphasis_wavelet-LLH | glszm_LargeAreaEmphasis_wavelet-LLH |
| glszm_LargeAreaHighGrayLevelEmphasis_wavelet-LLH | glszm_LargeAreaLowGrayLevelEmphasis_wavelet-LLH |
| glszm_LowGrayLevelZoneEmphasis_wavelet-LLH | glszm_SizeZoneNonUniformity_wavelet-LLH |
| glszm_SizeZoneNonUniformityNormalized_wavelet-LLH | glszm_SmallAreaEmphasis_wavelet-LLH |
| glszm_SmallAreaHighGrayLevelEmphasis_wavelet-LLH | glszm_SmallAreaLowGrayLevelEmphasis_wavelet-LLH |
| glszm_ZoneEntropy_wavelet-LLH | glszm_ZonePercentage_wavelet-LLH |
| glszm_ZoneVariance_wavelet-LLH | ngtdm_Busyness_wavelet-LLH |
| ngtdm_Coarseness_wavelet-LLH | ngtdm_Complexity_wavelet-LLH |
| ngtdm_Contrast_wavelet-LLH | ngtdm_Strength_wavelet-LLH |
| glcm_Autocorrelation_wavelet-LHL | glcm_JointAverage_wavelet-LHL |
| glcm_ClusterProminence_wavelet-LHL | glcm_ClusterShade_wavelet-LHL |
| glcm_ClusterTendency_wavelet-LHL | glcm_Contrast_wavelet-LHL |
| glcm_Correlation_wavelet-LHL | glcm_DifferenceAverage_wavelet-LHL |
| glcm_DifferenceEntropy_wavelet-LHL | glcm_DifferenceVariance_wavelet-LHL |
| glcm_JointEnergy_wavelet-LHL | glcm_JointEntropy_wavelet-LHL |
| glcm_Imc1_wavelet-LHL | glcm_Imc2_wavelet-LHL |
| glcm_Idm_wavelet-LHL | glcm_Idmn_wavelet-LHL |
| glcm_Id_wavelet-LHL | glcm_Idn_wavelet-LHL |
| glcm_InverseVariance_wavelet-LHL | glcm_MaximumProbability_wavelet-LHL |
| glcm_SumEntropy_wavelet-LHL | gldm_DependenceEntropy_wavelet-LHL |
| gldm_DependenceNonUniformity_wavelet-LHL | gldm_DependenceNonUniformityNormalized_wavelet-LHL |
| gldm_DependenceVariance_wavelet-LHL | gldm_GrayLevelNonUniformity_wavelet-LHL |
| gldm_GrayLevelVariance_wavelet-LHL | gldm_HighGrayLevelEmphasis_wavelet-LHL |
| gldm_LargeDependenceEmphasis_wavelet-LHL | gldm_LargeDependenceHighGrayLevelEmphasis_wavelet-LHL |
| gldm_LargeDependenceLowGrayLevelEmphasis_wavelet-LHL | gldm_LowGrayLevelEmphasis_wavelet-LHL |
| gldm_SmallDependenceEmphasis_wavelet-LHL | gldm_SmallDependenceHighGrayLevelEmphasis_wavelet-LHL |
| gldm_SmallDependenceLowGrayLevelEmphasis_wavelet-LHL | glrlm_GrayLevelNonUniformity_wavelet-LHL |
| glrlm_GrayLevelNonUniformityNormalized_wavelet-LHL | glrlm_GrayLevelVariance_wavelet-LHL |
| glrlm_HighGrayLevelRunEmphasis_wavelet-LHL | glrlm_LongRunEmphasis_wavelet-LHL |
| glrlm_LongRunHighGrayLevelEmphasis_wavelet-LHL | glrlm_LongRunLowGrayLevelEmphasis_wavelet-LHL |
| glrlm_LowGrayLevelRunEmphasis_wavelet-LHL | glrlm_RunEntropy_wavelet-LHL |
| glrlm_RunLengthNonUniformity_wavelet-LHL | glrlm_RunLengthNonUniformityNormalized_wavelet-LHL |
| glrlm_RunPercentage_wavelet-LHL | glrlm_RunVariance_wavelet-LHL |
| glrlm_ShortRunEmphasis_wavelet-LHL | glrlm_ShortRunHighGrayLevelEmphasis_wavelet-LHL |

| | |
|---|---|
| glrlm_ShortRunLowGrayLevelEmphasis_wavelet-LHL | glszm_GrayLevelNonUniformity_wavelet-LHL |
| glszm_GrayLevelNonUniformityNormalized_wavelet-LHL | glszm_GrayLevelVariance_wavelet-LHL |
| glszm_HighGrayLevelZoneEmphasis_wavelet-LHL | glszm_LargeAreaEmphasis_wavelet-LHL |
| glszm_LargeAreaHighGrayLevelEmphasis_wavelet-LHL | glszm_LargeAreaLowGrayLevelEmphasis_wavelet-LHL |
| glszm_LowGrayLevelZoneEmphasis_wavelet-LHL | glszm_SizeZoneNonUniformity_wavelet-LHL |
| glszm_SizeZoneNonUniformityNormalized_wavelet-LHL | glszm_SmallAreaEmphasis_wavelet-LHL |
| glszm_SmallAreaHighGrayLevelEmphasis_wavelet-LHL | glszm_SmallAreaLowGrayLevelEmphasis_wavelet-LHL |
| glszm_ZoneEntropy_wavelet-LHL | glszm_ZonePercentage_wavelet-LHL |
| glszm_ZoneVariance_wavelet-LHL | ngtdm_Busyness_wavelet-LHL |
| ngtdm_Coarseness_wavelet-LHL | ngtdm_Complexity_wavelet-LHL |
| ngtdm_Contrast_wavelet-LHL | ngtdm_Strength_wavelet-LHL |
| glcm_Autocorrelation_wavelet-LHH | glcm_JointAverage_wavelet-LHH |
| glcm_ClusterProminence_wavelet-LHH | glcm_ClusterShade_wavelet-LHH |
| glcm_ClusterTendency_wavelet-LHH | glcm_Contrast_wavelet-LHH |
| glcm_Correlation_wavelet-LHH | glcm_DifferenceAverage_wavelet-LHH |
| glcm_DifferenceEntropy_wavelet-LHH | glcm_DifferenceVariance_wavelet-LHH |
| glcm_JointEnergy_wavelet-LHH | glcm_JointEntropy_wavelet-LHH |
| glcm_Imc1_wavelet-LHH | glcm_Imc2_wavelet-LHH |
| glcm_Idm_wavelet-LHH | glcm_Idmn_wavelet-LHH |
| glcm_Id_wavelet-LHH | glcm_Idn_wavelet-LHH |
| glcm_InverseVariance_wavelet-LHH | glcm_MaximumProbability_wavelet-LHH |
| glcm_SumEntropy_wavelet-LHH | gldm_DependenceEntropy_wavelet-LHH |
| gldm_DependenceNonUniformity_wavelet-LHH | gldm_DependenceNonUniformityNormalized_wavelet-LHH |
| gldm_DependenceVariance_wavelet-LHH | gldm_GrayLevelNonUniformity_wavelet-LHH |
| gldm_GrayLevelVariance_wavelet-LHH | gldm_HighGrayLevelEmphasis_wavelet-LHH |
| gldm_LargeDependenceEmphasis_wavelet-LHH | gldm_LargeDependenceHighGrayLevelEmphasis_wavelet-LHH |
| gldm_LargeDependenceLowGrayLevelEmphasis_wavelet-LHH | gldm_LowGrayLevelEmphasis_wavelet-LHH |
| gldm_SmallDependenceEmphasis_wavelet-LHH | gldm_SmallDependenceHighGrayLevelEmphasis_wavelet-LHH |
| gldm_SmallDependenceLowGrayLevelEmphasis_wavelet-LHH | glrlm_GrayLevelNonUniformity_wavelet-LHH |
| glrlm_GrayLevelNonUniformityNormalized_wavelet-LHH | glrlm_GrayLevelVariance_wavelet-LHH |
| glrlm_HighGrayLevelRunEmphasis_wavelet-LHH | glrlm_LongRunEmphasis_wavelet-LHH |
| glrlm_LongRunHighGrayLevelEmphasis_wavelet-LHH | glrlm_LongRunLowGrayLevelEmphasis_wavelet-LHH |
| glrlm_LowGrayLevelRunEmphasis_wavelet-LHH | glrlm_RunEntropy_wavelet-LHH |
| glrlm_RunLengthNonUniformity_wavelet-LHH | glrlm_RunLengthNonUniformityNormalized_wavelet-LHH |
| glrlm_RunPercentage_wavelet-LHH | glrlm_RunVariance_wavelet-LHH |
| glrlm_ShortRunEmphasis_wavelet-LHH | glrlm_ShortRunHighGrayLevelEmphasis_wavelet-LHH |
| glrlm_ShortRunLowGrayLevelEmphasis_wavelet-LHH | glszm_GrayLevelNonUniformity_wavelet-LHH |
| glszm_GrayLevelNonUniformityNormalized_wavelet-LHH | glszm_GrayLevelVariance_wavelet-LHH |
| glszm_HighGrayLevelZoneEmphasis_wavelet-LHH | glszm_LargeAreaEmphasis_wavelet-LHH |
| glszm_LargeAreaHighGrayLevelEmphasis_wavelet-LHH | glszm_LargeAreaLowGrayLevelEmphasis_wavelet-LHH |
| glszm_LowGrayLevelZoneEmphasis_wavelet-LHH | glszm_SizeZoneNonUniformity_wavelet-LHH |
| glszm_SizeZoneNonUniformityNormalized_wavelet-LHH | glszm_SmallAreaEmphasis_wavelet-LHH |
| glszm_SmallAreaHighGrayLevelEmphasis_wavelet-LHH | glszm_SmallAreaLowGrayLevelEmphasis_wavelet-LHH |

| | |
|---|---|
| glszm_ZoneEntropy_wavelet-LHH | glszm_ZonePercentage_wavelet-LHH |
| glszm_ZoneVariance_wavelet-LHH | ngtdm_Busyness_wavelet-LHH |
| ngtdm_Coarseness_wavelet-LHH | ngtdm_Complexity_wavelet-LHH |
| ngtdm_Contrast_wavelet-LHH | ngtdm_Strength_wavelet-LHH |
| glcm_Autocorrelation_wavelet-HLL | glcm_JointAverage_wavelet-HLL |
| glcm_ClusterProminence_wavelet-HLL | glcm_ClusterShade_wavelet-HLL |
| glcm_ClusterTendency_wavelet-HLL | glcm_Contrast_wavelet-HLL |
| glcm_Correlation_wavelet-HLL | glcm_DifferenceAverage_wavelet-HLL |
| glcm_DifferenceEntropy_wavelet-HLL | glcm_DifferenceVariance_wavelet-HLL |
| glcm_JointEnergy_wavelet-HLL | glcm_JointEntropy_wavelet-HLL |
| glcm_Imc1_wavelet-HLL | glcm_Imc2_wavelet-HLL |
| glcm_Idm_wavelet-HLL | glcm_Idmn_wavelet-HLL |
| glcm_Id_wavelet-HLL | glcm_Idn_wavelet-HLL |
| glcm_InverseVariance_wavelet-HLL | glcm_MaximumProbability_wavelet-HLL |
| glcm_SumEntropy_wavelet-HLL | gldm_DependenceEntropy_wavelet-HLL |
| gldm_DependenceNonUniformity_wavelet-HLL | gldm_DependenceNonUniformityNormalized_wavelet-HLL |
| gldm_DependenceVariance_wavelet-HLL | gldm_GrayLevelNonUniformity_wavelet-HLL |
| gldm_GrayLevelVariance_wavelet-HLL | gldm_HighGrayLevelEmphasis_wavelet-HLL |
| gldm_LargeDependenceEmphasis_wavelet-HLL | gldm_LargeDependenceHighGrayLevelEmphasis_wavelet-HLL |
| gldm_LargeDependenceLowGrayLevelEmphasis_wavelet-HLL | gldm_LowGrayLevelEmphasis_wavelet-HLL |
| gldm_SmallDependenceEmphasis_wavelet-HLL | gldm_SmallDependenceHighGrayLevelEmphasis_wavelet-HLL |
| gldm_SmallDependenceLowGrayLevelEmphasis_wavelet-HLL | glrlm_GrayLevelNonUniformity_wavelet-HLL |
| glrlm_GrayLevelNonUniformityNormalized_wavelet-HLL | glrlm_GrayLevelVariance_wavelet-HLL |
| glrlm_HighGrayLevelRunEmphasis_wavelet-HLL | glrlm_LongRunEmphasis_wavelet-HLL |
| glrlm_LongRunHighGrayLevelEmphasis_wavelet-HLL | glrlm_LongRunLowGrayLevelEmphasis_wavelet-HLL |
| glrlm_LowGrayLevelRunEmphasis_wavelet-HLL | glrlm_RunEntropy_wavelet-HLL |
| glrlm_RunLengthNonUniformity_wavelet-HLL | glrlm_RunLengthNonUniformityNormalized_wavelet-HLL |
| glrlm_RunPercentage_wavelet-HLL | glrlm_RunVariance_wavelet-HLL |
| glrlm_ShortRunEmphasis_wavelet-HLL | glrlm_ShortRunHighGrayLevelEmphasis_wavelet-HLL |
| glrlm_ShortRunLowGrayLevelEmphasis_wavelet-HLL | glszm_GrayLevelNonUniformity_wavelet-HLL |
| glszm_GrayLevelNonUniformityNormalized_wavelet-HLL | glszm_GrayLevelVariance_wavelet-HLL |
| glszm_HighGrayLevelZoneEmphasis_wavelet-HLL | glszm_LargeAreaEmphasis_wavelet-HLL |
| glszm_LargeAreaHighGrayLevelEmphasis_wavelet-HLL | glszm_LargeAreaLowGrayLevelEmphasis_wavelet-HLL |
| glszm_LowGrayLevelZoneEmphasis_wavelet-HLL | glszm_SizeZoneNonUniformity_wavelet-HLL |
| glszm_SizeZoneNonUniformityNormalized_wavelet-HLL | glszm_SmallAreaEmphasis_wavelet-HLL |
| glszm_SmallAreaHighGrayLevelEmphasis_wavelet-HLL | glszm_SmallAreaLowGrayLevelEmphasis_wavelet-HLL |
| glszm_ZoneEntropy_wavelet-HLL | glszm_ZonePercentage_wavelet-HLL |
| glszm_ZoneVariance_wavelet-HLL | ngtdm_Busyness_wavelet-HLL |
| ngtdm_Coarseness_wavelet-HLL | ngtdm_Complexity_wavelet-HLL |
| ngtdm_Contrast_wavelet-HLL | ngtdm_Strength_wavelet-HLL |
| glcm_Autocorrelation_wavelet-HLH | glcm_JointAverage_wavelet-HLH |
| glcm_ClusterProminence_wavelet-HLH | glcm_ClusterShade_wavelet-HLH |
| glcm_ClusterTendency_wavelet-HLH | glcm_Contrast_wavelet-HLH |

| | |
|---|---|
| glcm_Correlation_wavelet-HLH | glcm_DifferenceAverage_wavelet-HLH |
| glcm_DifferenceEntropy_wavelet-HLH | glcm_DifferenceVariance_wavelet-HLH |
| glcm_JointEnergy_wavelet-HLH | glcm_JointEntropy_wavelet-HLH |
| glcm_Imc1_wavelet-HLH | glcm_Imc2_wavelet-HLH |
| glcm_Idm_wavelet-HLH | glcm_Idmn_wavelet-HLH |
| glcm_Id_wavelet-HLH | glcm_Idn_wavelet-HLH |
| glcm_InverseVariance_wavelet-HLH | glcm_MaximumProbability_wavelet-HLH |
| glcm_SumEntropy_wavelet-HLH | gldm_DependenceEntropy_wavelet-HLH |
| gldm_DependenceNonUniformity_wavelet-HLH | gldm_DependenceNonUniformityNormalized_wavelet-HLH |
| gldm_DependenceVariance_wavelet-HLH | gldm_GrayLevelNonUniformity_wavelet-HLH |
| gldm_GrayLevelVariance_wavelet-HLH | gldm_HighGrayLevelEmphasis_wavelet-HLH |
| gldm_LargeDependenceEmphasis_wavelet-HLH | gldm_LargeDependenceHighGrayLevelEmphasis_wavelet-HLH |
| gldm_LargeDependenceLowGrayLevelEmphasis_wavelet-HLH | gldm_LowGrayLevelEmphasis_wavelet-HLH |
| gldm_SmallDependenceEmphasis_wavelet-HLH | gldm_SmallDependenceHighGrayLevelEmphasis_wavelet-HLH |
| gldm_SmallDependenceLowGrayLevelEmphasis_wavelet-HLH | glrlm_GrayLevelNonUniformity_wavelet-HLH |
| glrlm_GrayLevelNonUniformityNormalized_wavelet-HLH | glrlm_GrayLevelVariance_wavelet-HLH |
| glrlm_HighGrayLevelRunEmphasis_wavelet-HLH | glrlm_LongRunEmphasis_wavelet-HLH |
| glrlm_LongRunHighGrayLevelEmphasis_wavelet-HLH | glrlm_LongRunLowGrayLevelEmphasis_wavelet-HLH |
| glrlm_LowGrayLevelRunEmphasis_wavelet-HLH | glrlm_RunEntropy_wavelet-HLH |
| glrlm_RunLengthNonUniformity_wavelet-HLH | glrlm_RunLengthNonUniformityNormalized_wavelet-HLH |
| glrlm_RunPercentage_wavelet-HLH | glrlm_RunVariance_wavelet-HLH |
| glrlm_ShortRunEmphasis_wavelet-HLH | glrlm_ShortRunHighGrayLevelEmphasis_wavelet-HLH |
| glrlm_ShortRunLowGrayLevelEmphasis_wavelet-HLH | glszm_GrayLevelNonUniformity_wavelet-HLH |
| glszm_GrayLevelNonUniformityNormalized_wavelet-HLH | glszm_GrayLevelVariance_wavelet-HLH |
| glszm_HighGrayLevelZoneEmphasis_wavelet-HLH | glszm_LargeAreaEmphasis_wavelet-HLH |
| glszm_LargeAreaHighGrayLevelEmphasis_wavelet-HLH | glszm_LargeAreaLowGrayLevelEmphasis_wavelet-HLH |
| glszm_LowGrayLevelZoneEmphasis_wavelet-HLH | glszm_SizeZoneNonUniformity_wavelet-HLH |
| glszm_SizeZoneNonUniformityNormalized_wavelet-HLH | glszm_SmallAreaEmphasis_wavelet-HLH |
| glszm_SmallAreaHighGrayLevelEmphasis_wavelet-HLH | glszm_SmallAreaLowGrayLevelEmphasis_wavelet-HLH |
| glszm_ZoneEntropy_wavelet-HLH | glszm_ZonePercentage_wavelet-HLH |
| glszm_ZoneVariance_wavelet-HLH | ngtdm_Busyness_wavelet-HLH |
| ngtdm_Coarseness_wavelet-HLH | ngtdm_Complexity_wavelet-HLH |
| ngtdm_Contrast_wavelet-HLH | ngtdm_Strength_wavelet-HLH |
| glcm_Autocorrelation_wavelet-HHL | glcm_JointAverage_wavelet-HHL |
| glcm_ClusterProminence_wavelet-HHL | glcm_ClusterShade_wavelet-HHL |
| glcm_ClusterTendency_wavelet-HHL | glcm_Contrast_wavelet-HHL |
| glcm_Correlation_wavelet-HHL | glcm_DifferenceAverage_wavelet-HHL |
| glcm_DifferenceEntropy_wavelet-HHL | glcm_DifferenceVariance_wavelet-HHL |
| glcm_JointEnergy_wavelet-HHL | glcm_JointEntropy_wavelet-HHL |
| glcm_Imc1_wavelet-HHL | glcm_Imc2_wavelet-HHL |
| glcm_Idm_wavelet-HHL | glcm_Idmn_wavelet-HHL |
| glcm_Id_wavelet-HHL | glcm_Idn_wavelet-HHL |
| glcm_InverseVariance_wavelet-HHL | glcm_MaximumProbability_wavelet-HHL |

| | |
|---|---|
| glcm_SumEntropy_wavelet-HHL | gldm_DependenceEntropy_wavelet-HHL |
| gldm_DependenceNonUniformity_wavelet-HHL | gldm_DependenceNonUniformityNormalized_wavelet-HHL |
| gldm_DependenceVariance_wavelet-HHL | gldm_GrayLevelNonUniformity_wavelet-HHL |
| gldm_GrayLevelVariance_wavelet-HHL | gldm_HighGrayLevelEmphasis_wavelet-HHL |
| gldm_LargeDependenceEmphasis_wavelet-HHL | gldm_LargeDependenceHighGrayLevelEmphasis_wavelet-HHL |
| gldm_LargeDependenceLowGrayLevelEmphasis_wavelet-HHL | gldm_LowGrayLevelEmphasis_wavelet-HHL |
| gldm_SmallDependenceEmphasis_wavelet-HHL | gldm_SmallDependenceHighGrayLevelEmphasis_wavelet-HHL |
| gldm_SmallDependenceLowGrayLevelEmphasis_wavelet-HHL | glrlm_GrayLevelNonUniformity_wavelet-HHL |
| glrlm_GrayLevelNonUniformityNormalized_wavelet-HHL | glrlm_GrayLevelVariance_wavelet-HHL |
| glrlm_HighGrayLevelRunEmphasis_wavelet-HHL | glrlm_LongRunEmphasis_wavelet-HHL |
| glrlm_LongRunHighGrayLevelEmphasis_wavelet-HHL | glrlm_LongRunLowGrayLevelEmphasis_wavelet-HHL |
| glrlm_LowGrayLevelRunEmphasis_wavelet-HHL | glrlm_RunEntropy_wavelet-HHL |
| glrlm_RunLengthNonUniformity_wavelet-HHL | glrlm_RunLengthNonUniformityNormalized_wavelet-HHL |
| glrlm_RunPercentage_wavelet-HHL | glrlm_RunVariance_wavelet-HHL |
| glrlm_ShortRunEmphasis_wavelet-HHL | glrlm_ShortRunHighGrayLevelEmphasis_wavelet-HHL |
| glrlm_ShortRunLowGrayLevelEmphasis_wavelet-HHL | glszm_GrayLevelNonUniformity_wavelet-HHL |
| glszm_GrayLevelNonUniformityNormalized_wavelet-HHL | glszm_GrayLevelVariance_wavelet-HHL |
| glszm_HighGrayLevelZoneEmphasis_wavelet-HHL | glszm_LargeAreaEmphasis_wavelet-HHL |
| glszm_LargeAreaHighGrayLevelEmphasis_wavelet-HHL | glszm_LargeAreaLowGrayLevelEmphasis_wavelet-HHL |
| glszm_LowGrayLevelZoneEmphasis_wavelet-HHL | glszm_SizeZoneNonUniformity_wavelet-HHL |
| glszm_SizeZoneNonUniformityNormalized_wavelet-HHL | glszm_SmallAreaEmphasis_wavelet-HHL |
| glszm_SmallAreaHighGrayLevelEmphasis_wavelet-HHL | glszm_SmallAreaLowGrayLevelEmphasis_wavelet-HHL |
| glszm_ZoneEntropy_wavelet-HHL | glszm_ZonePercentage_wavelet-HHL |
| glszm_ZoneVariance_wavelet-HHL | ngtdm_Busyness_wavelet-HHL |
| ngtdm_Coarseness_wavelet-HHL | ngtdm_Complexity_wavelet-HHL |
| ngtdm_Contrast_wavelet-HHL | ngtdm_Strength_wavelet-HHL |
| glcm_Autocorrelation_wavelet-HHH | glcm_JointAverage_wavelet-HHH |
| glcm_ClusterProminence_wavelet-HHH | glcm_ClusterShade_wavelet-HHH |
| glcm_ClusterTendency_wavelet-HHH | glcm_Contrast_wavelet-HHH |
| glcm_Correlation_wavelet-HHH | glcm_DifferenceAverage_wavelet-HHH |
| glcm_DifferenceEntropy_wavelet-HHH | glcm_DifferenceVariance_wavelet-HHH |
| glcm_JointEnergy_wavelet-HHH | glcm_JointEntropy_wavelet-HHH |
| glcm_Imc1_wavelet-HHH | glcm_Imc2_wavelet-HHH |
| glcm_Idm_wavelet-HHH | glcm_Idmn_wavelet-HHH |
| glcm_Id_wavelet-HHH | glcm_Idn_wavelet-HHH |
| glcm_InverseVariance_wavelet-HHH | glcm_MaximumProbability_wavelet-HHH |
| glcm_SumEntropy_wavelet-HHH | gldm_DependenceEntropy_wavelet-HHH |
| gldm_DependenceNonUniformity_wavelet-HHH | gldm_DependenceNonUniformityNormalized_wavelet-HHH |
| gldm_DependenceVariance_wavelet-HHH | gldm_GrayLevelNonUniformity_wavelet-HHH |
| gldm_GrayLevelVariance_wavelet-HHH | gldm_HighGrayLevelEmphasis_wavelet-HHH |
| gldm_LargeDependenceEmphasis_wavelet-HHH | gldm_LargeDependenceHighGrayLevelEmphasis_wavelet-HHH |
| gldm_LargeDependenceLowGrayLevelEmphasis_wavelet-HHH | gldm_LowGrayLevelEmphasis_wavelet-HHH |
| gldm_SmallDependenceEmphasis_wavelet-HHH | gldm_SmallDependenceHighGrayLevelEmphasis_wavelet-HHH |

| | |
|---|---|
| gldm_SmallDependenceLowGrayLevelEmphasis_wavelet-HHH | glrlm_GrayLevelNonUniformity_wavelet-HHH |
| glrlm_GrayLevelNonUniformityNormalized_wavelet-HHH | glrlm_GrayLevelVariance_wavelet-HHH |
| glrlm_HighGrayLevelRunEmphasis_wavelet-HHH | glrlm_LongRunEmphasis_wavelet-HHH |
| glrlm_LongRunHighGrayLevelEmphasis_wavelet-HHH | glrlm_LongRunLowGrayLevelEmphasis_wavelet-HHH |
| glrlm_LowGrayLevelRunEmphasis_wavelet-HHH | glrlm_RunEntropy_wavelet-HHH |
| glrlm_RunLengthNonUniformity_wavelet-HHH | glrlm_RunLengthNonUniformityNormalized_wavelet-HHH |
| glrlm_RunPercentage_wavelet-HHH | glrlm_RunVariance_wavelet-HHH |
| glrlm_ShortRunEmphasis_wavelet-HHH | glrlm_ShortRunHighGrayLevelEmphasis_wavelet-HHH |
| glrlm_ShortRunLowGrayLevelEmphasis_wavelet-HHH | glszm_GrayLevelNonUniformity_wavelet-HHH |
| glszm_GrayLevelNonUniformityNormalized_wavelet-HHH | glszm_GrayLevelVariance_wavelet-HHH |
| glszm_HighGrayLevelZoneEmphasis_wavelet-HHH | glszm_LargeAreaEmphasis_wavelet-HHH |
| glszm_LargeAreaHighGrayLevelEmphasis_wavelet-HHH | glszm_LargeAreaLowGrayLevelEmphasis_wavelet-HHH |
| glszm_LowGrayLevelZoneEmphasis_wavelet-HHH | glszm_SizeZoneNonUniformity_wavelet-HHH |
| glszm_SizeZoneNonUniformityNormalized_wavelet-HHH | glszm_SmallAreaEmphasis_wavelet-HHH |
| glszm_SmallAreaHighGrayLevelEmphasis_wavelet-HHH | glszm_SmallAreaLowGrayLevelEmphasis_wavelet-HHH |
| glszm_ZoneEntropy_wavelet-HHH | glszm_ZonePercentage_wavelet-HHH |
| glszm_ZoneVariance_wavelet-HHH | ngtdm_Busyness_wavelet-HHH |
| ngtdm_Coarseness_wavelet-HHH | ngtdm_Complexity_wavelet-HHH |
| ngtdm_Contrast_wavelet-HHH | ngtdm_Strength_wavelet-HHH |
| firstorder_10Percentile_wavelet-LLL | firstorder_90Percentile_wavelet-LLL |
| firstorder_Energy_wavelet-LLL | firstorder_Entropy_wavelet-LLL |
| firstorder_InterquartileRange_wavelet-LLL | firstorder_Kurtosis_wavelet-LLL |
| firstorder_Maximum_wavelet-LLL | firstorder_MeanAbsoluteDeviation_wavelet-LLL |
| firstorder_Mean_wavelet-LLL | firstorder_Median_wavelet-LLL |
| firstorder_Minimum_wavelet-LLL | firstorder_Range_wavelet-LLL |
| firstorder_RobustMeanAbsoluteDeviation_wavelet-LLL | firstorder_RootMeanSquared_wavelet-LLL |
| firstorder_Skewness_wavelet-LLL | firstorder_TotalEnergy_wavelet-LLL |
| firstorder_Uniformity_wavelet-LLL | firstorder_Variance_wavelet-LLL |
| glcm_Autocorrelation_wavelet-LLL | glcm_JointAverage_wavelet-LLL |
| glcm_ClusterProminence_wavelet-LLL | glcm_ClusterShade_wavelet-LLL |
| glcm_ClusterTendency_wavelet-LLL | glcm_Contrast_wavelet-LLL |
| glcm_Correlation_wavelet-LLL | glcm_DifferenceAverage_wavelet-LLL |
| glcm_DifferenceEntropy_wavelet-LLL | glcm_DifferenceVariance_wavelet-LLL |
| glcm_JointEnergy_wavelet-LLL | glcm_JointEntropy_wavelet-LLL |
| glcm_Imc1_wavelet-LLL | glcm_Imc2_wavelet-LLL |
| glcm_Idm_wavelet-LLL | glcm_Idmn_wavelet-LLL |
| glcm_Id_wavelet-LLL | glcm_Idn_wavelet-LLL |
| glcm_InverseVariance_wavelet-LLL | glcm_MaximumProbability_wavelet-LLL |
| glcm_SumEntropy_wavelet-LLL | gldm_DependenceEntropy_wavelet-LLL |
| gldm_DependenceNonUniformity_wavelet-LLL | gldm_DependenceNonUniformityNormalized_wavelet-LLL |
| gldm_DependenceVariance_wavelet-LLL | gldm_GrayLevelNonUniformity_wavelet-LLL |
| gldm_GrayLevelVariance_wavelet-LLL | gldm_HighGrayLevelEmphasis_wavelet-LLL |
| gldm_LargeDependenceEmphasis_wavelet-LLL | gldm_LargeDependenceHighGrayLevelEmphasis_wavelet-LLL |

| | |
|---|---|
| gldm_LargeDependenceLowGrayLevelEmphasis_wavelet-LLL | gldm_LowGrayLevelEmphasis_wavelet-LLL |
| gldm_SmallDependenceEmphasis_wavelet-LLL | gldm_SmallDependenceHighGrayLevelEmphasis_wavelet-LLL |
| gldm_SmallDependenceLowGrayLevelEmphasis_wavelet-LLL | glrlm_GrayLevelNonUniformity_wavelet-LLL |
| glrlm_GrayLevelNonUniformityNormalized_wavelet-LLL | glrlm_GrayLevelVariance_wavelet-LLL |
| glrlm_HighGrayLevelRunEmphasis_wavelet-LLL | glrlm_LongRunEmphasis_wavelet-LLL |
| glrlm_LongRunHighGrayLevelEmphasis_wavelet-LLL | glrlm_LongRunLowGrayLevelEmphasis_wavelet-LLL |
| glrlm_LowGrayLevelRunEmphasis_wavelet-LLL | glrlm_RunEntropy_wavelet-LLL |
| glrlm_RunLengthNonUniformity_wavelet-LLL | glrlm_RunLengthNonUniformityNormalized_wavelet-LLL |
| glrlm_RunPercentage_wavelet-LLL | glrlm_RunVariance_wavelet-LLL |
| glrlm_ShortRunEmphasis_wavelet-LLL | glrlm_ShortRunHighGrayLevelEmphasis_wavelet-LLL |
| glrlm_ShortRunLowGrayLevelEmphasis_wavelet-LLL | glszm_GrayLevelNonUniformity_wavelet-LLL |
| glszm_GrayLevelNonUniformityNormalized_wavelet-LLL | glszm_GrayLevelVariance_wavelet-LLL |
| glszm_HighGrayLevelZoneEmphasis_wavelet-LLL | glszm_LargeAreaEmphasis_wavelet-LLL |
| glszm_LargeAreaHighGrayLevelEmphasis_wavelet-LLL | glszm_LargeAreaLowGrayLevelEmphasis_wavelet-LLL |
| glszm_LowGrayLevelZoneEmphasis_wavelet-LLL | glszm_SizeZoneNonUniformity_wavelet-LLL |
| glszm_SizeZoneNonUniformityNormalized_wavelet-LLL | glszm_SmallAreaEmphasis_wavelet-LLL |
| glszm_SmallAreaHighGrayLevelEmphasis_wavelet-LLL | glszm_SmallAreaLowGrayLevelEmphasis_wavelet-LLL |
| glszm_ZoneEntropy_wavelet-LLL | glszm_ZonePercentage_wavelet-LLL |
| glszm_ZoneVariance_wavelet-LLL | ngtdm_Busyness_wavelet-LLL |
| ngtdm_Coarseness_wavelet-LLL | ngtdm_Complexity_wavelet-LLL |
| ngtdm_Contrast_wavelet-LLL | ngtdm_Strength_wavelet-LLL |
| glcm_Autocorrelation_square | glcm_JointAverage_square |
| glcm_ClusterProminence_square | glcm_ClusterShade_square |
| glcm_ClusterTendency_square | glcm_Contrast_square |
| glcm_Correlation_square | glcm_DifferenceAverage_square |
| glcm_DifferenceEntropy_square | glcm_DifferenceVariance_square |
| glcm_JointEnergy_square | glcm_JointEntropy_square |
| glcm_Imc1_square | glcm_Imc2_square |
| glcm_Idm_square | glcm_Idmn_square |
| glcm_Id_square | glcm_Idn_square |
| glcm_InverseVariance_square | glcm_MaximumProbability_square |
| glcm_SumEntropy_square | gldm_DependenceEntropy_square |
| gldm_DependenceNonUniformity_square | gldm_DependenceNonUniformityNormalized_square |
| gldm_DependenceVariance_square | gldm_GrayLevelNonUniformity_square |
| gldm_GrayLevelVariance_square | gldm_HighGrayLevelEmphasis_square |
| gldm_LargeDependenceEmphasis_square | gldm_LargeDependenceHighGrayLevelEmphasis_square |
| gldm_LargeDependenceLowGrayLevelEmphasis_square | gldm_LowGrayLevelEmphasis_square |
| gldm_SmallDependenceEmphasis_square | gldm_SmallDependenceHighGrayLevelEmphasis_square |
| gldm_SmallDependenceLowGrayLevelEmphasis_square | glrlm_GrayLevelNonUniformity_square |
| glrlm_GrayLevelNonUniformityNormalized_square | glrlm_GrayLevelVariance_square |
| glrlm_HighGrayLevelRunEmphasis_square | glrlm_LongRunEmphasis_square |
| glrlm_LongRunHighGrayLevelEmphasis_square | glrlm_LongRunLowGrayLevelEmphasis_square |
| glrlm_LowGrayLevelRunEmphasis_square | glrlm_RunEntropy_square |

| | |
|---|---|
| glrlm_RunLengthNonUniformity_square | glrlm_RunLengthNonUniformityNormalized_square |
| glrlm_RunPercentage_square | glrlm_RunVariance_square |
| glrlm_ShortRunEmphasis_square | glrlm_ShortRunHighGrayLevelEmphasis_square |
| glrlm_ShortRunLowGrayLevelEmphasis_square | glszm_GrayLevelNonUniformity_square |
| glszm_GrayLevelNonUniformityNormalized_square | glszm_GrayLevelVariance_square |
| glszm_HighGrayLevelZoneEmphasis_square | glszm_LargeAreaEmphasis_square |
| glszm_LargeAreaHighGrayLevelEmphasis_square | glszm_LargeAreaLowGrayLevelEmphasis_square |
| glszm_LowGrayLevelZoneEmphasis_square | glszm_SizeZoneNonUniformity_square |
| glszm_SizeZoneNonUniformityNormalized_square | glszm_SmallAreaEmphasis_square |
| glszm_SmallAreaHighGrayLevelEmphasis_square | glszm_SmallAreaLowGrayLevelEmphasis_square |
| glszm_ZoneEntropy_square | glszm_ZonePercentage_square |
| glszm_ZoneVariance_square | ngtdm_Busyness_square |
| ngtdm_Coarseness_square | ngtdm_Complexity_square |
| ngtdm_Contrast_square | ngtdm_Strength_square |
| glcm_Autocorrelation_squareroot | glcm_JointAverage_squareroot |
| glcm_ClusterProminence_squareroot | glcm_ClusterShade_squareroot |
| glcm_ClusterTendency_squareroot | glcm_Contrast_squareroot |
| glcm_Correlation_squareroot | glcm_DifferenceAverage_squareroot |
| glcm_DifferenceEntropy_squareroot | glcm_DifferenceVariance_squareroot |
| glcm_JointEnergy_squareroot | glcm_JointEntropy_squareroot |
| glcm_Imc1_squareroot | glcm_Imc2_squareroot |
| glcm_Idm_squareroot | glcm_Idmn_squareroot |
| glcm_Id_squareroot | glcm_Idn_squareroot |
| glcm_InverseVariance_squareroot | glcm_MaximumProbability_squareroot |
| glcm_SumEntropy_squareroot | gldm_DependenceEntropy_squareroot |
| gldm_DependenceNonUniformity_squareroot | gldm_DependenceNonUniformityNormalized_squareroot |
| gldm_DependenceVariance_squareroot | gldm_GrayLevelNonUniformity_squareroot |
| gldm_GrayLevelVariance_squareroot | gldm_HighGrayLevelEmphasis_squareroot |
| gldm_LargeDependenceEmphasis_squareroot | gldm_LargeDependenceHighGrayLevelEmphasis_squareroot |
| gldm_LargeDependenceLowGrayLevelEmphasis_squareroot | gldm_LowGrayLevelEmphasis_squareroot |
| gldm_SmallDependenceEmphasis_squareroot | gldm_SmallDependenceHighGrayLevelEmphasis_squareroot |
| gldm_SmallDependenceLowGrayLevelEmphasis_squareroot | glrlm_GrayLevelNonUniformity_squareroot |
| glrlm_GrayLevelNonUniformityNormalized_squareroot | glrlm_GrayLevelVariance_squareroot |
| glrlm_HighGrayLevelRunEmphasis_squareroot | glrlm_LongRunEmphasis_squareroot |
| glrlm_LongRunHighGrayLevelEmphasis_squareroot | glrlm_LongRunLowGrayLevelEmphasis_squareroot |
| glrlm_LowGrayLevelRunEmphasis_squareroot | glrlm_RunEntropy_squareroot |
| glrlm_RunLengthNonUniformity_squareroot | glrlm_RunLengthNonUniformityNormalized_squareroot |
| glrlm_RunPercentage_squareroot | glrlm_RunVariance_squareroot |
| glrlm_ShortRunEmphasis_squareroot | glrlm_ShortRunHighGrayLevelEmphasis_squareroot |
| glrlm_ShortRunLowGrayLevelEmphasis_squareroot | glszm_GrayLevelNonUniformity_squareroot |
| glszm_GrayLevelNonUniformityNormalized_squareroot | glszm_GrayLevelVariance_squareroot |
| glszm_HighGrayLevelZoneEmphasis_squareroot | glszm_LargeAreaEmphasis_squareroot |
| glszm_LargeAreaHighGrayLevelEmphasis_squareroot | glszm_LargeAreaLowGrayLevelEmphasis_squareroot |

| | |
|---|---|
| glszm_LowGrayLevelZoneEmphasis_squareroot | glszm_SizeZoneNonUniformity_squareroot |
| glszm_SizeZoneNonUniformityNormalized_squareroot | glszm_SmallAreaEmphasis_squareroot |
| glszm_SmallAreaHighGrayLevelEmphasis_squareroot | glszm_SmallAreaLowGrayLevelEmphasis_squareroot |
| glszm_ZoneEntropy_squareroot | glszm_ZonePercentage_squareroot |
| glszm_ZoneVariance_squareroot | ngtdm_Busyness_squareroot |
| ngtdm_Coarseness_squareroot | ngtdm_Complexity_squareroot |
| ngtdm_Contrast_squareroot | ngtdm_Strength_squareroot |
| glcm_Autocorrelation_logarithm | glcm_JointAverage_logarithm |
| glcm_ClusterProminence_logarithm | glcm_ClusterShade_logarithm |
| glcm_ClusterTendency_logarithm | glcm_Contrast_logarithm |
| glcm_Correlation_logarithm | glcm_DifferenceAverage_logarithm |
| glcm_DifferenceEntropy_logarithm | glcm_DifferenceVariance_logarithm |
| glcm_JointEnergy_logarithm | glcm_JointEntropy_logarithm |
| glcm_Imc1_logarithm | glcm_Imc2_logarithm |
| glcm_Idm_logarithm | glcm_Idmn_logarithm |
| glcm_Id_logarithm | glcm_Idn_logarithm |
| glcm_InverseVariance_logarithm | glcm_MaximumProbability_logarithm |
| glcm_SumEntropy_logarithm | gldm_DependenceEntropy_logarithm |
| gldm_DependenceNonUniformity_logarithm | gldm_DependenceNonUniformityNormalized_logarithm |
| gldm_DependenceVariance_logarithm | gldm_GrayLevelNonUniformity_logarithm |
| gldm_GrayLevelVariance_logarithm | gldm_HighGrayLevelEmphasis_logarithm |
| gldm_LargeDependenceEmphasis_logarithm | gldm_LargeDependenceHighGrayLevelEmphasis_logarithm |
| gldm_LargeDependenceLowGrayLevelEmphasis_logarithm | gldm_LowGrayLevelEmphasis_logarithm |
| gldm_SmallDependenceEmphasis_logarithm | gldm_SmallDependenceHighGrayLevelEmphasis_logarithm |
| gldm_SmallDependenceLowGrayLevelEmphasis_logarithm | glrlm_GrayLevelNonUniformity_logarithm |
| glrlm_GrayLevelNonUniformityNormalized_logarithm | glrlm_GrayLevelVariance_logarithm |
| glrlm_HighGrayLevelRunEmphasis_logarithm | glrlm_LongRunEmphasis_logarithm |
| glrlm_LongRunHighGrayLevelEmphasis_logarithm | glrlm_LongRunLowGrayLevelEmphasis_logarithm |
| glrlm_LowGrayLevelRunEmphasis_logarithm | glrlm_RunEntropy_logarithm |
| glrlm_RunLengthNonUniformity_logarithm | glrlm_RunLengthNonUniformityNormalized_logarithm |
| glrlm_RunPercentage_logarithm | glrlm_RunVariance_logarithm |
| glrlm_ShortRunEmphasis_logarithm | glrlm_ShortRunHighGrayLevelEmphasis_logarithm |
| glrlm_ShortRunLowGrayLevelEmphasis_logarithm | glszm_GrayLevelNonUniformity_logarithm |
| glszm_GrayLevelNonUniformityNormalized_logarithm | glszm_GrayLevelVariance_logarithm |
| glszm_HighGrayLevelZoneEmphasis_logarithm | glszm_LargeAreaEmphasis_logarithm |
| glszm_LargeAreaHighGrayLevelEmphasis_logarithm | glszm_LargeAreaLowGrayLevelEmphasis_logarithm |
| glszm_LowGrayLevelZoneEmphasis_logarithm | glszm_SizeZoneNonUniformity_logarithm |
| glszm_SizeZoneNonUniformityNormalized_logarithm | glszm_SmallAreaEmphasis_logarithm |
| glszm_SmallAreaHighGrayLevelEmphasis_logarithm | glszm_SmallAreaLowGrayLevelEmphasis_logarithm |
| glszm_ZoneEntropy_logarithm | glszm_ZonePercentage_logarithm |
| glszm_ZoneVariance_logarithm | ngtdm_Busyness_logarithm |
| ngtdm_Coarseness_logarithm | ngtdm_Complexity_logarithm |
| ngtdm_Contrast_logarithm | ngtdm_Strength_logarithm |

| | |
|---|---|
| firstorder_10Percentile_exponential | firstorder_90Percentile_exponential |
| firstorder_Energy_exponential | firstorder_Entropy_exponential |
| firstorder_InterquartileRange_exponential | firstorder_Kurtosis_exponential |
| firstorder_Maximum_exponential | firstorder_MeanAbsoluteDeviation_exponential |
| firstorder_Mean_exponential | firstorder_Median_exponential |
| firstorder_Minimum_exponential | firstorder_Range_exponential |
| firstorder_RobustMeanAbsoluteDeviation_exponential | firstorder_RootMeanSquared_exponential |
| firstorder_Skewness_exponential | firstorder_TotalEnergy_exponential |
| firstorder_Uniformity_exponential | firstorder_Variance_exponential |
| glcm_Autocorrelation_exponential | glcm_JointAverage_exponential |
| glcm_ClusterProminence_exponential | glcm_ClusterShade_exponential |
| glcm_ClusterTendency_exponential | glcm_Contrast_exponential |
| glcm_Correlation_exponential | glcm_DifferenceAverage_exponential |
| glcm_DifferenceEntropy_exponential | glcm_DifferenceVariance_exponential |
| glcm_JointEnergy_exponential | glcm_JointEntropy_exponential |
| glcm_Imc1_exponential | glcm_Imc2_exponential |
| glcm_Idm_exponential | glcm_Idmn_exponential |
| glcm_Id_exponential | glcm_Idn_exponential |
| glcm_InverseVariance_exponential | glcm_MaximumProbability_exponential |
| glcm_SumEntropy_exponential | gldm_DependenceEntropy_exponential |
| gldm_DependenceNonUniformity_exponential | gldm_DependenceNonUniformityNormalized_exponential |
| gldm_DependenceVariance_exponential | gldm_GrayLevelNonUniformity_exponential |
| gldm_GrayLevelVariance_exponential | gldm_HighGrayLevelEmphasis_exponential |
| gldm_LargeDependenceEmphasis_exponential | gldm_LargeDependenceHighGrayLevelEmphasis_exponential |
| gldm_LargeDependenceLowGrayLevelEmphasis_exponential | gldm_LowGrayLevelEmphasis_exponential |
| gldm_SmallDependenceEmphasis_exponential | gldm_SmallDependenceHighGrayLevelEmphasis_exponential |
| gldm_SmallDependenceLowGrayLevelEmphasis_exponential | glrlm_GrayLevelNonUniformity_exponential |
| glrlm_GrayLevelNonUniformityNormalized_exponential | glrlm_GrayLevelVariance_exponential |
| glrlm_HighGrayLevelRunEmphasis_exponential | glrlm_LongRunEmphasis_exponential |
| glrlm_LongRunHighGrayLevelEmphasis_exponential | glrlm_LongRunLowGrayLevelEmphasis_exponential |
| glrlm_LowGrayLevelRunEmphasis_exponential | glrlm_RunEntropy_exponential |
| glrlm_RunLengthNonUniformity_exponential | glrlm_RunLengthNonUniformityNormalized_exponential |
| glrlm_RunPercentage_exponential | glrlm_RunVariance_exponential |
| glrlm_ShortRunEmphasis_exponential | glrlm_ShortRunHighGrayLevelEmphasis_exponential |
| glrlm_ShortRunLowGrayLevelEmphasis_exponential | glszm_GrayLevelNonUniformity_exponential |
| glszm_GrayLevelNonUniformityNormalized_exponential | glszm_GrayLevelVariance_exponential |
| glszm_HighGrayLevelZoneEmphasis_exponential | glszm_LargeAreaEmphasis_exponential |
| glszm_LargeAreaHighGrayLevelEmphasis_exponential | glszm_LargeAreaLowGrayLevelEmphasis_exponential |
| glszm_LowGrayLevelZoneEmphasis_exponential | glszm_SizeZoneNonUniformity_exponential |
| glszm_SizeZoneNonUniformityNormalized_exponential | glszm_SmallAreaEmphasis_exponential |
| glszm_SmallAreaHighGrayLevelEmphasis_exponential | glszm_SmallAreaLowGrayLevelEmphasis_exponential |
| glszm_ZoneEntropy_exponential | glszm_ZonePercentage_exponential |
| glszm_ZoneVariance_exponential | ngtdm_Busyness_exponential |

| | |
|---|---|
| ngtdm_Coarseness_exponential | ngtdm_Complexity_exponential |
| ngtdm_Contrast_exponential | ngtdm_Strength_exponential |
